# Is there a role for traditional and complementary medicines in managing chronic fatigue? a systematic review of randomized controlled trials

**DOI:** 10.3389/fphar.2023.1266803

**Published:** 2023-10-24

**Authors:** Yuxiao Li, Jingya Yang, Chi Ian Chau, Junnan Shi, Xianwen Chen, Hao Hu, Carolina Oi Lam Ung

**Affiliations:** ^1^ State Key Laboratory of Quality Research in Chinese Medicine, Institute of Chinese Medical Sciences, University of Macau, Macao SAR, China; ^2^ Department of Public Health and Medicinal Administration, Faculty of Health Sciences, University of Macau, Macao SAR, China

**Keywords:** chronic fatigue syndrome, traditional and complementary medicine, randomized clinical trial, CONSORT-CHM, risk of bias

## Abstract

**Introduction:** Chronic fatigue syndrome (CFS) is an increasingly common condition that is challenging to treat due to unclear etiology and a lack of consensus on clinical diagnosis and treatment guidance. Many affected people resorted to using traditional and complementary medicines (T&CMs). However, the evidence for T&CMs for CFS has been inconclusive and continues to evolve. The study aims to identify, summarize and assess the most recent evidence on the efficacy and safety of T&CMs for CFS.

**Methods:** Randomized controlled trials (RCTs) investigating T&CMs for CFS published in English of Chinese between 1 January 2013 and 31 December 2022 were searched from 7 databases. RCTs comparing T&CMs with no treatment, placebo, or pharmacological medicine were included, irrespective of language or blinding. The Consolidated Standards of Reporting Trials Statement extensions for Chinese herbal medicine Formulas (CONSORT-CHM) and the Cochrane Collaboration’s Risk of Bias tool were used to evaluate the quality and risk of bias of included studies.

**Results:** A total of 62 RCTs investigating 43 types of T&CMs and involving 5,231 participants with CFS were included in this review. The primary outcome measures mainly included the scoring of fatigue symptoms using the validated tool Fatigue Scale-14 (FS-14) or the TCM syndrome score. The main interventions showing overall efficacy were Chaihu Guizhi Decoction and Buzhong Yiqi combined with Xiao Chaihu Decoction, and 148 ingredients were identified, including Astragali Radix, Glycyrrhizae Radix et Rhizoma, Atractylodis Macrocephalae Rhizoma, and Bupleuri Radix. The most significant effect was the improvement of fatigue, followed by TCM-diagnosed symptoms and other psychological conditions. No serious adverse effect had been reported. However, the quality of the RCTs included RCTs were found to be suboptimal, and the risk of bias remained uncertain.

**Conclusion:** Some evidence from RCTs supported the efficacy and safety of T&CM in CFS. However, given the methodological and quality heterogenicity of the included studies, the recommendations of T&CMs in treating CFS remain inconclusive. To develop better quality evidence about T&CMs for CFS, future studies should employ more objective diagnosis standards and outcome measurements, larger sample size, and better bias control, and ensure the compliance with the corresponding reporting guidelines.

**Systematic Review Registration:** https://www.crd.york.ac.uk/prospero/display_record.php?ID=CRD42022362268, identifier CRD42022362268.

## 1 Introduction

Chronic fatigue syndrome (CFS) is a chronic, debilitating disorder characterized by unexplained fatigue lasting more than 6 months with complex cognitive, immune, endocrine, and autonomic dysfunction-related symptoms (Son, 2019; vant Leven et al., 2010), such as muscle and joint pain, headache, sore throat, lymph node tenderness, sleep deficiency, and cognitive or behavioral impairment (Vercoulen et al., 1994; Carruthers et al., 2011a; Brurberg et al., 2014). Unlike acute fatigue which usually settles upon sufficient rest or alleviation of underlying causes, this chronic exhaustion and weakness is exceptionally problematic as it negatively impacts on individual physical and social functioning, with a huge burden on the patient’s family and caregivers, while also having negative social and economic implications (Matura et al., 2018).

In the United States (Jason et al., 1999), the prevalence of CFS is approximately 1% and the unemployment rate for people with this pathological condition is between 35%–69% (Beyond Myalgic Encephalomyelitis/Chronic Fatigue Syndrome, 2015). In a review reported by The Institute of Medicine (IOM), it was estimated that 836,000 to 2.5 million people in the United States suffered from CFS, resulting in financial costs between 17–24 billion (USD) annually (Collaborators, 2019; Cortes Rivera et al., 2019), while the cost in the United Kingdom (UK) was around 1,906 (GBP) per person every 3 months (McCrone et al., 2003). Other studies had shown that the prevalence of the disease varied globally, with 0.77% and 0.76% in Korea and Japan respectively. Recent systematic reviews or meta-analyses had produced estimates of the prevalence of CFS that ranged from 0.76% to 3.28%. This heterogeneity was due to the different sets of criteria that identified overlapping populations with slightly different symptom characteristics.

Despite the high prevalence, the gaps in the effective management of CFS remain. Firstly, the awareness of CFS lagged behind. It has been reported that up to 90% of people with CFS remained undiagnosed or misdiagnosed as other conditions (Cho et al., 2008; Brimmer et al., 2010). Secondly, the diagnosis of CFS is complicated. For instance, according to the Canadian Consensus Criteria (CCC) (Carruthers et al., 2011b), the criteria for CFS are multifaceted including: (a) post-exertional malaise and/or fatigue, sleep dysfunction and pain; (b) having two or more neurological/cognitive manifestations and one or more symptoms from two of the categories of autonomic, neuroendocrine and immune manifestations; and (c) with the illness persisting for at least 6 months. As chronic fatigue represents a cluster of non-specific signs and symptoms, there is no single test to confirm a diagnosis and clinical diagnostic criterion continues to evolve (Bateman et al., 2021). At present, the diagnosis of chronic fatigue is usually confirmed following elimination of other possible causes of similar symptoms. Thirdly, various case definitions or diagnostic criteria currently exist for CFS including the toolkits based on the 1994 definition of the Center for Disease Control criteria (CDC) (Fukuda et al., 1994), and the Introduction to International Consensus (ICC) (Carruthers et al., 2011a), Holmes (Holmes et al., 1988), Oxford (Sharpe et al., 1991), epidemiological case definition (ECD) (Osoba et al., 2011), and National Institute for Health and Care Excellence (NICE) guidelines (National Institute for Health and Care Excellence, 2021). What these diagnostic criteria have in common was the recommendation of a comprehensive and detailed examination of the patient’s symptoms, laboratory and further investigations that helped in differential diagnosis. But they all differ in terms of specific description of the main and accompanied diseases, as well as important symptoms of chronic fatigue (Johnston et al., 2014).

Even with an accurate diagnosis, the evidence for appropriate management of chronic fatigue remains controversial and limited (Cho et al., 2009; Zhang et al., 2022). No medications for the treatment of CFS had been approved by the U.S Food and Drug Administration (FDA). However, many drugs (e.g., rintatolimod, an immune modulator) were used for CFS without review and approval (off-label) (Smith et al., 2015). In an FDA survey, treatments for patients with CFS were divided into two broad categories including the treatments that targeted the cause of the disease, including immunomodulators, antivirals, and antibiotics, or the treatment that targeted specific symptoms or perpetuating factors, including medications or non-pharmacological treatments (such as yoga, motor skills, counseling, pacing strategies, and mental exercises) to treat specific symptoms such as pain, fatigue, autonomic dysfunction, and sleep dysfunction (CfDEa, 2015). However, conventional medicines may be prescribed only to achieve symptomatic relief (Castro-Marrero et al., 2017; Joung et al., 2019) but the risk of adverse effects is often concerning. Cognitive-behavioral therapy (CBT) and graded exercise therapy (GET), which were once commonly recommended for CFS, are now found to possibly worsen the condition (Friedberg et al., 2019; Geraghty et al., 2019). Guidelines published in 2021 by NICE recommend that GET should not be used and that CBT should only be used to control symptoms and reduce distress, not to aid recovery. The evidence for other interventions such, as exercise training (Tai et al., 2022), moxibustion, acupuncture (Fang et al., 2022), music (Jacquet et al., 2021), qigong, massage and tuina (Alraek et al., 2011) is still deemed inconclusive.

In light of limited treatment options for CFS, many patients with CFS, who desire autonomy in management and perceive natural resources as safe, resorted to modalities of Traditional Medicine (TM) or Complementary Medicine (CM) as a choice of self-care or under the guidance of healthcare practitioners (Chung et al., 2021). According to the World Health Organization (WHO, 2013), TM is defined as “the sum of the knowledge, skill and practices based on the theories, beliefs and experiences indigenous to different cultures, whether explicable or not, used in the maintenance of health as well as in the prevention, diagnosis, improvement or treatment of physical and mental illness” and CM as “a broad set of healthcare practices that are not part of a country’s own traditional or conventional medicine and are not fully integrated into the dominant healthcare system”. Depending on the cultural background, the structure of the health system and the regulations in the local context, different forms of TM and/or CM practice and preparations may exist. For the purpose of this study, the prime focus rests on TM and CM preparations (T&CMs), which may contain single or multiple herbs, animals, vitamins, minerals, and nutritional compounds in the form of capsules, tablets, decoctions, drink, pills, granules, or mixtures.

More and more research focused on the anti-fatigue effect of T&CMs. Reviews reported natural medicines, such as *Panax ginseng C.A. Mey*. (Jin et al., 2013). and *Lycium barbarum L*. (Jin et al., 2013), for the treatment of fatigue had specific anti-fatigue effects, few toxic side effects, and were rich in pharmacological activity (Luo et al., 2019). Pharmacology studies reported that many herbal ingredients used in T&CMs such as Ginseng root (*P. ginseng C.A. Mey*.) (Reay et al., 2006; Hsiao et al., 2018; Yang et al., 2022) and *Cordyceps militaris* (Song et al., 2015) have specific anti-fatigue effects. Astragali Radix was also found to promote the recovery of fatigue by regulating glucose metabolism, lipid metabolism, and energy metabolism (Li et al., 2014). Other natural ingredients such as *Gynostemma pentaphyllum (Thunb.) Makino* (Lin-Na and Yong-Xiu, 2014), *Portulaca oleracea L*. (Xu and Shan, 2014), and *L. barbarum L*. (Jin et al., 2013) have shown potentials in improving exercise capacity (Li et al., 2014). Rehmannia root [*Rehmanniae Radix*] had a protective effect on the nerves of mouse brain tissue (Zhang et al., 2007) According to the theory of Traditional Chinese Medicine, herbs that nourish “yin” and “blood” such as Angelica root [*Angelica sinensis (Oliv.) Diels*] (Kupfersztain et al., 2003) have also been recommended for chronic fatigue in clinical trials. These pharmacological studies suggest that T&CMs could be a promising treatment strategy for CFS.

The number of randomized controlled trials (RCTs) that investigated the benefits and risks of T&CMs in treating chronic fatigue has been increasing. Multiple attempts have been made to systematically evaluate the evidence yielded from these trials. A Cochrane systematic review in 2009 that aimed to evaluate the effectiveness of traditional Chinese herbal medicine in treating CFS was ceased prematurely as no studies were eligible for inclusion due methodological flaws (Adams et al., 2009). Later on, a meta-analysis in 2013 showed that T&CMs were more effective than Western medicine in treating CFS. However, due to the limited number (a total of 11 studies) and the concerns about the quality of the included studies, the findings were deemed inconclusive (Peng et al., 2013). Another systematic review in 2014 also concluded that the benefits of T&CMs on CFS remained questionable due to the high risks of study bias and very little was reported about the safety (Wang et al., 2014). A more recent meta-analysis in 2022 included larger number of studies (a total of 84 trials) found that Chinese herbal medicine used as an adjuvant or monotherapy for CFS appeared to be effective to improve fatigue (Zhang et al., 2022). However, this meta-analysis only studies concluded in China which may limit the generalizability of the findings.

Considering the evidence about T&CMs for CFS continues to emerge, there is a need to continuously and critically review the evidence available to better inform clinical practice and research design. Based on the experiences of these above-mentioned reviews, future analysis should seek to include RCTs that are of well design, larger sample size, low risks of bias and conducted in multicenter and reported both the efficacy and safety. Therefore, this review aimed to provide a more comprehensive and critical update of the RCTs conducted across the globe that investigated both the efficacy and safety of T&CMs for the treatment of CFS.

## 2 Methods

This study was a systematic literature review conducted and reported in compliance with the updated referred Reporting Items for Systematic reviews and Meta-Analyses (PRISMA) statement (Page et al., 2021a). The Consolidated Standards of Reporting Trials Statement extensions for Chinese Herbal Medicine Formulas (CONSORT-CHM) (Cheng et al., 2017) and the Cochrane Collaboration’s Risk of Bias tool (Higgins et al., 2022) were used in this review to evaluate the reporting quality and the risk of bias of all included trials. The protocol of this systematic review has been registered on PROSPERO (CRD42022362268). [https://www.crd.york.ac.uk/prospero/display_record.php?ID=CRD42022362268].

### 2.1 Inclusion and exclusion criteria

#### 2.1.1 Inclusion criteria

We included randomized, controlled trials which investigated the efficacy and/or safety of T&CMs in chronic fatigue involving participants of any age, gender or ethnic origin, and published in English or Chinese, between 1 January 2013 and 31 December 2022. The comparison might be made against the control groups which might use placebo, pharmacological therapy or no treatment. Pharmacological therapies referred to conventional medicines or other T&CMs such as traditional Chinese medicine patent prescription.

#### 2.1.2 Exclusion criteria

Studies subjected to exclusion were: 1) reviews, meta-analyses, protocols, or observational studies; 2) non-randomized or single-arm clinical trials; 3) pharmacodynamics or pharmacology studies; 4) animal experiments; 5) studies on other non-oral drugs therapies (such as acupuncture, qigong, music, yoga, and mindfulness); 6) other disease studies or studies on fatigue due to other diseases; or 7) studies on non-herbal pharmaceutical ingredients or specific plants or herbs not listed in T&CMs related standards.

### 2.2 Outcome measurements

#### 2.2.1 Primary outcomes

The primary outcome measures considered included scoring of fatigue symptoms using the validated tool Fatigue Scale-14 (FS-14) (Chalder et al., 1993) and changes in clinical symptoms of chronic fatigue syndrome reported using the TCM syndrome score (Luo et al., 2015). FS-14 scale is a common tool generally considered as effective in reflecting the level of fatigue. The FS-14 involved 14 items divided into two categories: physical fatigue (1–8) and mental fatigue (9–14). Higher total score indicated higher severity of fatigue. On the other hand, the TCM syndrome score table was used to evaluate the changes in TCM symptoms in specific types of patients, generally including symptoms such as dreaminess, dizziness and fatigue. Similar to FD-14 score, higher score represented more obvious TCM symptoms in CFS patients.

#### 2.2.2 Secondary outcomes

The secondary outcome measures under considered included the following: other fatigue scales including the fatigue assessment instrument (FAI) (Schwartz et al., 1993), Chalder Fatigue Questionnaire (ChFi-11-item or CFQ-11) (Jackson, 2015), the fatigue severity scale (FSS) (Lerdal, 2020), Multidimensional Fatigue Inventory (MFI-20) (Shahid et al., 2011a), the fatigue assessment scale (FAS) (Hendriks et al., 2018), the checklist individual strengthen (CIS) (Vercoulen et al., 1994), the advanced trail making test (ATMT) (Arnett and Labovitz, 1995), as well as assistive tests such as Visual Analogue Scale (VAS) (Shahid et al., 2011b) and Numerical Rating Scale (NRS) (Gladman et al., 2020).

Other measurement scales might also be considered: 1) Mental health and mental condition reflected by the self-rating anxiety scale (SAS) (Zung, 1971), the self-rating depression scale (SDS) (Zung, 1965), Hamilton Depression Scale (HAMD) (Hamilton, 1960), Hamilton Anxiety Scale (HAMA) (Hamilton, 1959), the symptom checklist 90 (SCL-90) (Derogatis et al., 1973), Beck depression inventory test (BDI) (Beck et al., 1961); 2) Quality of life and health reflected by the 36-item short-form health survey (SF-36) (Ware and Sherbourne, 1992), World Health Organization quality of life scale (WHOQOL-BERF) (Skevington et al., 2004), the EuroQoL 5-dimension 5-level questionnaire (EQ-5D-5L) (Herdman et al., 2011), the Activity of Daily Living Scale (ADL) (Hindmarch et al., 1998); 3) Measurements of other items, such as Pittsburgh sleep quality index (PSQI) (Buysse et al., 1989), insomnia severity index (ISI) (Chalder and Morin, 1993), the Qi blood yin yang deficiency questionnaire (QBYY-Q) (Woo et al., 2008), Qi deficiency Constitution Scoring scale (Bai et al., 2022), the quality of sexual life with SLQ Questionnaire (Woodward et al., 2002), psychosocial stress survey for groups (PSSG) (Jiang, 1998), stress response inventory (SRI) (Kohn and OBrien, 1997).Other measurements reflecting the status of fatigue or related functions such as biochemical tests, immune factors, physiological tests, and nuclear magnetic resonance were also considered.

### 2.3 Search strategy and study selection

#### 2.3.1 Search strategy

Literature was searched systematically according to the updated PRISMA statement (Page et al., 2021b) in seven electronic databases including China National Knowledge Infrastructure (CNKI), Wanfang Data, Pubmed, Scopus, Web of Science (WOS), Embase, the Cochrane Library for RCTs to identify RCTs which evaluated T&CMs in the management or treatment of chronic fatigue syndrome published from the database inception to 31 December 2022. The search strategy focused on three primary terms, “fatigue”, “traditional and complementary medicine preparations (T&CMs)” and “randomized controlled trials (RCTs)”, and was limited to articles published in English or Chinese. As shown in Table 1, to ensure an effective search, Medical Subject Headings (MeSH) terms, common phrases and keywords related to the three primary terms were used to develop a comprehensive search strategy. Using PubMed as an example, the search strategy was as follows:

**TABLE 1:** Search term identifiers.

Primary terms	Entry search terms in English	Entry search terms in Chinese
1. fatigue	Lassitude	慢性疲勞綜合征
	myalgic encephalomyelitis	肌痛性腦脊髓炎
	Chronic Fatigue	肌痛性腦脊髓炎
	Fatigue Syndrome*	慢性疲勞免疫功能紊亂綜合征
	Chronic Fatigue Syndrome*	病毒感染後疲勞綜合征
	Chronic Fatigue Fibromyalgia Syndrome*	疲勞
	Fatigue Disorder*	慢性疲勞
	Postviral Fatigue Syndrome*	CFS
	Chronic Fatigue and Immune Dysfunction Syndrome*	
2. traditional and complementary medicine preparations (T&CMs)	Complementary Therapies[MeSH]	中藥
	“medicine, traditional” [Mesh]	藥物療法
	“Drugs, Chinese Herbal” [Mesh]	植物中藥
	“Medicine, Chinese Traditional” [Mesh]	飲片
	Plant Extracts[MeSH]	中藥配方顆粒
	“Plant Preparations” [Mesh]	草藥
	complementary medicine*	
	alternative medicine*	
	herbal medicine*	
	Chinese medicine*	
	folk medicine*	
	herb*	
	phytotherapy	
	nutraceutical*	
	pharmaceutical plant*	
	plant preparation*	
	medicinal plant*	
	plant medicinal product*	
	folk remed*	
3. randomized controlled trials (RCTs)	clinical	臨床
	trial*	實驗
	clinical study*	試驗
		療效
		觀察

1) AND, retrieves results that include all the search terms. 2)* Including but not limited to.

[“Fatigue”(MeSH Terms) OR “chronic fatigue” (MeSH Terms)] AND [“complementary therapies”(Title/Abstract) OR “medicine, traditional”(Title/Abstract)] AND [“clinical study” (Filter) OR “clinical trial ” (Filter) OR “randomized controlled trial” (Filter)]. Two authors (YL, JS) conducted the literature search independently. A detailed description of each of the search strategies used in each database is provided in Supplementary Material. The search results were discussed between these 2 authors (YL, JS) and confirmed by another author (COLU).

#### 2.3.2 Study selection

The search results, reference lists and citations of included literature were screened independently by two of the authors (YL, JS) to identify possibly eligible studies for inclusion. Duplicates were first removed from the initial search records, followed by screening of title, abstract and full-text by two of the authors (YL, JY) for inclusion.

### 2.4 Data extraction and analysis

All references were classified and archived in Endnote X9. Data was extracted and recorded in a standard table using Excel 2013. Under the supervision and guidance of one author (JS), two other authors (YL, JY) simultaneously extracted the data from five randomly selected studies to assure quality in data extraction. The quality check of data extracted from all the included studies was performed by one author (JS) and confirmed by another author (COLU). After confirming that no data were missing, relevant data from all included studies were independently screened and extracted by two authors (YL, JY) for further analysis, including basic information about studies, methods, interventions, participants, outcomes, and overall findings, as listed in Table 2.

**TABLE 2:** Relevant data from studies included.

Data category	Items
1. Basic information of study	⁃ Information on the first author
	⁃ Publication year and language
	⁃ Recruitment center
2. Methods	⁃ Trial design
	⁃ Date and setting of the trial
	⁃ Criteria for inclusion and exclusion of participants
	⁃ Criteria for diagnosing patients (either according to the theories of TM or the diagnosis criteria of modern medicine)
3. Intervention	⁃ The T&CMs and its dosage used in the experimental group
	⁃ The comparator in the control groups
	⁃ Intervention duration
	⁃ Duration of follow-up were recorded
4. Participants	⁃ The number of participants in the randomization phase
	⁃ The number of participants in the analysis phase
	⁃ Mean age
	⁃ The sex ratio
	⁃ History of chronic fatigue
	⁃ Dropouts
5. Outcomes and overall findings	⁃ Efficacy assessed statistically in terms of primary outcomes, secondary outcomes, and other outcomes
	⁃ Safety assessed either quantitatively or qualitatively in terms of adverse effects reported

### 2.5 Appraisal of reporting quality

Two of the authors (YL, JY) independently assessed each included study using the 25-item version of the CONSORT-CHM 2017 statement (Cheng et al., 2017). Any disagreements in the assessment results were discussed or negotiated among them and later confirmed with the other two authors (JS, COLU). The CONSORT-CHM 2017 statement provided a grading system devised for each criterion that was used to determine the strengths and weaknesses of clinical trials of T&CMs interventions. According to the degree of conformity, the assessment results for each item were determined as non-existent, partially present (for example, if some aspects of the CONSORT project are missing or poorly described), and fully compliant.

### 2.6 Assessment of risks of bias

Referring to the Cochrane guidelines, each study included was critically appraised independently by two of the authors (YL, JY) using the Cochrane Risk of Bias tool. The judgment was based on the definition of the recommendation by the Cochrane Handbook for Systematic Reviews of Interventions, and the assessment result for each item were grouped into one of the following three categories: “low risk of bias”, “unclear risk of bias”, and “high risk of bias”. Further explanation about the risk assessment is shown in Table 3.

**TABLE 3:** Level of risks of bias.

Risk items	Low risk of bias	Unclear risk of bias	High risk of bias
1. Sequence generation	Random methods used were described (e.g., table of random numbers, random block, or computer random number generation)	Information about sequence generation was unknown or difficult to judge	A non-random method in the sequence generation process was indicated
2. Allocation concealment	Participants and the researchers who recruited subjects were not able to predict the allocation (e.g., center assignment, identical containers, opaque sealed envelopes)	No specified information about the allocation methods (e.g., only reported the ratio of different groups)	Other methods unable to be hidden were used to allow participants or the researchers who recruited subjects to predict allocations
3. Blinding of participants and personnel	The study described the trial as blinding and mentioned the methods of blinding to make sure that the allocation of interventions in the study was not known to participants and trial researchers	Blinding and the methods in the study were not mentioned or used blinding methods without any descriptions, or related outcome was not reported in the studies	Blinding was not used or was incomplete, or the blinding might be broken
4. Blinding of outcome assessment	The study described the trial as blinding and mentioned the methods of blinding to make sure that the allocation of interventions in the study was not known to outcome evaluators	The study described the trial as blinding and mentioned the methods of blinding to make sure that the allocation of interventions in the study was not known to outcome evaluators	Blinding was not used or was incomplete, or the blinding might be broken
5. Incomplete outcome data	No missing data in the study or the missing data did not affect the results, or the analysis treated the missing data in an appropriate way	Information was incomplete, and it was difficult to judge whether the data was complete	The number of participants and causes of absence between groups in the studies was unbalanced or other causes affected the effect size of the intervention
6. Selective outcome reporting	A study proposal or all desired outcomes in published studies	Difficult to determine whether there was a risk of selective reporting of results due to incomplete information	Important outcomes were not reported in the studies, or methods of measurement and data analysis for unspecified indicators were reported
7. Other bias	No other sources of bias in the studies	Insufficient information to determine whether to cause an important risk of bias or no good justification or evidence that could lead to bias	Potential biases or some other issues related to particular study designs in the studies

## 3 Results

### 3.1 Search results

**FIGURE 1:**
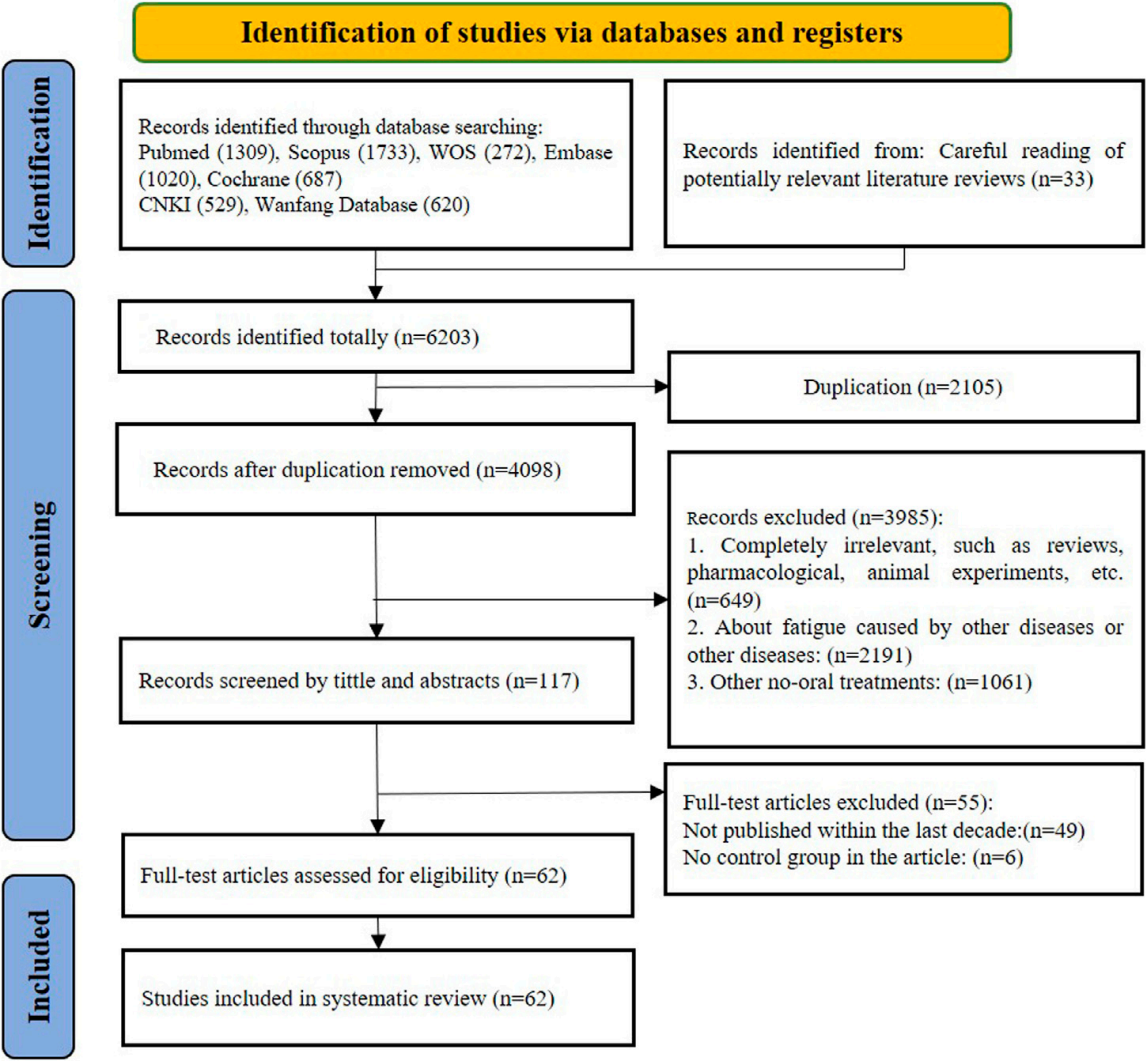
PRISMA flow-chart of study selection.

Based on the PRISMA guidelines, screening process was conducted as shown in Figure 1. Initially, 6,170 records were yielded through searching of the seven electronic databases, and 33 records that met the criteria were found in the citations of other reviews, for a total of 6,203 records. After removing 4,098 duplicates, 3,985 articles were retained for further screening. During the process of screening by title and abstract, 2,191 records were excluded due to multiple reasons: completely irrelevant study or publication type (review or meta-analysis or protocol articles or animal experiments, etc.) (*n* = 649); focus on other diseases or symptoms of fatigue developed from other diseases: (*n* = 2,191); interventions using non-oral treatment other than T&CMs such as music, food, acupuncture, moxibustion or others (*n* = 1,061). Subsequently, the full text of 117 records were further assessed for eligibility of which 55 were excluded. Given the timeliness of the evidence, only trials published between 1 January 2013 and 31 December 2022 were included, resulting in 49 studies being excluded. To avoid a high placebo effect and to get the better validate effectiveness, single-arm RCTs were excluded (*n* = 6). Eventually, 62 trials were eligible for analysis in this review.

### 3.2 Description of studies

Among the 62 studies included in this review, 57 trials were published in Chinese and 5 trials were published in English. The RCTs were conducted in China (*n* = 58) (81-138) and Korea (*n* = 4) (Kim et al., 2013; Joung et al., 2019; Sung et al., 2020; Shin et al., 2021). All trials set a parallel design with double-arm or triple-arm. More details are shown inSupplementary Table S8. All but one trial (Dai et al., 2014) did not report specific information about the place or way of participant recruitment, including hospitals or medical centers. Among them, five studies were multi-center trials (*n* = 5) (Joung et al., 2019; Ma et al., 2019; Shin et al., 2021; Ma et al., 2022; Wu et al., 2022), and the rest were single-center trials (*n* = 56) (81, 84-98, 100-139, 141).

### 3.3 Participants

As shown in Supplementary Table S8; Table 4,195 participants, with 2,221 males and 2,842 females (the number of males and females included in 2 of the studies (Dai et al., 2014; Liu et al., 2021) were not detailed), were eventually allocated into the test groups (*n* = 2,704) and the control or comparison groups (*n* = 2,497). Participants in the included trials ranged in age from 15 to 75 years, with majority between 20 and 65 years. Four trials included adolescents with participants aged 15–44 years (Wang, 2014; Liu A. et al., 2015; Yang and Liang, 2016; Li, 2017). Three trials recruited middle-aged and older participants (45–70 years) (Li and Cao, 2015; Hu, 2019; Guo and Huang, 2022). Most included RCTs had no gender requirement. Two trials recruited female patients only (Zhao et al., 2013; Li and Cao, 2015) and two included male participants only (Sun et al., 2016; Wei et al., 2017).

**TABLE 4:** Eligibility of study participants for chronic fatigue in the included RCTs.

Reference	Diagnostic criteria*				Inclusion criteria and exclusion criteria										
	TCM	Type of patient		WM	Physical examinations		Clinical data and medical history		Indicators			Degree of severity		Exclusion	
Zhou et al. (2022)	Other literature or teaching materials	Heart and Spleen deficiency		N/A	N/A		N/A		N/A			N/A		2,6	
Wu et al. (2022)	N/A	Qi and Yin deficiency		CDC1994	Y		N/A		N/A			N/A		1,2,5,6	
Ma et al. (2022)	Guidelines for clinical research of CM2002	Qi and Yin deficiency		CDC1994	Y		N/A		N/A			N/A		N/A	
Li et al. (2022)	Guidelines for clinical research of CM2002	Qi deficiency		CDC1994	N/A		N/A		N/A			N/A		6	
Huang et al. (2022)	Guidelines for clinical research of CM2002	Qi deficiency		CDC1994	N/A		N/A		8≤HAMD score≤35 and (or) 7≤HAMA score≤28			N/A		1,2,3,6	
Guo and Huang (2022)	Other literature or teaching materials	Qi and Blood deficiency		CDC1994	N/A		Y		N/A			N/A		4,5,6	
Liu et al. (2021)	Guidelines for clinical research of CM2002	Liver depression and Spleen deficiency		CDC1994	Y		N/A		N/A			N/A		2,6	
Chen (2021)	CM clinical diagnosis and treatment terminology 1997	Liver depression and Spleen deficiency		CDC1994	Y		N/A		N/A			N/A		2,6,7	
Shin et al. (2021)	N/A	N/A		CDC1994	N/A		N/A		CIS score>76			N/A		2,4,5,6	
Kan et al., 2021	N/A	N/A		CDC1994	N/A		N/A		N/A			N/A		1,3,5	
Zhang (2020)	N/A	N/A		Other literature or teaching materials	N/A		N/A		N/A			N/A		2	
Sheng (2020)	Guidelines for clinical research of CM2002	Liver depression and Spleen deficiency		CDC1994	N/A		N/A		N/A			N/A		N/A	
Mao (2020)	Other literature or teaching materials	Spleen, Kidney and Yang deficiency		N/A	N/A		N/A		N/A			N/A		5,6	
Li (2020)	Other literature or teaching materials	Spleen and Qi deficiency		N/A	N/A		N/A		N/A			N/A		N/A	
Huang et al. (2020)	Other literature or teaching materials	Qi deficiency		CDC1994	N/A		N/A		N/A			N/A		1,2,6,7	
Dong (2020)	CM clinical Diagnosis and treatment terminology 1997	Spleen deficiency and humid heat		CDC1994	N/A		Y		N/A			N/A		2,4	
Sung et al. (2020)	N/A	N/A		CDC1994	Y		N/A		N/A			N/A		N/A	
Yang et al. (2019)	Other literature or teaching materials	Kidney deficiency		CDC1994	N/A		Y, 6–18 m		N/A			N/A		1,2,6	
Wang (2019)	Other literature or teaching materials	Liver depression and Spleen deficiency		N/A	N/A		N/A		N/A			N/A		N/A	
Shi and Zha (2019)	Guidelines for clinical research of CM2002	Liver depression and Spleen deficiency		CDC1994	N/A		N/A		N/A			N/A		1,2,6	
Ma et al. (2019)	Other literature or teaching materials	Deficiency syndrome		CDC1994	N/A		N/A		N/A			N/A		1,2,6	
Liu et al. (2019a)	CM clinical diagnosis and treatment terminology 1997	Spleen and kidney deficiency		CDC1994	N/A		N/A		N/A			N/A		1,6	
Liu et al. (2019b)	Guidelines for clinical research of CM2002	Liver depression and Spleen deficiency		CDC1994	N/A		N/A		N/A			N/A		N/A	
Liu et al. (2019c)	Guidelines for clinical research of CM2002	Liver depression and Spleen deficiency		CDC1994	N/A		N/A		N/A			N/A		N/A	
Liu et al. (2019d)	N/A	N/A		CDC1994	N/A		N/A		N/A			N/A		1,2,3	
Lin et al. (2019)	Guidelines for clinical research of CM2002	Qi and Blood deficiency		CDC1994	N/A		N/A		N/A			N/A		1,2	
Li et al. (2019)	Guidelines for CFS with TCM 2008	Kidney deficiency		CDC1994	Y		N/A		N/A			N/A		1,24	
Hu (2019)	Other literature or teaching materials	Liver depression and Spleen deficiency		N/A	N/A		N/A		N/A			N/A		N/A	
Ding (2019)	N/A	Heart and Spleen deficiency		CDC1994	N/A		N/A		N/A			N/A		2,5	
Joung et al. (2019)	N/A	N/A		CDC1994	N/A		N/A		N/A			N/A		1	
Wu et al. (2018)	N/A	Heart and Spleen deficiency		CDC1994	N/A		N/A		N/A			N/A		1	
Ou et al. (2018)	Guidelines for clinical research of CM2002	Heart and Spleen deficiency		CDC1994	Y		N/A		blood pressure, or blood glucose were detected			N/A		2	
Liu and Cai (2018)	Other literature or teaching materials	Liver depression and Spleen deficiency		CDC1994	Y		N/A		blood pressure, or blood glucose were detected			N/A		1,2,6	
Li et al. (2018)	Internal Medicine of TCM	N/A		CDC1994	N/A		N/A		N/A			N/A		N/A	
Du (2018)	CM clinical diagnosis and treatment terminology 1997	Kidney and Yin deficiency		CDC1994	N/A		N/A		N/A			N/A		1,2,4,6	
Zheng et al. (2017)	Guidelines for clinical research of CM2002	Liver depression, Spleen and Kidney deficiency		CDC1994	N/A		N/A		N/A			N/A		1,2,3	
Wei et al. (2017)	Guidelines for clinical research of CM2002	Liver and Kidney deficiency		CDC1994	Y		N/A		0.7–3.5 g/L < IgA <0.7 g/L 7.0–16.6 g/L < IgG <7.0 g/L 0.5–2.6 g/L < IgM <0.5 g/L			N/A		N/A	
Wang (2017)	Guidelines for CFS with TCM 2008	Spleen and kidney deficiency		CDC1994	N/A		N/A		N/A			N/A		1,2,3	
Li (2017)	Other literature or teaching materials	Qi deficiency		N/A	N/A		N/A		N/A			N/A		N/A	
Huang et al. (2017)	N/A	N/A		CDC1994	N/A		N/A		N/A			N/A		2,6,7	
Yang and Liang (2016)	Other literature or teaching materials	Yang deficiency		CDC1994	Y		N/A, ≥6 m		Routine laboratory tests			N/A		1,2	
Wu et al. (2016)	Guidelines for clinical research of CM2002	Spleen and kidney deficiency		CDC1994	N/A		N/A		Daosheng Four Diagnostic Instrument			N/A		2	
Sun et al. (2016)	Guidelines for clinical research of CM2002	Liver depression and Kidney deficiency		CDC1994	N/A		N/A		N/A			N/A		1,2	
Huang et al. (2016)	Internal Medicine of TCM	N/A		CDC1994	N/A		N/A		N/A			N/A		1,2,7	
Gao and Pang (2016)	Guidelines for CFS with TCM 2008	Liver depression and Spleen deficiency		CDC1994	Y		N/A		blood pressure, or blood glucose were detected			N/A		1,2,6	
Wang et al. (2015)	Other literature or teaching materials	Qi deficiency		CDC1994	Y		N/A		N/A			N/A		1,2,3,4,6	
Liu et al. (2015a)	CM clinical diagnosis and treatment terminology 1997	Liver depression and Blood deficiency		CDC1994	N/A		N/A		N/A			N/A		1,6	
Liu et al. (2015b)	Internal Medicine of TCM	Spleen and Qi deficiency, Liver depression and stagnation		CDC1994	Y		N/A, ≥6 m		Routine laboratory tests			N/A		1,2	
Li (2015)	N/A	Liver depression and Spleen deficiency		CDC1994	N/A		N/A		N/A			N/A		1	
Li and Cao (2015)	Guidelines for clinical research of CM2002	Spleen and kidney deficiency, Liver depression and stagnation		CDC1994	N/A		N/A		N/A			N/A		1,2	
Guo and Guo (2015)	Internal Medicine of TCM	Liver depression and Spleen deficiency		CDC1994	Y		N/A		No positive physical signs, blood pressure, or blood glucose were detected			N/A		2,5,7	
Gao and Pang (2015)	Guidelines for CFS with TCM 2008	Liver depression and Spleen deficiency		CDC1994	Y		N/A		blood pressure, or blood glucose were detected			N/A		1,2,6	
Wang et al. (2014)	Internal Medicine of TCM	Spleen and Qi deficiency, Liver depression and stagnation		CDC1994	Y		N/A, ≥6 m		Routine laboratory tests			N/A		1,2	
Teng et al. (2014)	Guidelines for clinical research of CM2002	Qi deficiency		CDC1994	N/A		N/A		N/A			N/A		1,2,3	
Niu et al. (2014)	CM clinical diagnosis and treatment terminology 1997	Liver depression and Kidney deficiency		CDC1994	Y		N/A		Routine laboratory tests			N/A		1,2	
Liu et al. (2014)	Internal Medicine of TCM	Spleen and Qi deficiency, Liver depression and stagnation		CDC1994	Y		N/A, ≥6 m		Routine laboratory tests			N/A		1,2	
Dai et al. (2014)	N/A	Deficiency syndrome		CDC1994	Y		N/A, 6–18 m		Routine laboratory tests			severe		N/A	
Zhao et al. (2013)	Guidelines for clinical research of CM2002	Qi and Blood deficiency		CDC1994	Y		N/A		N/A			N/A		7	
Xu et al. (2013a)	N/A	N/A		CDC1994	N/A		N/A		N/A			N/A		N/A	
Xu and Wang (2013)	Internal Medicine of TCM	Liver depression and Spleen deficiency		CDC1994	N/A		N/A		N/A			N/A		2,3,4,6,7	
Sun (2013)	N/A	N/A		CDC1994	N/A		N/A		N/A			N/A		2	
Kim et al. (2013)	N/A	N/A		CDC1994	N/A		N/A		BDI score < 29 and STAI score <70			ICF		1,3,5,6	

^1^Examination to evaluate fatigue caused by other diseases.

^2^Other diseases (chronic diseases such as cardiovascular, rheumatism and immunity, cancer, infection, mental illness) or.

^3^Check items for abnormalities (blood pressure, blood sugar, BMI value).

^4^Past medical history (smoking, alcoholism, drug abuse, recent use of other drugs, or recent participation in other experiments).

^5^Other interventions (use of other drugs Special populations (pregnant and nursing women, mentally unstable or involuntary women, night workers, researchers).

^6^Individual patient circumstances (inability to check, poor compliance, failure to provide outcome measures).

Routine laboratory tests: CBC, urinalysis, erythrocyte sedimentation rate, electrolytes, blood glucose, liver function, renal function, thyroid-stimulating hormone.

Others: other literature or teaching materials.

Fifty-nine trials reported the history of participants with CFS, out of them, twenty-six trials only reported the range of participants’ history (Wang, 2014; Liu Y. et al., 2015; Liu A. et al., 2015; Gao and Pang, 2015; Li and Cao, 2015; Wang et al., 2015; Gao and Pang, 2016; Huang et al., 2016; Huang et al., 2017; Wei et al., 2017; Ou et al., 2018; Wu et al., 2018; Liu et al., 2019b; Liu et al., 2019c; Ding, 2019; Joung et al., 2019; Li et al., 2019; Lin et al., 2019; Ma et al., 2019; Kan et al., 2021; Liu et al., 2021; Shin et al., 2021). Twenty-two trials reported the range of participants’ history with mean history and standard deviation in the test and control groups (Xu D. et al., 2013; Xu Z. et al., 2013; Zhao et al., 2013; Liu et al., 2014; Guo and Guo, 2015; Li, 2015; Wu et al., 2016; Yang and Liang, 2016; Wang, 2017; Du, 2018; Li et al., 2018; Liu and Cai, 2018; Liu Y. et al., 2019; Hu, 2019; Mao, 2020; Sheng, 2020; Chen, 2021; Guo and Huang, 2022; Huang et al., 2022; Zhou et al., 2022). Three trials reported the average history of participants without providing the standard deviation (Su, 2013; Teng et al., 2014; Liu F. et al., 2019). Eight trials reported mean history and standard deviation in the test and control groups without the range of participants’ history (Kim et al., 2013; Niu et al., 2014; Wang, 2019; Dong, 2020; Sung et al., 2020; Li et al., 2022; Ma et al., 2022; Wu et al., 2022). The remaining three trials provided no information about the participants’ CFS condition (Li, 2017; Li, 2020; Zhang, 2020).

A total of 44 participants withdrew from ten trials (Zhao et al., 2013; Huang et al., 2016; Huang et al., 2017; Joung et al., 2019; Huang et al., 2020; Sung et al., 2020; Kan et al., 2021; Li et al., 2022; Ma et al., 2022; Wu et al., 2022), none of which exceeded 10% of the sample size in each trial, participant withdrew before intervention started in two trials, the rest dropped out during treatment, and only one trial reported specific reasons, details as shown in Supplementary Table S8, but did not report the reasons for dropout.

As shown in Table 4, among the included RCTs, the types of participants varied. Their eligibility was determined using different methods and criteria. Most of the participants recruited were diagnosed according the relevant standards of both Traditional Chinese Medicine and Modern Medicine (*n* = 41) (83-88, 91, 94-96, 98-102, 104, 105, 109-115, 118-125, 127-133, 135, 137), participants recruited for the included studies were only diagnosed for CFS according to the diagnosis standards in Modern Medicine (*n* = 15) (Kim et al., 2013; Su, 2013; Dai et al., 2014; Huang et al., 2017; Wu et al., 2018; Ding, 2019; Liu F. et al., 2019; Joung et al., 2019; Sung et al., 2020; Kan et al., 2021; Shin et al., 2021). In the remaining 6 studies, participants were recruited for diagnosis solely based on the relevant standards of Traditional Chinese Medicine (*n* = 6) (Li, 2017; Hu, 2019; Wang, 2019; Li, 2020; Mao, 2020; Zhou et al., 2022). Clear diagnostic details indicating CFS were presented in Table 4, including medical history enquiry, laboratory tests or physical examinations, as well as relevant fatigue scale scores to confirm the severity of CFS.

With Modern Medicine, the common diagnosis criteria reported in the studies was developed by the CDC in 1994 (Fukuda et al., 1994). Other tools from Modern Medicine used as diagnosis criteria included scales such as CIS score higher than 76 points at baseline (Shin et al., 2021), Korean version of the BDI score lower than 29 points and the Korean translation of the State–Trait Anxiety Inventory (STAI) score lower than 70 points (Kim et al., 2013), Hamilton Depression Scale (HAMD) scores on a scale of 8–35 point, and (or) Hamilton Anxiety Scale (HAMA) scores on a scale of 7–28 point (Huang et al., 2022). Laboratory tests (Dai et al., 2014; Liu et al., 2014; Wang, 2014; Liu A. et al., 2015; Yang and Liang, 2016) were used as another major diagnostic method in Modern Medicine, including complete blood count (CBC), urinalysis, erythrocyte sedimentation rate, electrolytes, blood glucose, liver function, renal function, thyroid-stimulating hormone. The diagnostic method applicable to both modern and traditional medical diagnostic was the investigation of past medical history (Dai et al., 2014; Liu et al., 2014; Wang, 2014; Liu A. et al., 2015; Yang and Liang, 2016; Yang et al., 2019; Dong, 2020; Guo and Huang, 2022), including clinical data inquiry. More details were shown in Table 4.

According to the theory of Traditional Chinese Medicine, fatigue in participants of the included studies was diagnosed based on the Guidelines for clinical research of new traditional Chinese medicines (2002) (Zheng, 2002), Clinical research guidelines for the treatment of chronic fatigue syndrome by new Chinese medicine drugs (2008) (Luo et al., 2008), State Administration of Traditional Chinese Medicine. Clinical diagnosis and treatment terminology and syndrome of traditional Chinese medicine (1997) (WHO, 1997), multiple versions of internal Medicine of Traditional Chinese Medicine textbooks (Zhou, 2007), and other literature or teaching materials. Referring to these criteria, participants were classified into the following 19 categories, namely, Liver depression and Spleen deficiency (*n* = 14) (Xu Z. et al., 2013; Gao and Pang, 2015; Guo and Guo, 2015; Li, 2015; Gao and Pang, 2016; Liu and Cai, 2018; Liu et al., 2019b; Liu et al., 2019c; Hu, 2019; Shi and Zha, 2019; Wang, 2019; Sheng, 2020; Chen, 2021; Liu et al., 2021), Qi deficiency (*n* = 6) (Teng et al., 2014; Wang et al., 2015; Li, 2017; Huang et al., 2020; Huang et al., 2022; Li et al., 2022), Heart and Spleen deficiency (*n* = 4) (Ou et al., 2018; Wu et al., 2018; Ding, 2019; Zhou et al., 2022), Qi and Blood deficiency (*n* = 3) (Zhao et al., 2013; Lin et al., 2019; Guo and Huang, 2022), Spleen and kidney deficiency (*n* = 3) (Wu et al., 2016; Wang, 2017; Liu Y. et al., 2019), Spleen and Qi deficiency, Liver depression and stagnation (*n* = 3) (Liu et al., 2014; Wang, 2014; Liu A. et al., 2015), Qi and Yin deficiency (*n* = 2) (Ma et al., 2022; Wu et al., 2022), Kidney deficiency (*n* = 2) (Li et al., 2019; Yang et al., 2019), Deficiency syndrome (*n* = 2) (Dai et al., 2014; Ma et al., 2019), Liver depression and Kidney deficiency (*n* = 2) (Niu et al., 2014; Sun et al., 2016), Spleen, Kidney and Yang deficiency (*n* = 1) (Mao, 2020), Spleen and Qi deficiency (*n* = 1) (Li, 2020), Spleen deficiency and humid heat (*n* = 1) (Dong, 2020), Kidney and Yin deficiency (*n* = 1) (Du, 2018), Liver depression, Spleen and Kidney deficiency (*n* = 1) (Zheng et al., 2017), Liver and Kidney deficiency (*n* = 1) (Wei et al., 2017), Yang deficiency (*n* = 1) (Yang and Liang, 2016), Liver depression and Blood deficiency (*n* = 1) (Liu Y. et al., 2015), Spleen and kidney deficiency, and Liver depression and stagnation(*n* = 1) (Li and Cao, 2015).

### 3.4 Interventions

As shown in Supplementary Table S9, the combination of various ingredients was the main intervention investigated in the 62 included studies. Different forms of T&CM preparations were tested, including decoctions (*n* = 39) (82-86, 88, 90, 92, 93, 95, 97-100, 104, 106-111, 113, 115-119, 121-124, 126, 127, 129, 131, 132, 134, 136, 137), capsules (*n* = 5) (Kim et al., 2013; Sun et al., 2016; Joung et al., 2019; Sung et al., 2020; Zhou et al., 2022), plaster (*n* = 6) (Liu et al., 2014; Wang, 2014; Liu A. et al., 2015; Wei et al., 2017; Du, 2018; Huang et al., 2020), granules (*n* = 5) (Liu et al., 2019b; Liu et al., 2019c; Sheng, 2020; Liu et al., 2021; Shin et al., 2021), pills (*n* = 2) (Liu F. et al., 2019; Yang et al., 2019), tablets (*n* = 2) (Guo and Guo, 2015; Kan et al., 2021), drink (*n* = 1) (Li et al., 2019), mixtures (*n* = 1) (Li et al., 2019) and prepared slices (*n* = 1) (Su, 2013). Dosage forms including capsules, granules, pills, tablets, or mixtures of the T&CM tested group had clear quality standards or control. Most of the remaining T&CM interventions tested were formulated decoctions, and 16 studies mentioned that the basic medication could be adjusted according to the symptoms of individual participants during treatment and follow-up (Dai et al., 2014; Niu et al., 2014; Teng et al., 2014; Li, 2015; Wang et al., 2015; Wang, 2017; Du, 2018; Ou et al., 2018; Wu et al., 2018; Ding, 2019; Hu, 2019; Shi and Zha, 2019; Wang, 2019; Dong, 2020; Mao, 2020; Chen, 2021). Only eight RCTs of the included trials reported follow-up data, and four individual trials had follow-up longer than 6 months. Only one trial calculated recurrence rates without complete or detailed data.

The T&CMs investigated were mostly formulations of multiple herbal ingredients originated from Traditional Chinese Medicine. A total of forty-three formulas, including Buzhong Yiqi Decotion Combine with Xiaochaihu Decotion (*n* = 5), Chaihu Guizhi Decoction (*n* = 4), Wenzhen Yunqi Formula (*n* = 3), Guipi Decoction (*n* = 3), Jianpi Jieyu Xiaopi Paste (*n* = 3), Dispelling Dampness-Replenishing Qi-Nourishing Yin Step Therapy (*n* = 2), Modified Huangqi Jianzhong Decoction (*n* = 2). The following formulations were only used once in the included trials, namely, Xinshen’an Capsule, Guashen Decoction, Fali Decoction, Xiaoyao powder, Yishen Tiaodu Method, Yangwei Jianpi Plaster, Qingshu Yiqi Decotion, Zuogui Pills, Modified Xiaoyao San, Modified Erxian Decoction, Jianpi Yishen Decoction, Modified Lingzhi Pills, Self-made fatigue Decoction, Dalishen Tea, Bupiwei Xieyinhuo Shengyang Decoction, Yiqi Yangxue Bupi Hegan Decoction, Self-made Yishen Buxue Ointment, Shugan Jianpi Yishen Decoction, Long Gao, Bupi Yishen Decoction, Xiaopi - Yin, Shugan Yiyang Capsule, Wendan Decoction Combined with Sini powder, Yiqi Jianpi Bushen Decoction, Shugan Yangxue Method, Invigorating spleen warming kidney and smoothing liver Decoction, Danzhi Xiaoyao tablet, Shugan Jianpi Method, Buzhong Jiepi Decoction, Bushen Shugan Decoction, Buyi Pishen Decoction, Fufangteng Mixture, Modified Naoxinkang, Chaihu Combine with Longgu Muli Decoction, Panax quinquefolius L.prepared Slices. In the remaining 5 RCTs, Sipjeondaebo-tang (SJDBT), Korean red ginseng (KRG), Myelophil, Cistanche and Ginkgo extracts, and Extract of P. ginseng did not show any superior anti-fatigue effects when compared with placebo.

At least 148 ingredients were used in the included T&CM preparations for the treatment of CFS. Top 20 most common materials found in the T&CM preparations included *Astragali Radix (黃芪), Glycyrrhizae Radix et Rhizoma (甘草)*, *Atractylodis Macrocephalae Rhizoma (白朮)*, *Bupleuri Radix (柴胡), Codonopsis pilosula (Franch.) Nannf.* o*r C. pilosula Nannf.var.modesta (Nannf.L.T.Shen or Codonopsis tangshen Oliv) (党参*), *Paeoniae Radix Alba (白芍)*, *Angelicae Sinensis Radix (當歸)*, *Poria (茯苓)*, *Citri Reticulatae Pericarpium (陳皮), Curcumae Radix (鬱金), Ziziphus jujuba Mill.(大枣), Pinellia ternata (Thunb.) Breit. (半夏), Rehmanniae Radix (地黄), Zingiber officinale Rosc. (生姜)*, *Chuanxiong Rhizoma (川芎), Dioscoreae Rhizoma (山药), Ginseng Radix et Rhizoma (人參), Epimedii Folium (淫羊藿), Scutellaria baicalensis Georgi (黄芩)*and *Cinnamomum cassia Presl (桂枝).*

As shown in Supplementary Table S8, among the included trials, the duration of the intervention ranged from 10 days to 4 months in the test and control groups, namely, 10 days (*n* = 1) (Xu D. et al., 2013), 2 weeks or 14 days (*n* = 2) (Wang, 2019; Zhang, 2020), 3 weeks (*n* = 1) (Shi and Zha, 2019), 4 weeks or 28 days or 1 month (*n* = 19) (Kim et al., 2013; Su, 2013; Gao and Pang, 2015; Li, 2015; Li and Cao, 2015; Gao and Pang, 2016; Wang, 2017; Liu and Cai, 2018; Liu et al., 2019b; Liu et al., 2019c; Liu F. et al., 2019; Li et al., 2019; Lin et al., 2019; Huang et al., 2020; Li, 2020; Sheng, 2020; Liu et al., 2021; Guo and Huang, 2022; Li et al., 2022), 6 weeks (*n* = 5) (Liu Y. et al., 2015; Wu et al., 2016; Du, 2018; Liu Y. et al., 2019; Sung et al., 2020), 8 weeks (*n* = 6) (Niu et al., 2014; Teng et al., 2014; Zheng et al., 2017; Mao, 2020; Shin et al., 2021; Zhou et al., 2022), 9 weeks or 60 days or 2 months (*n* = 7) (Yang and Liang, 2016; Li, 2017; Ma et al., 2019; Chen, 2021; Kan et al., 2021; Ma et al., 2022; Wu et al., 2022), 12 weeks or 90 days or 3 months (*n* = 12) (Zhao et al., 2013; Liu et al., 2014; Wang, 2014; Liu A. et al., 2015; Huang et al., 2016; Huang et al., 2017; Wei et al., 2017; Ou et al., 2018; Ding, 2019; Joung et al., 2019; Dong, 2020; Huang et al., 2022) and 4 months (*n* = 1) (Yang et al., 2019).The duration of the intervention described in three trials varied, namely, 15–30 days (*n* = 1) (Li et al., 2018), 4–8 weeks (*n* = 1) (Wang et al., 2015) and 30–40 days (*n* = 1) (Dai et al., 2014).

### 3.5 Control and comparison

Two trials of the 62 studies had two control groups, therefore, the total number of control interventions was sixty-four, included placebo (*n* = 8) (Kim et al., 2013; Liu et al., 2019b; Liu et al., 2019c; Joung et al., 2019; Sung et al., 2020; Kan et al., 2021; Liu et al., 2021; Shin et al., 2021), pharmacological medicine (*n* = 54) or no treatment (*n* = 2) (Kim et al., 2013; Kan et al., 2021). For the 54 trials which tested T&CM against pharmacological medicine, conventional western medicine (*n* = 35) (Xu D. et al., 2013; Xu Z. et al., 2013; Su, 2013; Zhao et al., 2013; Niu et al., 2014; Teng et al., 2014; Liu Y. et al., 2015; Gao and Pang, 2015; Guo and Guo, 2015; Li, 2015; Li and Cao, 2015; Gao and Pang, 2016; Sun et al., 2016; Wu et al., 2016; Wang, 2017; Zheng et al., 2017; Li et al., 2018; Liu and Cai, 2018; Ou et al., 2018; Wu et al., 2018; Liu Y. et al., 2019; Liu F. et al., 2019; Hu, 2019; Li et al., 2019; Ma et al., 2019; Shi and Zha, 2019; Yang et al., 2019; Li, 2020; Mao, 2020; Guo and Huang, 2022; Huang et al., 2022) or traditional Chinese medicine patent prescription (*n* = 16) (Wang et al., 2015; Huang et al., 2016; Yang and Liang, 2016; Huang et al., 2017; Li, 2017; Wei et al., 2017; Lin et al., 2019; Wang, 2019; Dong, 2020; Huang et al., 2020; Sheng, 2020; Zhang, 2020; Li et al., 2022; Ma et al., 2022; Wu et al., 2022; Zhou et al., 2022), or traditional Chinese medicine combined with the conventional western medicine therapy (*n* = 3) (Liu et al., 2014; Wang, 2014; Liu A. et al., 2015) were used as comparators. Detailed information is provided in Supplementary Table S9.

### 3.6 Efficacy outcomes reported

As shown in Table 5, a total of 29 different scales were reported in sixty-two trials to measure the outcomes of CFS interventions. As the primary outcomes, FS-14 (*n* = 27) (Xu D. et al., 2013; Xu Z. et al., 2013; Liu et al., 2014; Niu et al., 2014; Liu Y. et al., 2015; Liu A. et al., 2015; Guo and Guo, 2015; Wang et al., 2015; Sun et al., 2016; Yang and Liang, 2016; Huang et al., 2017; Zheng et al., 2017; Du, 2018; Liu et al., 2019b; Liu et al., 2019c; Liu F. et al., 2019; Ma et al., 2019; Shi and Zha, 2019; Mao, 2020; Sheng, 2020; Liu et al., 2021; Guo and Huang, 2022; Huang et al., 2022; Li et al., 2022; Ma et al., 2022; Wu et al., 2022; Zhou et al., 2022) and TCM syndrome scores (*n* = 24) (Gao and Pang, 2015; Wang et al., 2015; Gao and Pang, 2016; Sun et al., 2016; Wu et al., 2016; Wei et al., 2017; Zheng et al., 2017; Du, 2018; Liu and Cai, 2018; Ou et al., 2018; Wu et al., 2018; Liu et al., 2019c; Ding, 2019; Hu, 2019; Li et al., 2019; Shi and Zha, 2019; Dong, 2020; Huang et al., 2020; Liu et al., 2021; Huang et al., 2022; Li et al., 2022; Ma et al., 2022; Wu et al., 2022; Zhou et al., 2022) were used in similar number of studies, and twelve of these trials used both scales simultaneously (Wang et al., 2015; Sun et al., 2016; Zheng et al., 2017; Du, 2018; Liu et al., 2019c; Shi and Zha, 2019; Liu et al., 2021; Huang et al., 2022; Li et al., 2022; Ma et al., 2022; Wu et al., 2022; Zhou et al., 2022). Secondary outcomes included the following categories of scales and fatigue-related measures. Different types of scales used were ranked from highest to lowest frequency of use.

**TABLE 5:** The results of the outcome measurements of the RCTs included in the review.

Reference	Primary outcome	Second outcome	Other test	Overall efficacy rate	Mild adverse effects		Serious adverse reactions
Zhou et al. (2022)	FS-14*, TCM syndrome score*	A:FAS*	"BI:NK cell*	T: 87.84% C: 73.97%	ADR1		None
Wu et al. (2022)	FS-14*, TCM syndrome score*	C:SF-36*	IF:T cell*(CD3^+^, CD4 + and CD8+T),IgA*,IgM*,IgE*"	T: 98.0% C: 84.0%	ADR2		None
Ma et al. (2022)	FS-14*, TCM syndrome score*	None	None	T: 98% C: 84%	ADR2		None
Li et al. (2022)	FS-14*, TCM syndrome score*	None	IF:IgG*,IgA*,IgM*	T: 97.5% C: 82.9%	N/A		N/A
Huang et al. (2022)	FS-14*, TCM syndrome score*	B:HAMD*,HAMA*	IF:IL-6*,TNF-α*, and IgG*,IgA*,IgM*	TCM syndrome T: 90.00% C: 66.67% FS-14 T: 86.67% C: 60.00%	N/A		N/A
Guo and Huang (2022)	FS-14*	B:SDS*,SAS*	NMR (1H-MRS)*	T: 93.33% C: 63.33%	ADR3		None
Liu et al. (2021)	FS-14*, TCM syndrome score*	D:VAS*	None	T: 88.89% C: 36.11%	None		None
Chen (2021)	None	E:PSQI*	None	T: 87.9% C: 66.7%	None		None
Shin et al. (2021)	None	B:SDS*,SAS*	None	T: 35.4% C:54.2% *p* = 0.101	ADR4		None
Kan et al., 2021	None	A:FAI*,CFQ-11*	None	T(H):81.4% T(L):72.4% C: 65.50%	ADR5		none
Zhang (2020)	None	A:FSS*,CFQ-11*,CIS	BI:Blood ammonia, glucose, free fatty acid, creatine kinase, C-reactive protein, lactic acid, estradiol (only for females), and testosterone (only for males); ALT,AST,GGT, and BUN	None	N/A		N/A
Sheng (2020)	FS-14**	C:EQ-5D 5L	None	T: 91.67% C: 65.50%	N/A		N/A
Mao (2020)	FS-14*	D:VAS*	IF:TNF-α*,IL-6*,IFN-γ*, and IL-1β*	T: 96.67% C: 79.31%	N/A		N/A
Li (2020)	None	E:PSQI,QBYY-Q*	None	T: 94.44% C: 77.78%	N/A		N/A
Huang et al. (2020)	TCM syndrome score*	A:CFQ-11***	None	T: 92.5% C: 87.5%	None		None
Dong (2020)	TCM syndrome score*	C:WHOQOL-BERF**	None	T: 92.50% C: 72.50%	N/A		N/A
Sung et al. (2020)	None	E:SLQ**	None	None	None		None
Yang et al. (2019)	None	A:MFI-20*	BI:Antioxidants: d-ROMs, TBARS, BAP, and SOD; Cortisol concentration: salivary cortisol	T: 90.00% C: 60.00%	N/A		N/A
Wang (2019)	None	C:SF-36*	None	T: 97.5% C: 82.5%	N/A		N/A
Shi and Zha (2019)	FS-14*, TCM syndrome score*	None	None	T: 89.74% C: 68.29%	None		None
Ma et al. (2019)	FS-14**	E:PSQI*	None	T: 90.0% C: 77.5%	N/A		N/A
Liu et al. (2019a)	None	C:SF-36*	None	T: 93.33% C: 73.33%	N/A		N/A
Liu et al. (2019b)	FS-14**	A:FAI*	BI:SOD*, LPO*, GSH-Px*, MDA*, CAT*	T: 88.89% C: 36.11%	None		None
Liu et al. (2019c)	FS-14**, TCM syndrome score**	C:WHOQOL-BERF*	IF:TNF-α*,IL-1β*,IL-6*, and IFN-γ*	T: 88.89% C: 36.11%	None		None
Liu et al. (2019d)	FS-14*	E:ADL*	IF:IgG*,IgA*,IgM*	T: 90.0% C: 80.0%	N/A		N/A
Lin et al. (2019)	None	A:FSS,CFQ-11	None	T: 94.0% C: 48.0%	N/A		N/A
Li et al. (2019)	TCM syndrome score*	B:BDI	None	T: 88.57% C: 60.00%	None		None
Hu (2019)	TCM syndrome score*	C:EQ-5D 5L	None	T: 96.9% C: 72.7%	N/A		N/A
Ding (2019)	TCM syndrome score*	D:VAS	None	T: 93.33% C: 70.00%	N/A		N/A
Joung et al. (2019)	None	E:ISI,SRI	None	None	ADR6		None
Wu et al. (2018)	TCM syndrome score*	B:SAS*	BI: Oxidative Stress, and Cytokines	T: 95.35% C: 72.09%	N/A		N/A
Ou et al. (2018)	TCM syndrome score*	A:MFI-20*	IF:IgA*,IgG*, and IgM*	T: 85.0% C: 67.5%	None		None
Liu and Cai (2018)	TCM syndrome score*	None	None	T: 92.68% C: 75.61%	ADR7		None
Li et al. (2018)	None	None	None	T: 96.67% C: 76.67%	ADR8		None
Du (2018)	FS-14*, TCM syndrome score*	None	None	T: 90.74% C: 75.93%	N/A		N/A
Zheng et al. (2017)	FS-14**, TCM syndrome score**	None	IF:IgA,IgG, and IgM	T: 91.1% C: 71.1%	N/A		N/A
Wei et al. (2017)	TCM syndrome score*	None	IF:IL-6**,TNF-α**, and INF-γ**	T: 77.78% (clinical) C: 76.6% (clinical) T: 80.00% (scale) C: 56.67% (scale)	N/A		N/A
Wang (2017)	None	None	IF:IgA, IgG and IgM	T: 84.29% C: 61.53%	N/A		None
Li (2017)	None	A:ATMT*	None	T: 96.67% C: 76.67%	N/A		N/A
Huang et al. (2017)	FS-14*	D:VAS*	None	None	ADR9		none
Yang and Liang (2016)	FS-14*	None	BI*	T: 90.0% C: 76.7%	N/A		N/A
Wu et al. (2016)	TCM syndrome score*	None	None	T: 95.24% C: 78.95%	ADR10		None
Sun et al. (2016)	FS-14*, TCM syndrome score*	None	None	T: 90.00% C: 70.00%	ADR11		None
Huang et al. (2016)	None	A:FSS,CFQ-11(NRS)	None	None	N/A		N/A
Gao and Pang (2016)	TCM syndrome score*	C:SF-36	NMR(1H-MRS)*	T: 91.43% C: 74.29%	N/A		N/A
Wang et al. (2015)	FS-14*, TCM syndrome score***	D:VAS	None	T: 86.67% C: 68.33%	N/A		N/A
Liu et al. (2015a)	FS-14*	None	None	None	N/A		N/A
Liu et al. (2015b)	FS-14**	None	None	None	N/A		N/A
Li (2015)	None	A:FAI*	None	T: 97.06% C: 85.29%	N/A		N/A
Li and Cao (2015)	None	None	None	T: 91.9% C: 56.8%	N/A		N/A
Guo and Guo (2015)	FS-14*	None	None	T: 93.33% C: 75.56%	ADR12		None
Gao and Pang (2015)	TCM syndrome score*	None	BI	T: 91.43% C: 74.29%	N/A		N/A
Wang et al. (2014)	FS-14*, TCM syndrome score*	None	None	T: 97.14% C: 77.14%	N/A		N/A
Teng et al. (2014)	None	None	None	T: 93.3% C: 66.7%	N/A		N/A
Niu et al. (2014)	FS-14*	None	None	None	N/A		N/A
Liu et al. (2014)	FS-14**	B:HAMD*,HAMA*	None	T: 92.5% C: 70.0%	N/A		N/A
Dai et al. (2014)	None	E:Qi deficiency Constitution Scoring scale*	None	T: 85.00% C: 45%	N/A		N/A
Zhao et al. (2013)	None	A:MFI-20*	None	T: 89.1% C: 61.8%	N/A		N/A
Xu et al. (2013b)	FS-14**	C:SF-36*	Relapse rate	T: 95.00% C: 78.57%	N/A		N/A
Xu and Wang (2013)	FS-14*	E:PSSG	None	T: 92.86% C: 73.81%	ADR13		None
Sun (2013)	None	B:SDS*,SAS*	None	T: 93.33% C: 66.67%	N/A		N/A
Kim et al. (2013)	None	B:HAMD*,HAMA*	None	None	ADR14		None

*p < 0.05, **p < 0.01, ***p < 0.001. BI: biochemical indicators, IF: immunization indicators, NMR: nuclear magnetic resonance, TNF-α: tumor necrosis factor -α, IL-1β: interleukin-1β, IL-6: interleukin-6, IFN-γ: interferon-γ, ALT: alanine aminotransferase, AST: aspartate transaminase, GGT: gamma-glutamyl transferase, BUN: blood urea nitrogen.

ADR1: The incidence of total adverse reactions was T: 10 (13.51%), C: 12 (16.44%), and the symptoms were nausea and vomiting, dizziness, headache, abdominal pain and anemia.; ADR2: Loose stools and increased frequency of stools; ADR3: Nausea, abdominal pain, diarrhea, rash; ADR4: Sixteen participants reported adverse events (T: 9; C: 7) Includes hot flashes, headache, heartburn, migraine, dermatitis, nausea, lower abdominal pain, dizziness, heavy stomach, loss of appetite, and muscle pain; ADR5: seven (3.68%) mild adverse events were reported, including upper respiratory tract infection (*n* = 3), diarrhea (*n* = 1), elevated BUN concentration at Day 30 (*n* = 2), and elevated ALT and AST (*n* = 1). However, none of these adverse events were related to the study products; ADR6: Diarrhea, knee pain, common cold, migraine, neck pain, diarrhea, pulpitis, diarrhea, cough; ADR7: T: 2 cases of nausea, 1 case of diarrhea, 1 case of dyspepsia, the overall incidence of 9.76% C: 1 case of nausea, 1 case of vomiting, the total incidence of 4.88%; ADR8: Thirteen participants reported adverse events (T:3; C: 10) Includes dizziness, dry mouth, drowsiness; ADR10: T: 2 cases had nausea, dry mouth, and vomiting after taking the drug. C: 3 patients had dry mouth, skin itching, rash, and loss of appetite; ADR11: T: 5 cases had thirst, dry mouth, nausea, and lack of diet, and C: 13 cases had thirst, dry mouth, nausea, and lack of diet; ADR12: T: 7 cases had nausea, dizziness, dry mouth, headache; C: 15 patients developed dizziness, nausea, dry mouth, drowsiness; ADR13: T: 6 patients with dry mouth, nausea, C: 18 patients with obvious dry mouth, malignant; ADR14: T: 6 patients with dry mouth, nausea, C: 18 patients with obvious dry mouth, malignant.

Other fatigue scales: CFQ-11 (*n* = 5), FAI (*n* = 5), FSS (*n* = 4), MFI-20 (*n* = 3), FAS (*n* = 1), CIS (*n* = 1), ATMT (*n* = 1), as well as assists in measuring fatigue, VAS (*n* = 7), NRS (*n* = 2).; Measurements reflecting the status of fatigue or related functions: inflammatory factor (*n* = 9), biochemical indicators (*n* = 8), nuclear magnetic resonance (*n* = 2), and relapse rate (*n* = 1).; Other measurement scales: Mental health and mental condition scales: SAS (*n* = 6), SDS (*n* = 5), HAMD (*n* = 3), HAMA (*n* = 3), SCL-90 (n = 2), BDI (*n* = 1); Quality of life and health scales: SF-36 (*n* = 4), WHOQOL-BERF (*n* = 3), EQ-5D 5L (*n* = 2); Measurements of other items: PSQI (*n* = 4), QBYY-Q (*n* = 1), SLQ Questionnaire (*n* = 1), ISI (*n* = 1), Qi deficiency Constitution Scoring scale (*n* = 1), PSSG (*n* = 1), SRI (*n* = 1), ADL (*n* = 1).

Nineteen trials measured quantifiable physiological and biochemical indicators. Only nine reported statistically significant differences in pre- and post-intervention measurements between the test and control groups (Zheng et al., 2017; Du, 2018; Wu et al., 2018; Liu Y. et al., 2019; Liu et al., 2019c; Sheng, 2020; Li et al., 2022; Ma et al., 2022; Zhou et al., 2022), with the levels of serum immunoglobulin A (IgA), immunoglobulin M (IgM) and immunoglobulin E (IgE) reporting the most discrepancies.

### 3.7 T&CMs efficacy

A total of 58 out of 62 included trials reported overall effectiveness in their results, of which 53 trials calculated overall response rates. In the test groups, the overall efficacy rates were in the range between 77.7% and 98.0%, of which 37 trials reported more than 90% of overall efficacy rate (Xu D. et al., 2013; Xu Z. et al., 2013; Su, 2013; Liu et al., 2014; Teng et al., 2014; Wang, 2014; Gao and Pang, 2015; Guo and Guo, 2015; Li, 2015; Li and Cao, 2015; Gao and Pang, 2016; Sun et al., 2016; Wu et al., 2016; Yang and Liang, 2016; Li, 2017; Zheng et al., 2017; Du, 2018; Li et al., 2018; Liu and Cai, 2018; Wu et al., 2018; Liu Y. et al., 2019; Ding, 2019; Liu F. et al., 2019; Hu, 2019; Lin et al., 2019; Ma et al., 2019; Wang, 2019; Yang et al., 2019; Dong, 2020; Huang et al., 2020; Li, 2020; Mao, 2020; Sheng, 2020; Guo and Huang, 2022; Li et al., 2022; Ma et al., 2022; Wu et al., 2022) and only one trial with less than 50% overall efficacy rate (Shin et al., 2021). In the control groups, the overall efficacy rates ranged from 36.11% to 87.5%. The remaining six did not calculate the total efficacy rate. Instead, the difference in scale scores between the test group and the control group before and after treatment was used to assess the improvement in fatigue after interventions and showed statistical significance (*p* < 0.05) (Niu et al., 2014; Liu Y. et al., 2015; Liu A. et al., 2015; Huang et al., 2016; Huang et al., 2017; Zhang, 2020). Three trials did not report a statistically significant difference in fatigue improvement in the treatment group compared to the control group (Kim et al., 2013; Joung et al., 2019; Sung et al., 2020). Nevertheless, the trial of Sung et al. (2020) analyzing the subgroup with initial fatigue VAS below 80 mm and age greater than 50 years showed that the reduction in fatigue VAS was significantly greater if KRG was used instead of placebo (Sung et al., 2020). An analysis of a subpopulation of severe symptoms (NRS value ≥ 63), in the trial of Joung et al. (2019) showed a statistically significant improvement in fatigue symptoms in the Myelophil group compared with placebo (*p* < 0.05 for NRS, FSS, and SF-36) (Joung et al., 2019). In the trial of Kim et al. (2013), the NRS score of the ginseng dosing groups did not improve for physical fatigue symptoms, but significantly improved for mental fatigue symptoms. The ginseng groups also showed reduced ROS and MDA levels compared to placebo (Kim et al., 2013).

### 3.8 T&CMs safety

Only twenty-four out of 62 included studies monitored and reported any suspected adverse effects associated with the use of T&CM. Among them, 10 RCTs found no adverse reactions associated with the T&CM interventions tested (Kim et al., 2013; Huang et al., 2017; Ou et al., 2018; Liu et al., 2019b; Liu et al., 2019c; Li et al., 2019; Shi and Zha, 2019; Huang et al., 2020; Sung et al., 2020; Chen, 2021; Liu et al., 2021) while the remaining 14 trials had reported suspected adverse reactions. Among these 14 RCTs which reported adverse reactions, 4 RCTs only indicated the occurrence of adverse reactions and the types of adverse reactions (Joung et al., 2019; Guo and Huang, 2022; Ma et al., 2022; Wu et al., 2022), whereas the other 10 RCTs provided further information about the number of participants affected and whether they came from the test or control group (Xu Z. et al., 2013; Kim et al., 2013; Guo and Guo, 2015; Sun et al., 2016; Wu et al., 2016; Li et al., 2018; Liu and Cai, 2018; Kan et al., 2021; Shin et al., 2021; Zhou et al., 2022). All adverse reactions reported were non-serious and could mainly be divided into the following three categories: (Son, 2019): gastrointestinal discomfort (such as decreased appetite, diarrhea, stomach discomfort or mild pain, nausea, vomiting, diarrhea); (vant Leven et al., 2010); mild allergic reactions (dermatitis, systemic rash, itching); (Brurberg et al., 2014); and other minor complaints of cough, headache, blurred vision, dizziness, toothache, bleeding gums, flu-like symptoms, and other symptoms. Three trials reported discontinuation or disappearance of adverse effects after symptomatic treatment without affecting the results of the study.

### 3.9 Quality assessment

The results of the quality assessment are summarized in Table 6. Only two of the 62 RCTs fully reported the 25 items required for the CONSORT-CHM. The two most common reasons for non-compliance were poor “Methods” (such as sample size, allocation, implementation, or blinding) and “Results” (such as participant flow, numbers analyzed, ancillary analysis or harms).

**TABLE 6:** Evaluation of included trial studies using the CONSORT-CHM statement.

Reference	Title	Abstract	Keywords	Background	Objectives	Trial design	Participants	Interventions	Outcomes	Sample size	Randomization	Allocation	Implementation	Blinding
Zhou et al. (2022)	△	△	△	△	△	△	○	△	○	×	○	×	×	×
Wu et al. (2022)	○	△	△	△	△	△	○	△	○	×	△	×	△	○
Ma et al. (2022)	△	△	△	△	△	○	○	△	○	×	○	○	○	○
Li et al. (2022)	○	△	△	△	△	△	○	△	○	×	△	×	×	×
Huang et al. (2022)	△	△	△	△	△	○	○	△	○	○	○	×	×	×
Guo and Huang (2022)	△	△	△	△	○	○	○	△	○	×	○	×	×	×
Liu et al. (2021)	○	△	△	△	○	△	△	△	○	×	○	○	○	○
Chen (2021)	○	△	△	△	△	△	○	△	△	×	○	×	×	×
Shin et al. (2021)	○	△	○	○	○	○	△	○	○	○	○	△	○	○
Kan et al., 2021	○	○	△	○	○	△	○	○	○	○	○	○	○	△
Zhang (2020)	△	△	△	△	△	△	△	△	△	×	△	×	×	×
Sheng (2020)	○	△	○	△	△	○	○	△	△	×	△	△	×	△
Mao (2020)	○	△	△	△	△	△	△	△	○	×	△	×	×	×
Li (2020)	△	△	△	△	△	△	△	△	△	×	○	×	×	×
Huang et al. (2020)	○	△	△	△	△	△	△	△	△	×	○	×	×	×
Dong (2020)	○	△	△	△	△	△	○	△	○	×	○	×	×	×
Sung et al. (2020)	△	○	△	○	○	○	○	○	○	○	○	○	○	○
Yang (2019)	○	△	△	△	△	△	○	△	○	×	△	×	×	△
Wang (2019)	△	△	△	△	△	△	△	△	△	×	△	×	×	△
Shi (2019)	○	△	△	△	△	△	△	△	△	×	○	×	×	×
Ma et al. (2019)	○	△	△	△	△	△	○	△	○	×	△	×	×	×
Liu et al. (2019a)	△	△	△	△	○	○	○	△	○	×	△	×	×	△
Liu et al. (2019b)	○	△	△	△	△	△	△	△	△	×	○	○	○	○
Liu et al. (2019c)	○	△	△	△	○	△	△	△	○	×	○	○	○	○
Liu et al. (2019d)	○	△	○	△	△	△	○	△	△	×	△	×	×	×
Lin et al. (2019)	○	△	△	△	△	△	△	△	△	×	○	×	×	×
Li et al. (2019)	△	△	△	△	△	△	○	△	△	×	○	×	×	×
Hu (2019)	○	△	△	△	△	○	△	△	△	×	△	×	×	×
Ding (2019)	○	△	△	△	△	△	△	△	△	×	△	×	×	×
Joung et al. (2019)	○	○	○	○	○	○	○	○	○	○	○	△	○	○
Wu et al. (2018)	○	△	○	△	△	○	△	△	△	×	△	×	×	×
Ou et al. (2018)	○	△	○	△	△	○	○	△	△	×	○	×	×	×
Liu and Cai (2018)	△	△	△	△	△	△	○	△	△	×	○	×	×	×
Li et al. (2018)	△	△	△	△	△	○	△	△	△	×	△	×	×	×
Du (2018)	○	△	△	△	△	△	△	△	△	×	○	×	×	×
Zheng et al. (2017)	○	△	△	△	△	△	△	△	△	×	△	×	×	×
Wei et al. (2017)	○	△	△	△	△	△	○	△	△	×	△	×	×	△
Wang (2017)	○	○	○	△	△	△	○	△	○	×	△	×	×	×
Li (2017)	○	△	△	△	△	△	×	△	△	×	○	×	×	×
Huang et al. (2017)	○	△	○	△	△	△	○	△	○	×	△	×	×	×
Yang and Liang (2016)	○	△	△	△	×	△	○	△	△	×	△	×	×	×
Wu et al. (2016)	○	△	△	△	△	○	○	△	○	×	○	×	×	×
Sun et al. (2016)	○	△	△	△	△	△	○	△	○	×	○	×	×	△
Huang et al. (2016)	○	△	△	△	△	○	○	△	○	○	○	×	×	×
Gao and Pang (2016)	△	△	△	△	△	△	△	△	△	×	○	×	×	×
Wang et al. (2015)	○	△	△	△	△	○	○	△	△	×	○	×	×	△
Liu et al. (2015a)	△	△	△	△	○	△	○	△	△	×	○	×	×	×
Liu et al. (2015b)	△	△	△	△	△	△	△	△	△	×	△	×	×	×
Li (2015)	○	△	△	△	△	△	○	△	△	×	△	×	×	×
Li and Cao (2015)	○	△	△	△	△	△	○	△	△	×	△	×	×	×
Guo and Guo (2015)	△	△	△	△	△	△	○	△	△	×	○	×	×	×
Gao and Pang (2015)	△	△	△	△	○	○	○	△	△	×	○	×	×	×
Wang et al. (2014)	△	△	△	△	△	△	○	△	△	×	△	×	×	×
Teng et al. (2014)	△	△	△	△	△	△	△	△	△	×	△	×	×	×
Niu et al. (2014)	○	△	△	×	△	△	○	△	△	×	△	×	×	×
Liu et al. (2014)	△	△	△	△	△	△	○	△	△	×	△	×	×	×
Dai et al. (2014)	△	△	△	△	△	△	△	△	△	×	×	×	×	×
Zhao et al. (2013)	△	△	△	△	△	○	○	△	△	×	○	×	×	×
Xu et al. (2013)	△	△	△	△	△	△	△	△	△	×	△	×	×	×
Xu and Wang (2013)	△	△	△	△	△	○	○	△	△	×	○	×	×	×
Sun (2013)	△	△	△	△	△	△	△	△	△	×	△	×	×	×
Kim et al. (2013)	△	○	×	○	○	○	○	○	○	○	○	△	△	△

Statistical methods	Participant flow	Recruitment	Baseline data	Numbers analysis	Outcomes	Ancillary analysis	Harms	Limitations	Generalizability	Interpretation	Registration	Protocol	Funding
○	×	○	△	×	△	×	○	△	△	○	△	△	○
○	×	○	△	○	△	×	○	×	○	○	×	△	○
○	×	○	△	○	△	×	○	×	○	○	△	△	○
○	×	△	○	△	△	×	△	×	○	○	△	△	○
○	×	○	○	△	△	×	×	○	△	○	△	△	○
○	×	○	○	×	△	×	○	×	○	○	×	×	×
○	×	○	△	×	△	×	○	×	△	△	△	△	○
○	×	○	△	×	△	×	○	×	△	○	×	△	×
○	○	○	△	○	○	×	○	○	○	○	○	○	○
○	○	○	○	○	○	×	○	○	○	○	○	○	×
○	×	○	△	×	△	×	×	×	△	△	×	△	×
○	×	○	△	×	△	×	×	×	△	△	△	△	×
○	×	○	△	×	△	×	×	×	△	△	×	△	×
○	×	○	×	×	△	×	×	×	△	△	×	×	×
○	×	○	△	△	△	×	○	△	△	△	△	△	○
○	×	○	△	×	△	×	×	×	△	○	△	×	×
○	○	○	○	○	△	○	△	○	○	○	○	○	○
○	×	○	△	×	△	×	×	×	△	△	△	△	×
○	×	○	△	×	△	×	×	×	△	△	×	×	×
○	×	○	△	△	△	×	○	×	△	○	△	△	×
○	×	○	△	×	△	×	×	×	△	△	×	×	○
○	×	○	△	×	△	×	×	△	○	○	○	△	○
○	×	△	△	△	△	×	○	×	△	○	×	△	○
○	×	△	△	△	△	×	○	△	△	○	×	△	○
○	×	○	△	×	△	×	×	×	△	○	×	×	×
○	×	○	△	×	△	×	×	×	△	△	×	△	○
○	×	○	△	×	△	×	○	×	△	○	×	△	○
○	×	○	△	×	△	×	×	×	△	△	×	△	×
○	×	○	×	×	△	×	×	×	△	△	×	△	×
○	○	○	○	○	○	○	○	○	○	○	○	○	○
○	×	○	△	×	△	×	×	×	△	△	×	×	○
○	×	○	△	×	△	×	○	×	△	○	×	△	○
○	×	○	△	×	△	×	○	×	△	△	△	△	×
○	×	○	×	×	△	×	○	×	△	△	×	△	×
○	×	○	×	×	△	×	×	×	△	○	△	△	×
○	×	○	×	×	△	×	×	○	△	○	×	△	×
○	×	○	△	×	△	×	×	×	×	△	×	△	○
○	×	○	○	○	△	×	△	×	○	△	△	△	×
○	×	○	△	×	△	×	×	×	△	△	×	×	×
○	×	△	△	△	△	×	○	×	△	○	×	△	○
○	×	×	△	△	△	×	×	×	△	△	×	△	×
○	×	○	△	×	△	×	○	×	△	△	×	△	○
○	×	○	△	×	△	×	○	△	△	△	×	△	○
○	×	○	△	△	△	×	×	×	△	○	×	△	○
○	×	○	△	×	△	×	×	×	△	○	×	△	○
○	×	○	○	×	△	×	×	×	△	△	×	△	○
○	×	×	△	×	△	×	×	×	○	○	×	△	○
○	×	△	△	×	△	×	×	×	△	△	×	△	○
○	×	○	△	×	△	×	×	×	△	△	×	×	×
○	×	○	△	×	△	×	×	×	△	△	×	×	×
○	×	○	△	×	△	×	○	×	○	○	×	△	○
○	×	○	△	×	△	×	×	×	○	○	×	△	○
○	×	○	△	×	△	×	×	×	○	○	×	△	○
○	×	○	×	×	△	×	×	×	△	△	×	×	×
×	×	×	△	×	△	×	×	×	△	△	×	△	○
○	×	○	×	△	△	×	×	×	○	○	×	△	○
×	×	×	×	×	△	×	×	△	△	△	×	×	×
○	×	○	×	△	△	×	×	×	△	△	×	△	×
○	×	○	△	×	△	×	×	×	△	△	×	×	×
○	×	○	△	×	△	×	○	×	△	△	×	△	×
○	×	○	×	×	△	×	○	×	△	△	×	×	×
○	○	○	○	○	○	△	○	×	○	○	○	○	○

○, Fully satisfied; △, Partially satisfied; ×, Not satisfied.

Only one trial did not report randomization (Dai et al., 2014), and of the additional 61 included trials, 22 trials mentioned randomization without specifying the method (Xu D. et al., 2013; Su, 2013; Dai et al., 2014; Liu et al., 2014; Niu et al., 2014; Teng et al., 2014; Wang, 2014; Liu A. et al., 2015; Li, 2015; Li and Cao, 2015; Yang and Liang, 2016; Huang et al., 2017; Li et al., 2018; Wu et al., 2018; Liu Y. et al., 2019; Liu F. et al., 2019; Ma et al., 2019; Wang, 2019; Yang et al., 2019; Sheng, 2020; Zhang, 2020; Li et al., 2022; Wu et al., 2022), and the remaining 39 trials reported the detailed randomization methods at low risk of bias, the most commonly used include, namely, random number table (*n* = 22) (Xu Z. et al., 2013; Zhao et al., 2013; Liu Y. et al., 2015; Gao and Pang, 2015; Guo and Guo, 2015; Wang et al., 2015; Sun et al., 2016; Wu et al., 2016; Li, 2017; Zheng et al., 2017; Li et al., 2018; Liu and Cai, 2018; Ou et al., 2018; Liu et al., 2019b; Li et al., 2019; Shi and Zha, 2019; Dong, 2020; Huang et al., 2020; Li, 2020; Guo and Huang, 2022; Huang et al., 2022; Zhou et al., 2022), and the block randomization (*n* = 10) (Kim et al., 2013; Gao and Pang, 2016; Liu et al., 2019c; Joung et al., 2019; Sung et al., 2020; Chen, 2021; Kan et al., 2021; Liu et al., 2021; Shin et al., 2021; Ma et al., 2022). Blinding was mentioned in seventeen studies, but only eight trials adequately described how this was conducted in detail (Liu et al., 2019b; Liu et al., 2019c; Joung et al., 2019; Sung et al., 2020; Liu et al., 2021; Shin et al., 2021; Ma et al., 2022; Wu et al., 2022). The remaining nine trials mentioned blinding but could not ensure the reliability of blinding (Kim et al., 2013; Wang et al., 2015; Sun et al., 2016; Wei et al., 2017; Liu Y. et al., 2019; Wang, 2019; Yang et al., 2019; Sheng, 2020; Kan et al., 2021). Forty-five trials mentioned no blinding procedures at all (81, 84-86, 88, 90, 92-95, 98, 99, 103-113, 115-119, 121, 122, 124-138). Only 6 studies provided information on allocation (Liu et al., 2019b; Liu et al., 2019c; Sung et al., 2020; Kan et al., 2021; Liu et al., 2021; Ma et al., 2022).

### 3.10 Risk of bias

**FIGURE 2:**
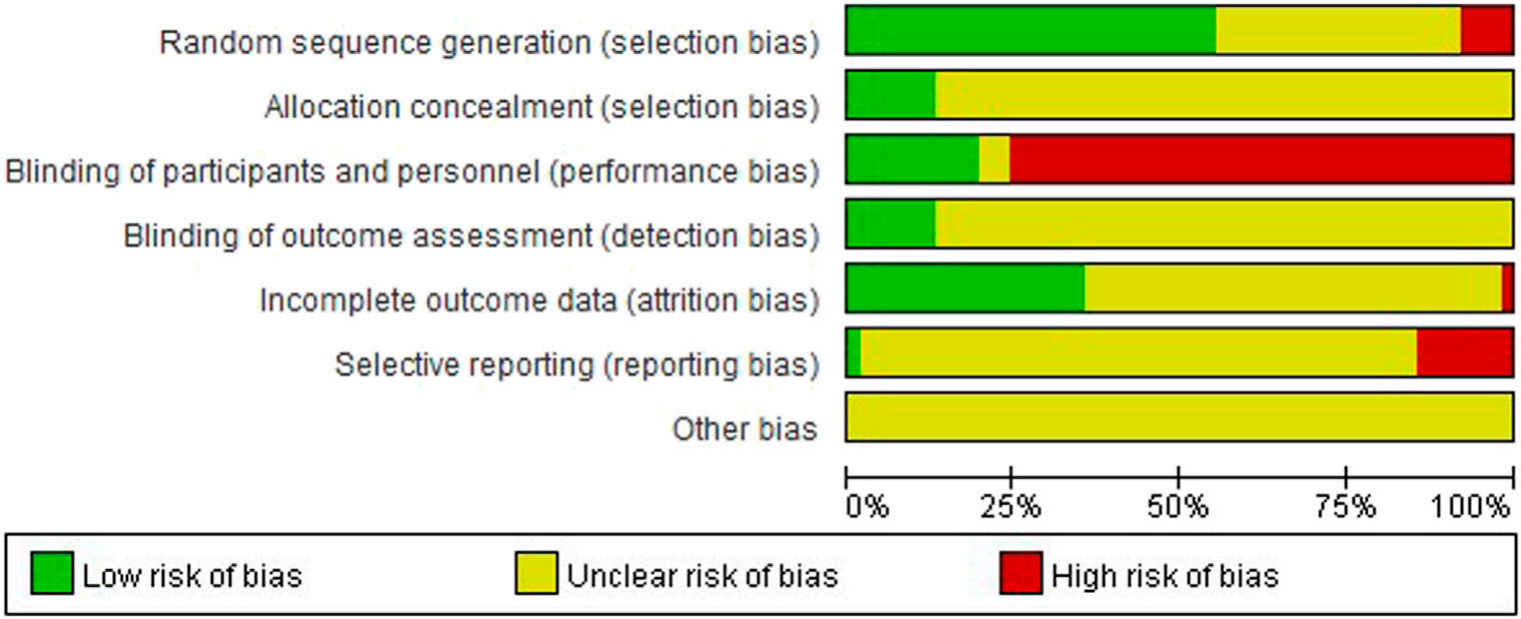
Risk of bias graph: the judgements of the review authors about each risk of bias item presented as percentages across all included studies.

**FIGURE 3:**
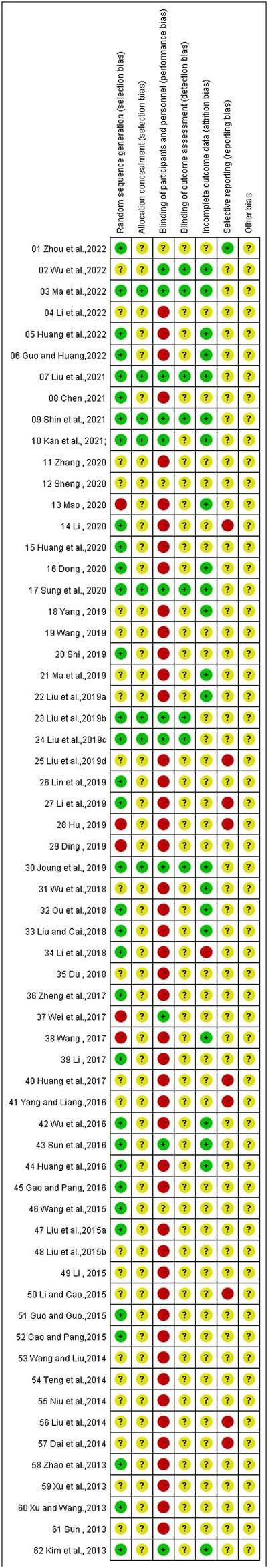
Risk of bias summary: the judgements of the review authors about each risk of bias item presented for each included trial.

The overall risk of bias assessment results of all included trials is shown in Figure 2, and items of bias assessment for each included trial is shown in Figure 3. Most of the included studies provided incomplete information on study design and methodology. All of the included trials were found to be having unclear or high risk of bias in at least one domain. No trial was assessed as at low risk of bias in all domains. A detailed assessment of the seven domains at risk of bias was explained in the following: (Son, 2019): Randomization - only 34 trials were assessed as low risk of bias as all of them adequately reported the use of random sequence generation; (vant Leven et al., 2010); Allocation - only 8 trials reported detailed information on allocation concealment methods, which were considered sufficient to be assessed as low risk of bias, while 54 trials were assessed as unclear risk of bias for not providing specific method of allocation concealment or not describing them at all; (Brurberg et al., 2014); Blinding of participants and personnel - due to incomplete or easily interrupted blinding, 43 trials with blinding information were assessed as having high risk of bias. Three trials were assessed as having unclear risk of bias because no specific method of blinding of participants and personnel was provided; (Carruthers et al., 2011a); Blinding of outcome assessment -only 8 trials were assessed as low risk of bias as they reported detailed information on blinding of fact by outcome assessors, and blinding was not easily compromised; (Vercoulen et al., 1994); Incomplete outcome data - 38 trials reported little or no reporting of this information and were therefore assessed as trials at unclear risk of bias; (Matura et al., 2018); Selective reporting - 9 trials were assessed as having a high risk of reporting bias due to statistical errors or omissions in the results, such as incomplete or unclear interpretation of scale items; and (Jason et al., 1999) Other potential sources of bias - all 62 trials were assessed as unclear risk because of incomplete information reporting, such as failure to report sample size calculations or adverse effects, or lack sufficient justification or evidence to judge bias.

## 4 Discussion

This review systematically analyzed 62 RCTs that investigated the effectiveness and safety of T&CMs for CFS. T&CMs were found to be statistically more effective as an intervention group when compared with control groups in improving symptoms of CFS including physical and mental fatigue, TCM-diagnosed symptoms such as dizziness, tinnitus, or other psychological conditions such as depression or anxiety. Only mild or reversible adverse effects such as mild gastrointestinal discomfort and mild allergic reactions had been reported. Overall, the findings of this review suggested that T&CMs were effective in relieving CFS and relatively safe to use, which reaffirmed and supplemented previous findings (Kupfersztain et al., 2003; Zhang et al., 2007; Cho et al., 2009) with the inclusion of RCTs not limited to any form of T&CMs (e.g., traditional Chinese medicines) (Peng et al., 2013; Wang et al., 2014; Zhang et al., 2022), nor any particular study country (e.g., China) (Peng et al., 2013; Wang et al., 2014; Zhang et al., 2022). However, due to methodological and quality heterogeneity of the included RCTs, the positive findings of the current review should be interpreted with cautions. A number of important implications have been derived from this review, which warrant further consideration in the following.

### 4.1 The role of T&CMs in CFS

Among the 43 formulas investigated in the studies included in this review, the main intervention groups which showed significant benefits were Buzhong Yiqi Decoction combined with Xiao Chaihu Decoction (補中益氣湯合小柴胡湯) and Chaihu Guizhi Decoction (柴胡桂枝湯), which were significantly improved fatigue and the quality of life of participants. Regarding the treatment of fatigue with Buzhong Yiqi Decoction and Xiao Chaihu Decoction, the earliest trace could be traced back to the ancient Chinese medical book “Pujifang” in the Ming Dynasty (Chen et al., 2010). According to the Chinese traditional medical theory, most of the ingredients in the decoction were deficiency supplements, which were consistent with the systemic symptoms and signs of CFS which as liver depression and spleen deficiency, liver qi stasis, spleen deficiency, qi deficiency, blood stasis, liver and kidney yin deficiency, spleen and kidney yang deficiency (Luo et al., 2008).

In some included studies of the review, the T&CMs used were mainly formulations of multiple herbal ingredients originated from Traditional Chinese Medicine, with a wide variety and complex composition, including 148 ingredients. Moreover, in 16 studies, minor adjustments to herbal ingredients based on individual participants' fatigue symptoms (e.g., insomnia and depression) during the study period had been reported. It was called “Suizheng Jiajian” (隨證加減) or “Bianzheng Lunzhi” (辨證論治) with the theory of Chinese medicine (Chen et al., 2010). Compared to the fixed T&CMs preparations, information about the quality standards and the quality control of the T&CMs under investigation was often insufficient or ambiguous, if not lacking, which inevitably raised reasonable doubts about the safety of the T&CMs used by the participants. Reports of future RCTs should provide supporting adequate information that demonstrate the standardization of the T&CMs, such as composition, quality control, detailed dosing regimens and manufacturing processes.

Fatigue resistance can possibly be achieved mainly through reducing oxidant stress, regulating carbohydrate metabolism, delaying the accumulation of metabolites, promoting mitochondrial function, neuroprotection, anti-apoptosis, or regulating neurotransmitter disorder in the central nervous system. Most of herbal ingredients involved in this review included astragalus (*Astragali Radix, 黃芪*), angelica (*Angelicae Sinensis Radix,當歸*), paeony (*Paeoniae Radix Alba,白芍*), ginseng (*Ginseng Radix et Rhizoma,人參*), yam (*Dioscoreae Rhizom*,*山藥*), licorice (*Glycyrrhizae Radix et Rhizom,甘草*), all of which reportedly related to dopamine, hypothalamic-pituitary-thyroid axis disorders, compensatory effects generated by negative feedback inhibition, and activated immune-inflammatory pathways. For instance, increasing evidence has emerged in recent years that indicated the multiple immunomodulatory activities of *Astragali Radix* and *Ginseng Radix* resulting in therapeutic effects against fatigue in preclinical and clinical studies (Chen et al., 2010). Pharmacological studies also found that *Astragali Radix* could promote the recovery of fatigue by regulating glucose metabolism, lipid metabolism, and energy metabolism (Li et al., 2014). *Ginseng Radix* might inhibit oxidative stress and improve mitochondrial function in skeletal muscles (Bao et al., 2016). *Bupleuri Radix* had the functions of analgesic, antibacterial, antiviral, anti-inflammatory, anti-oxidation, and anti-depression. All of these mechanisms may have contributed to the positive impact on chronic fatigue (Jiang et al., 2020). Also, *Paeonia Lactiflora* was found to inhibit 5-HT synthesis and tryptophan hydroxylase expression and thus reduce fatigue during exercise and the resting state (Hong et al., 2003).

### 4.2 Methodological heterogeneity in RCTs

Methodological heterogeneity exists across all trials leading to questionable quality of the RCTs included in this study. Uncertainty about the risk of bias, inconsistent criteria for participant diagnosis and inclusion in RCTs, and inconsistent measures of T&CMs efficacy had made it impractical to perform meta-analysis for a comprehensive evaluation of the current evidence. For improvement in future studies, the following areas warrant careful consideration.

The use of the CONSORT-CHM statement was relatively consistent in only a few trials. The reporting quality of the rest of the studies was generally considered having unclear or high risk of bias. Most studies did not meet the recommended requirements, especially in the missing sample size calculation method, flow chart and other information, incomplete personnel allocation and blinding concealment information. Indeed, sample size calculations should be adequately performed and fully reported to ensure and demonstrate methodological quality. Otherwise, statistical bias, reduced trial quality, a lack of statistical capacity to correctly estimate treatment effects, and overestimation of the risk of intervention benefit were inevitable.

The evaluation results of the Cochrane risk-of-bias tool were also concerning in light of the multiple risks of bias associated with blinding. Although all trials reported that study protocols were designed according to recommended criteria, only eight RCTs reported comprehensive details of the blinding process, including participants (and personnel) and outcome assessment. Blinding is important to minimize bias and maximize the validity of study results (He et al., 2011). However, in other studies, researchers seemed to have failed to make improvements in study design and implementation, and the issue of blinding procedures for RCTs to test T&CM was repeatedly reported, as described in this study (Zhang et al., 2022).

According to the data analysis in this review, it was found that different inclusion and screening criteria of the participants also contributed to heterogeneity across the included studies. Over the past few decades, several countries and organizations developed new or improved diagnostic criteria (Jason et al., 2015). Importantly, most of these standard sets were primarily used for research purposes (e.g., epidemiology, pathophysiology, or therapeutic trials) rather than routine clinical practice (Jason et al., 2003; Sandler and Lloyd, 2020; National Institute for Health and Care Excellence, 2021). The most commonly used standard of Modern Medicine in this review were also drafted by an international expert group convened by the Centers for Disease Control and Prevention in 1994 (CDC) (Fukuda et al., 1994), which had been criticized for being overly accommodating to patients with milder disease (Reeves et al., 2003). Other guidelines and standards of traditional Chinese medicines were only used in China. However, these criteria had not been fully validated in extensive population studies.

In previous studies, the age, gender, and duration of CFS were representative factors affecting participants' function and symptoms (Chang et al., 2012; Stevelink et al., 2022). Women had about twice the risk of developing CFS than men (Faro et al., 2016), Moreover, patients who were older and with long-term CFS were more likely to experience reduced levels of vitality associated with physical fatigue, while short-term CFS patients were presented with worse levels of mental health (Kidd et al., 2016). Most of the included RCTs recruited participants in China, and only four RCTs were conducted in South Korea. These might impact on the generality of evidence and applicability of interventions to other populations.

### 4.3 Future research

A more comprehensive search strategy, stricter screening criteria should be set, ensuring the quality of the included studies, and focusing on other symptoms or causes to construct new diagnostic screening or criteria, and further evaluate the validity of the criteria to better build understanding of CFS disease. There is an urgent need to develop objective diagnostic tools for CFS. Insights into priority areas for improvement in conducting RCTs in T&CMs, such as standardizing the consensus of T&CMs in the diagnosis and treatment of CFS to establish unified comprehensive identification and efficacy measurement standards. It is expected that future RCTs could be improved by increasing awareness and adoption of the CONSORT-CHM guidelines. The CONSORT-CHM guidelines can be used to guide the design of the RCTs and as an assessment tool of the reporting quality. It is also important to conduct multicenter, large-sample RCT of CFS to eliminate influence caused by age and sex and to ensure the validity of results. Conducting clinical studies of multicomponent therapies to enrich the T&CMs studies of CFS and at the same time verify whether it has a comparative advantage over single component therapy in terms of safety and reliability may also be relevant to the practical needs in the clinical setting.

### 4.4 Limitation

This review has a number of limitations. Firstly, this review only searched for RCTs within last 10 years, and did not further compare the results of different time periods. Therefore, it was not possible to assess the development of evidence about T&CMs for CFS over time. Secondly, due to the heterogeneity of results, it was not practical to conduct any meta-analysis to allow for more definitive conclusions. We also did not perform subgroup analyses to give more definitive answers, a shortcoming that should be addressed in our subsequent studies. Thirdly, the CONSORT-CHM extension was used in this review as an evaluation framework aiming to ensure the completeness and transparency in assessing the reporting quality of the RCTs included for analysis. However, few RCTs included in this review adopted the guideline. The overall reporting quality was found sub-optimal which might not necessarily reflect of the overall RCT design. Fourthly, the validated FS-14 and TCM syndrome score were commonly used to measure primary outcomes in the included studies. However, the subjectivity and ceiling effect of the tool itself further limit the reliability of the research results. The strength of the results of this review may be diminished due to these limitations, but this review remains relevant due to the growing popularity of studies of T&CMs interventions for CFS and the lack of conclusive evidence.

## 5 Conclusion

Based on this review, some T&CMs are effective in managing CFS, especially in improving physical and mental fatigue, and without major safety concerns. However, no conclusive recommendations can be made about T&CMs due to methodological heterogeneity and questionable quality of RCTs included in this review. For the development of evidence about T&CMs for CFS, the design of future RCTs should be improved by using large sample sizes, clearly indicating the inclusion criteria, as well as adopting a more focused approach when selecting T&CMs and measures that allow objective investigation of the long-term effectiveness and safety of T&CMs.

## Data Availability

The original contributions presented in the study are included in the article/Supplementary Material, further inquiries can be directed to the corresponding author.
