# Supplementary material for: Is there a role for traditional and complementary medicines in managing chronic fatigue? a systematic review of randomized controlled trials
Source: Front Pharmacol. 2023 Oct 24;14:1266803. doi: 10.3389/fphar.2023.1266803 (PMC10628447; doi:10.3389/fphar.2023.1266803)
Supplement: Supplementary file 1 [file DataSheet1.PDF]

Supplementary Material

Is there a role for traditional and complementary medicines in managing chronic fatigue? a systematic literature review of randomized controlled trials

Yuxiao Li [1, †], Jingya Yang[1, †], Chi Ian Chau[1], Junnan Shi[1], Xianwen Chen[1], Hao Hu[1,2], Carolina Oi Lam Ung [1,2*]

*** Correspondence:** Carolina Oi Lam Ung: carolinaung@um.edu.mo

1 Supplementary Tables

Supplementary Table. 1 Pubmed-Search history

Number	Query	Sort By	Filters	Search Details	Results
**1**	fatigue[MeSH Terms]	Most Recent	/	"fatigue"[MeSH Terms]	36417
**2**	fatigue[Title/Abstract]	Most Recent	/	"fatigue"[Title/Abstract]	119144
**3**	Lassitude[Title/Abstract]	Most Recent	/	"Lassitude"[Title/Abstract]	458
**4**	((fatigue[MeSH Terms]) OR (Lassitude[Title/Abstract])) OR (fatigue[Title/Abstract])	Most Recent	/	"fatigue"[MeSH Terms] OR "Lassitude"[Title/Abstract] OR "fatigue"[Title/Abstract]	129124
**5**	fatigue syndromes	Most Recent	/	("fatiguability"[All Fields] OR "fatiguable"[All Fields] OR "fatigue"[MeSH Terms] OR "fatigue"[All Fields] OR "fatigued"[All Fields] OR "fatigues"[All Fields] OR "fatiguing"[All Fields] OR "fatigueability"[All Fields]) AND ("syndrom"[All Fields] OR "syndromal"[All Fields] OR "syndromally"[All Fields] OR "syndrome"[MeSH Terms] OR "syndrome"[All Fields] OR "syndromes"[All Fields] OR "syndrome s"[All Fields] OR "syndromic"[All Fields] OR "syndroms"[All Fields])	20019
**6**	chronic fatigue[MeSH Terms]	Most Recent	/	("chronic"[All Fields] OR "chronical"[All Fields] OR "chronically"[All Fields] OR "chronicities"[All Fields] OR "chronicity"[All Fields] OR "chronicization"[All Fields] OR "chronics"[All Fields]) AND "fatigue"[MeSH Terms]	4949
**7**	chronic fatigue[Title/Abstract]	Most Recent	/	"chronic fatigue"[Title/Abstract]	8113

**8**	(Fatigue Syndrome*[MeSH Terms]) OR (Chronic Fatigue Syndrome*[MeSH Terms]) OR (Chronic Fatigue Fibromyalgia Syndrome*[MeSH Terms]) OR (Fatigue Disorder*[MeSH Terms]) OR (Postviral Fatigue Syndrome*[MeSH Terms]) OR (Chronic Fatigue and Immune Dysfunction Syndrome*[MeSH Terms])	Most Recent	/	"fatigue syndrome*"[MeSH Terms] OR (("chronic"[All Fields] OR "chronical"[All Fields] OR "chronically"[All Fields] OR "chronicities"[All Fields] OR "chronicity"[All Fields] OR "chronicization"[All Fields] OR "chronics"[All Fields]) AND "fatigue syndrome*"[MeSH Terms]) OR ((("chronic"[All Fields] OR "chronical"[All Fields] OR "chronically"[All Fields] OR "chronicities"[All Fields] OR "chronicity"[All Fields] OR "chronicization"[All Fields] OR "chronics"[All Fields]) AND ("fatiguability"[All Fields] OR "fatiguable"[All Fields] OR "Fatigue"[MeSH Terms] OR "Fatigue"[All Fields] OR "fatigued"[All Fields] OR "fatigues"[All Fields] OR "fatiguing"[All Fields] OR "fatigueability"[All Fields]) AND ("fibromyalgia"[MeSH Terms] OR "fibromyalgia"[All Fields] OR "fibromyalgias"[All Fields])) AND "syndrome*"[MeSH Terms]) OR (("fatiguability"[All Fields] OR "fatiguable"[All Fields] OR "Fatigue"[MeSH Terms] OR "Fatigue"[All Fields] OR "fatigued"[All Fields] OR "fatigues"[All Fields] OR "fatiguing"[All Fields] OR "fatigueability"[All Fields]) AND "disorder*"[MeSH Terms]) OR ("Postviral"[All Fields] AND "fatigue syndrome*"[MeSH Terms]) OR ((("chronic"[All Fields] OR "chronical"[All Fields] OR "chronically"[All Fields] OR "chronicities"[All Fields] OR "chronicity"[All Fields] OR "chronicization"[All Fields] OR "chronics"[All Fields]) AND ("fatiguability"[All Fields] OR "fatiguable"[All Fields] OR "Fatigue"[MeSH Terms] OR "Fatigue"[All Fields] OR "fatigued"[All Fields] OR "fatigues"[All Fields] OR "fatiguing"[All Fields] OR "fatigueability"[All Fields]) AND ("immune system diseases"[MeSH Terms] OR ("immune"[All Fields] AND "system"[All Fields] AND "diseases"[All Fields]) OR "immune system diseases"[All Fields] OR ("immune"[All Fields] AND "dysfunction"[All Fields]) OR "immune dysfunction"[All Fields])) AND "syndrome*"[MeSH Terms])	7041
**9**	(Fatigue Syndrome*[Title/Abstract]) OR (Chronic Fatigue Syndrome*[Title/Abstract]) OR (Chronic Fatigue Fibromyalgia Syndrome*[Title/Abstract]) OR (Fatigue Disorder*[Title/Abstract]) OR (Postviral Fatigue Syndrome*[Title/Abstract]) OR (Chronic Fatigue and Immune Dysfunction Syndrome*[Title/Abstract])	Most Recent	/	"fatigue syndrome*"[Title/Abstract] OR "chronic fatigue syndrome*"[Title/Abstract] OR "chronic fatigue fibromyalgia syndrome*"[Title/Abstract] OR "fatigue disorder*"[Title/Abstract] OR "postviral fatigue syndrome*"[Title/Abstract] OR "chronic fatigue and immune dysfunction syndrome*"[Title/Abstract]	6864

**10**	#4 OR #5 OR #6 OR #7 OR #8 OR #9	Most Recent	/	"Fatigue"[MeSH Terms] OR "Lassitude"[Title/Abstract] OR "Fatigue"[Title/Abstract] OR (("Chronic"[All Fields] OR "chronical"[All Fields] OR "chronically"[All Fields] OR "chronicities"[All Fields] OR "chronicity"[All Fields] OR "chronicization"[All Fields] OR "chronics"[All Fields]) AND "Fatigue"[MeSH Terms]) OR "chronic fatigue"[Title/Abstract] OR ("fatigue syndrome*"[MeSH Terms] OR (("Chronic"[All Fields] OR "chronical"[All Fields] OR "chronically"[All Fields] OR "chronicities"[All Fields] OR "chronicity"[All Fields] OR "chronicization"[All Fields] OR "chronics"[All Fields]) AND "fatigue syndrome*"[MeSH Terms]) OR ((("Chronic"[All Fields] OR "chronical"[All Fields] OR "chronically"[All Fields] OR "chronicities"[All Fields] OR "chronicity"[All Fields] OR "chronicization"[All Fields] OR "chronics"[All Fields]) AND ("fatiguability"[All Fields] OR "fatiguable"[All Fields] OR "Fatigue"[MeSH Terms] OR "Fatigue"[All Fields] OR "fatigued"[All Fields] OR "fatigues"[All Fields] OR "fatiguing"[All Fields] OR "fatigueability"[All Fields]) AND ("Fibromyalgia"[MeSH Terms] OR "Fibromyalgia"[All Fields] OR "fibromyalgias"[All Fields])) AND "syndrome*"[MeSH Terms]) OR (("fatiguability"[All Fields] OR "fatiguable"[All Fields] OR "Fatigue"[MeSH Terms] OR "Fatigue"[All Fields] OR "fatigued"[All Fields] OR "fatigues"[All Fields] OR "fatiguing"[All Fields] OR "fatigueability"[All Fields]) AND "disorder*"[MeSH Terms]) OR ("Postviral"[All Fields] AND "fatigue syndrome*"[MeSH Terms]) OR ((("Chronic"[All Fields] OR "chronical"[All Fields] OR "chronically"[All Fields] OR "chronicities"[All Fields] OR "chronicity"[All Fields] OR "chronicization"[All Fields] OR "chronics"[All Fields]) AND ("fatiguability"[All Fields] OR "fatiguable"[All Fields] OR "Fatigue"[MeSH Terms] OR "Fatigue"[All Fields] OR "fatigued"[All Fields] OR "fatigues"[All Fields] OR "fatiguing"[All Fields] OR "fatigueability"[All Fields]) AND ("immune system diseases"[MeSH Terms] OR ("Immune"[All Fields] AND "system"[All Fields] AND "diseases"[All Fields]) OR "immune system diseases"[All Fields] OR ("Immune"[All Fields] AND "Dysfunction"[All Fields]) OR "immune dysfunction"[All Fields])) AND "syndrome*"[MeSH Terms])) OR ("fatigue syndrome*"[Title/Abstract] OR "chronic fatigue syndrome*"[Title/Abstract] OR "chronic fatigue fibromyalgia syndrome*"[Title/Abstract] OR "fatigue disorder*"[Title/Abstract] OR "postviral fatigue syndrome*"[Title/Abstract] OR "chronic fatigue and immune dysfunction syndrome*"[Title/Abstract]) OR (("fatiguability"[All Fields] OR "fatiguable"[All Fields] OR "Fatigue"[MeSH Terms] OR "Fatigue"[All Fields] OR "fatigued"[All Fields] OR "fatigues"[All Fields] OR "fatiguing"[All Fields] OR "fatigueability"[All Fields]) AND ("syndrom"[All Fields] OR "syndromal"[All Fields] OR "syndromally"[All Fields] OR "syndrome"[MeSH Terms] OR "syndrome"[All Fields] OR "syndromes"[All Fields] OR "syndrome s"[All Fields] OR "syndromic"[All Fields] OR "syndroms"[All Fields]))	130349
**11**	Medicine, Traditional[MeSH Terms]	Most Recent	/	"medicine, traditional"[MeSH Terms]	44856
**12**	Complementary Therapies[MeSH Terms]	Most Recent	/	"complementary therapies"[MeSH Terms]	242059
**13**	Phytotherapy[MeSH Terms]	Most Recent	/	"phytotherapy"[MeSH Terms]	41874
**14**	Plant Extracts[MeSH Terms]	Most Recent	/	"plant extracts"[MeSH Terms]	203704
**15**	Plants, Medicinal[MeSH Terms]	Most Recent	/	"plants, medicinal"[MeSH Terms]	62060

**16**	Plant Preparations[MeSH Terms]	Most Recent	/	"plant preparations"[MeSH Terms]	266122
**17**	"alternative medicine*"[Title/Abstract]	Most Recent	/	"alternative medicine*"[Title/Abstract]	11979
**18**	"folk remed*"[Title/Abstract] OR nutraceutical*[Title/Abstract] OR herb*[Title/Abstract] OR"medicinal plant*"[Title/Abstract] OR"pharmaceutical plant*"[Title/Abstract] OR"Chinese medicine*"[Title/Abstract]	Most Recent	/	"folk remed*"[Title/Abstract] OR "nutraceutical*"[Title/Abstract] OR "herb*"[Title/Abstract] OR "medicinal plant*"[Title/Abstract] OR "pharmaceutical plant*"[Title/Abstract] OR "chinese medicine*"[Title/Abstract]	193311

**19**	#10 OR #18	Most Recent	/	("Fatigue"[MeSH Terms] OR "Lassitude"[Title/Abstract] OR "Fatigue"[Title/Abstract] OR (("Chronic"[All Fields] OR "chronical"[All Fields] OR "chronically"[All Fields] OR "chronicities"[All Fields] OR "chronicity"[All Fields] OR "chronicization"[All Fields] OR "chronics"[All Fields]) AND "Fatigue"[MeSH Terms]) OR (("fatiguability"[All Fields] OR "fatiguable"[All Fields] OR "Fatigue"[MeSH Terms] OR "Fatigue"[All Fields] OR "fatigued"[All Fields] OR "fatigues"[All Fields] OR "fatiguing"[All Fields] OR "fatigueability"[All Fields]) AND ("syndrom"[All Fields] OR "syndromal"[All Fields] OR "syndromally"[All Fields] OR "syndrome"[MeSH Terms] OR "syndrome"[All Fields] OR "syndromes"[All Fields] OR "syndrome s"[All Fields] OR "syndromic"[All Fields] OR "syndroms"[All Fields])) OR "chronic fatigue"[Title/Abstract] OR "fatigue syndrome*"[MeSH Terms] OR (("Chronic"[All Fields] OR "chronical"[All Fields] OR "chronically"[All Fields] OR "chronicities"[All Fields] OR "chronicity"[All Fields] OR "chronicization"[All Fields] OR "chronics"[All Fields]) AND "fatigue syndrome*"[MeSH Terms]) OR (("Chronic"[All Fields] OR "chronical"[All Fields] OR "chronically"[All Fields] OR "chronicities"[All Fields] OR "chronicity"[All Fields] OR "chronicization"[All Fields] OR "chronics"[All Fields]) AND ("fatiguability"[All Fields] OR "fatiguable"[All Fields] OR "Fatigue"[MeSH Terms] OR "Fatigue"[All Fields] OR "fatigued"[All Fields] OR "fatigues"[All Fields] OR "fatiguing"[All Fields] OR "fatigueability"[All Fields]) AND ("Fibromyalgia"[MeSH Terms] OR "Fibromyalgia"[All Fields] OR "fibromyalgias"[All Fields]) AND "syndrome*"[MeSH Terms]) OR (("fatiguability"[All Fields] OR "fatiguable"[All Fields] OR "Fatigue"[MeSH Terms] OR "Fatigue"[All Fields] OR "fatigued"[All Fields] OR "fatigues"[All Fields] OR "fatiguing"[All Fields] OR "fatigueability"[All Fields]) AND "disorder*"[MeSH Terms]) OR ("Postviral"[All Fields] AND "fatigue syndrome*"[MeSH Terms]) OR (("Chronic"[All Fields] OR "chronical"[All Fields] OR "chronically"[All Fields] OR "chronicities"[All Fields] OR "chronicity"[All Fields] OR "chronicization"[All Fields] OR "chronics"[All Fields]) AND ("fatiguability"[All Fields] OR "fatiguable"[All Fields] OR "Fatigue"[MeSH Terms] OR "Fatigue"[All Fields] OR "fatigued"[All Fields] OR "fatigues"[All Fields] OR "fatiguing"[All Fields] OR "fatigueability"[All Fields]) AND ("immune system diseases"[MeSH Terms] OR ("Immune"[All Fields] AND "system"[All Fields] AND "diseases"[All Fields]) OR "immune system diseases"[All Fields] OR ("Immune"[All Fields] AND "Dysfunction"[All Fields]) OR "immune dysfunction"[All Fields]) AND "syndrome*"[MeSH Terms]) OR "fatigue syndrome*"[Title/Abstract] OR "chronic fatigue syndrome*"[Title/Abstract] OR "chronic fatigue fibromyalgia syndrome*"[Title/Abstract] OR "fatigue disorder*"[Title/Abstract] OR "postviral fatigue syndrome*"[Title/Abstract] OR "chronic fatigue and immune dysfunction syndrome*"[Title/Abstract]) AND ("medicine, traditional"[MeSH Terms] OR "complementary therapies"[MeSH Terms] OR "phytotherapy"[MeSH Terms] OR "plant extracts"[MeSH Terms] OR "plants, medicinal"[MeSH Terms] OR "plant preparations"[MeSH Terms] OR "drugs, chinese herbal"[MeSH Terms] OR "medicine, chinese traditional"[MeSH Terms] OR "complementary medicine*"[Title/Abstract] OR "alternative medicine*"[Title/Abstract] OR "chinese medicine*"[Title/Abstract] OR "pharmaceutical plant*"[Title/Abstract] OR "medicinal plant*"[Title/Abstract] OR "herb*"[Title/Abstract] OR "nutraceutical*"[Title/Abstract] OR "folk remed*"[Title/Abstract] OR "folk medicine*"[Title/Abstract])	4524

**20**	#19	Most Recent	Randomized Controlled Trial	(("Fatigue"[MeSH Terms] OR "Lassitude"[Title/Abstract] OR "Fatigue"[Title/Abstract] OR (("Chronic"[All Fields] OR "chronical"[All Fields] OR "chronically"[All Fields] OR "chronicities"[All Fields] OR "chronicity"[All Fields] OR "chronicization"[All Fields] OR "chronics"[All Fields]) AND "Fatigue"[MeSH Terms]) OR (("fatiguability"[All Fields] OR "fatiguable"[All Fields] OR "Fatigue"[MeSH Terms] OR "Fatigue"[All Fields] OR "fatigued"[All Fields] OR "fatigues"[All Fields] OR "fatiguing"[All Fields] OR "fatigueability"[All Fields]) AND ("syndrom"[All Fields] OR "syndromal"[All Fields] OR "syndromally"[All Fields] OR "syndrome"[MeSH Terms] OR "syndrome"[All Fields] OR "syndromes"[All Fields] OR "syndrome s"[All Fields] OR "syndromic"[All Fields] OR "syndroms"[All Fields])) OR "chronic fatigue"[Title/Abstract] OR "fatigue syndrome*"[MeSH Terms] OR (("Chronic"[All Fields] OR "chronical"[All Fields] OR "chronically"[All Fields] OR "chronicities"[All Fields] OR "chronicity"[All Fields] OR "chronicization"[All Fields] OR "chronics"[All Fields]) AND "fatigue syndrome*"[MeSH Terms]) OR ((("Chronic"[All Fields] OR "chronical"[All Fields] OR "chronically"[All Fields] OR "chronicities"[All Fields] OR "chronicity"[All Fields] OR "chronicization"[All Fields] OR "chronics"[All Fields]) AND ("fatiguability"[All Fields] OR "fatiguable"[All Fields] OR "Fatigue"[MeSH Terms] OR "Fatigue"[All Fields] OR "fatigued"[All Fields] OR "fatigues"[All Fields] OR "fatiguing"[All Fields] OR "fatigueability"[All Fields]) AND ("Fibromyalgia"[MeSH Terms] OR "Fibromyalgia"[All Fields] OR "fibromyalgias"[All Fields])) AND "syndrome*"[MeSH Terms]) OR (("fatiguability"[All Fields] OR "fatiguable"[All Fields] OR "Fatigue"[MeSH Terms] OR "Fatigue"[All Fields] OR "fatigued"[All Fields] OR "fatigues"[All Fields] OR "fatiguing"[All Fields] OR "fatigueability"[All Fields]) AND "disorder*"[MeSH Terms]) OR ("Postviral"[All Fields] AND "fatigue syndrome*"[MeSH Terms]) OR ((("Chronic"[All Fields] OR "chronical"[All Fields] OR "chronically"[All Fields] OR "chronicities"[All Fields] OR "chronicity"[All Fields] OR "chronicization"[All Fields] OR "chronics"[All Fields]) AND ("fatiguability"[All Fields] OR "fatiguable"[All Fields] OR "Fatigue"[MeSH Terms] OR "Fatigue"[All Fields] OR "fatigued"[All Fields] OR "fatigues"[All Fields] OR "fatiguing"[All Fields] OR "fatigueability"[All Fields]) AND ("immune system diseases"[MeSH Terms] OR ("Immune"[All Fields] AND "system"[All Fields] AND "diseases"[All Fields]) OR "immune system diseases"[All Fields] OR ("Immune"[All Fields] AND "Dysfunction"[All Fields]) OR "immune dysfunction"[All Fields])) AND "syndrome*"[MeSH Terms]) OR "fatigue syndrome*"[Title/Abstract] OR "chronic fatigue syndrome*"[Title/Abstract] OR "chronic fatigue fibromyalgia syndrome*"[Title/Abstract] OR "fatigue disorder*"[Title/Abstract] OR "postviral fatigue syndrome*"[Title/Abstract] OR "chronic fatigue and immune dysfunction syndrome*"[Title/Abstract]) AND ("medicine, traditional"[MeSH Terms] OR "complementary therapies"[MeSH Terms] OR "phytotherapy"[MeSH Terms] OR "plant extracts"[MeSH Terms] OR "plants, medicinal"[MeSH Terms] OR "plant preparations"[MeSH Terms] OR "drugs, chinese herbal"[MeSH Terms] OR "medicine, chinese traditional"[MeSH Terms] OR "complementary medicine*"[Title/Abstract] OR "alternative medicine*"[Title/Abstract] OR "chinese medicine*"[Title/Abstract] OR "pharmaceutical plant*"[Title/Abstract] OR "medicinal plant*"[Title/Abstract] OR "herb*"[Title/Abstract] OR "nutraceutical*"[Title/Abstract] OR "folk remed*"[Title/Abstract] OR "folk medicine*"[Title/Abstract])) AND (randomized controlled trial[Filter])	1011

**21**	#19	Most Recent	Clinical Trial, Randomized Controlled Trial	(("Fatigue"[MeSH Terms] OR "Lassitude"[Title/Abstract] OR "Fatigue"[Title/Abstract] OR (("Chronic"[All Fields] OR "chronical"[All Fields] OR "chronically"[All Fields] OR "chronicities"[All Fields] OR "chronicity"[All Fields] OR "chronicization"[All Fields] OR "chronics"[All Fields]) AND "Fatigue"[MeSH Terms]) OR (("fatiguability"[All Fields] OR "fatiguable"[All Fields] OR "Fatigue"[MeSH Terms] OR "Fatigue"[All Fields] OR "fatigued"[All Fields] OR "fatigues"[All Fields] OR "fatiguing"[All Fields] OR "fatigueability"[All Fields]) AND ("syndrom"[All Fields] OR "syndromal"[All Fields] OR "syndromally"[All Fields] OR "syndrome"[MeSH Terms] OR "syndrome"[All Fields] OR "syndromes"[All Fields] OR "syndrome s"[All Fields] OR "syndromic"[All Fields] OR "syndroms"[All Fields])) OR "chronic fatigue"[Title/Abstract] OR "fatigue syndrome*"[MeSH Terms] OR (("Chronic"[All Fields] OR "chronical"[All Fields] OR "chronically"[All Fields] OR "chronicities"[All Fields] OR "chronicity"[All Fields] OR "chronicization"[All Fields] OR "chronics"[All Fields]) AND "fatigue syndrome*"[MeSH Terms]) OR ((("Chronic"[All Fields] OR "chronical"[All Fields] OR "chronically"[All Fields] OR "chronicities"[All Fields] OR "chronicity"[All Fields] OR "chronicization"[All Fields] OR "chronics"[All Fields]) AND ("fatiguability"[All Fields] OR "fatiguable"[All Fields] OR "Fatigue"[MeSH Terms] OR "Fatigue"[All Fields] OR "fatigued"[All Fields] OR "fatigues"[All Fields] OR "fatiguing"[All Fields] OR "fatigueability"[All Fields]) AND ("Fibromyalgia"[MeSH Terms] OR "Fibromyalgia"[All Fields] OR "fibromyalgias"[All Fields])) AND "syndrome*"[MeSH Terms]) OR (("fatiguability"[All Fields] OR "fatiguable"[All Fields] OR "Fatigue"[MeSH Terms] OR "Fatigue"[All Fields] OR "fatigued"[All Fields] OR "fatigues"[All Fields] OR "fatiguing"[All Fields] OR "fatigueability"[All Fields]) AND "disorder*"[MeSH Terms]) OR ("Postviral"[All Fields] AND "fatigue syndrome*"[MeSH Terms]) OR ((("Chronic"[All Fields] OR "chronical"[All Fields] OR "chronically"[All Fields] OR "chronicities"[All Fields] OR "chronicity"[All Fields] OR "chronicization"[All Fields] OR "chronics"[All Fields]) AND ("fatiguability"[All Fields] OR "fatiguable"[All Fields] OR "Fatigue"[MeSH Terms] OR "Fatigue"[All Fields] OR "fatigued"[All Fields] OR "fatigues"[All Fields] OR "fatiguing"[All Fields] OR "fatigueability"[All Fields]) AND ("immune system diseases"[MeSH Terms] OR ("Immune"[All Fields] AND "system"[All Fields] AND "diseases"[All Fields]) OR "immune system diseases"[All Fields] OR ("Immune"[All Fields] AND "Dysfunction"[All Fields]) OR "immune dysfunction"[All Fields])) AND "syndrome*"[MeSH Terms]) OR "fatigue syndrome*"[Title/Abstract] OR "chronic fatigue syndrome*"[Title/Abstract] OR "chronic fatigue fibromyalgia syndrome*"[Title/Abstract] OR "fatigue disorder*"[Title/Abstract] OR "postviral fatigue syndrome*"[Title/Abstract] OR "chronic fatigue and immune dysfunction syndrome*"[Title/Abstract]) AND ("medicine, traditional"[MeSH Terms] OR "complementary therapies"[MeSH Terms] OR "phytotherapy"[MeSH Terms] OR "plant extracts"[MeSH Terms] OR "plants, medicinal"[MeSH Terms] OR "plant preparations"[MeSH Terms] OR "drugs, chinese herbal"[MeSH Terms] OR "medicine, chinese traditional"[MeSH Terms] OR "complementary medicine*"[Title/Abstract] OR "alternative medicine*"[Title/Abstract] OR "chinese medicine*"[Title/Abstract] OR "pharmaceutical plant*"[Title/Abstract] OR "medicinal plant*"[Title/Abstract] OR "herb*"[Title/Abstract] OR "nutraceutical*"[Title/Abstract] OR "folk remed*"[Title/Abstract] OR "folk medicine*"[Title/Abstract])) AND (clinicaltrial[Filter] OR randomizedcontrolledtrial[Filter])	1214

**22**	#19	Most Recent	Clinical Study, Clinical Trial, Randomized Controlled Trial	(("Fatigue"[MeSH Terms] OR "Lassitude"[Title/Abstract] OR "Fatigue"[Title/Abstract] OR (("Chronic"[All Fields] OR "chronical"[All Fields] OR "chronically"[All Fields] OR "chronicities"[All Fields] OR "chronicity"[All Fields] OR "chronicization"[All Fields] OR "chronics"[All Fields]) AND "Fatigue"[MeSH Terms]) OR (("fatiguability"[All Fields] OR "fatiguable"[All Fields] OR "Fatigue"[MeSH Terms] OR "Fatigue"[All Fields] OR "fatigued"[All Fields] OR "fatigues"[All Fields] OR "fatiguing"[All Fields] OR "fatigueability"[All Fields]) AND ("syndrom"[All Fields] OR "syndromal"[All Fields] OR "syndromally"[All Fields] OR "syndrome"[MeSH Terms] OR "syndrome"[All Fields] OR "syndromes"[All Fields] OR "syndrome s"[All Fields] OR "syndromic"[All Fields] OR "syndroms"[All Fields])) OR "chronic fatigue"[Title/Abstract] OR "fatigue syndrome*"[MeSH Terms] OR (("Chronic"[All Fields] OR "chronical"[All Fields] OR "chronically"[All Fields] OR "chronicities"[All Fields] OR "chronicity"[All Fields] OR "chronicization"[All Fields] OR "chronics"[All Fields]) AND "fatigue syndrome*"[MeSH Terms]) OR ((("Chronic"[All Fields] OR "chronical"[All Fields] OR "chronically"[All Fields] OR "chronicities"[All Fields] OR "chronicity"[All Fields] OR "chronicization"[All Fields] OR "chronics"[All Fields]) AND ("fatiguability"[All Fields] OR "fatiguable"[All Fields] OR "Fatigue"[MeSH Terms] OR "Fatigue"[All Fields] OR "fatigued"[All Fields] OR "fatigues"[All Fields] OR "fatiguing"[All Fields] OR "fatigueability"[All Fields]) AND ("Fibromyalgia"[MeSH Terms] OR "Fibromyalgia"[All Fields] OR "fibromyalgias"[All Fields])) AND "syndrome*"[MeSH Terms]) OR (("fatiguability"[All Fields] OR "fatiguable"[All Fields] OR "Fatigue"[MeSH Terms] OR "Fatigue"[All Fields] OR "fatigued"[All Fields] OR "fatigues"[All Fields] OR "fatiguing"[All Fields] OR "fatigueability"[All Fields]) AND "disorder*"[MeSH Terms]) OR ("Postviral"[All Fields] AND "fatigue syndrome*"[MeSH Terms]) OR ((("Chronic"[All Fields] OR "chronical"[All Fields] OR "chronically"[All Fields] OR "chronicities"[All Fields] OR "chronicity"[All Fields] OR "chronicization"[All Fields] OR "chronics"[All Fields]) AND ("fatiguability"[All Fields] OR "fatiguable"[All Fields] OR "Fatigue"[MeSH Terms] OR "Fatigue"[All Fields] OR "fatigued"[All Fields] OR "fatigues"[All Fields] OR "fatiguing"[All Fields] OR "fatigueability"[All Fields]) AND ("immune system diseases"[MeSH Terms] OR ("Immune"[All Fields] AND "system"[All Fields] AND "diseases"[All Fields]) OR "immune system diseases"[All Fields] OR ("Immune"[All Fields] AND "Dysfunction"[All Fields]) OR "immune dysfunction"[All Fields])) AND "syndrome*"[MeSH Terms]) OR "fatigue syndrome*"[Title/Abstract] OR "chronic fatigue syndrome*"[Title/Abstract] OR "chronic fatigue fibromyalgia syndrome*"[Title/Abstract] OR "fatigue disorder*"[Title/Abstract] OR "postviral fatigue syndrome*"[Title/Abstract] OR "chronic fatigue and immune dysfunction syndrome*"[Title/Abstract]) AND ("medicine, traditional"[MeSH Terms] OR "complementary therapies"[MeSH Terms] OR "phytotherapy"[MeSH Terms] OR "plant extracts"[MeSH Terms] OR "plants, medicinal"[MeSH Terms] OR "plant preparations"[MeSH Terms] OR "drugs, chinese herbal"[MeSH Terms] OR "medicine, chinese traditional"[MeSH Terms] OR "complementary medicine*"[Title/Abstract] OR "alternative medicine*"[Title/Abstract] OR "chinese medicine*"[Title/Abstract] OR "pharmaceutical plant*"[Title/Abstract] OR "medicinal plant*"[Title/Abstract] OR "herb*"[Title/Abstract] OR "nutraceutical*"[Title/Abstract] OR "folk remed*"[Title/Abstract] OR "folk medicine*"[Title/Abstract])) AND (clinical study[Filter] OR clinical trial[Filter] OR randomized controlled trial[Filter])	1309

**23**	#19 AND (clinical study[Filter] OR clinical trial[Filter] OR randomized controlled trial[Filter])	Most Recent	Clinical Study, Clinical Trial, Randomized Controlled Trial	(("Fatigue"[MeSH Terms] OR "Lassitude"[Title/Abstract] OR "Fatigue"[Title/Abstract] OR (("Chronic"[All Fields] OR "chronical"[All Fields] OR "chronically"[All Fields] OR "chronicities"[All Fields] OR "chronicity"[All Fields] OR "chronicization"[All Fields] OR "chronics"[All Fields]) AND "Fatigue"[MeSH Terms]) OR (("fatiguability"[All Fields] OR "fatiguable"[All Fields] OR "Fatigue"[MeSH Terms] OR "Fatigue"[All Fields] OR "fatigued"[All Fields] OR "fatigues"[All Fields] OR "fatiguing"[All Fields] OR "fatigueability"[All Fields]) AND ("syndrom"[All Fields] OR "syndromal"[All Fields] OR "syndromally"[All Fields] OR "syndrome"[MeSH Terms] OR "syndrome"[All Fields] OR "syndromes"[All Fields] OR "syndromes"[All Fields] OR "syndromic"[All Fields] OR "syndroms"[All Fields])) OR "chronic fatigue"[Title/Abstract] OR "fatigue syndrome*"[MeSH Terms] OR (("Chronic"[All Fields] OR "chronical"[All Fields] OR "chronically"[All Fields] OR "chronicities"[All Fields] OR "chronicity"[All Fields] OR "chronicization"[All Fields] OR "chronics"[All Fields]) AND "fatigue syndrome*"[MeSH Terms]) OR ((("Chronic"[All Fields] OR "chronical"[All Fields] OR "chronically"[All Fields] OR "chronicities"[All Fields] OR "chronicity"[All Fields] OR "chronicization"[All Fields] OR "chronics"[All Fields]) AND ("fatiguability"[All Fields] OR "fatiguable"[All Fields] OR "Fatigue"[MeSH Terms] OR "Fatigue"[All Fields] OR "fatigued"[All Fields] OR "fatigues"[All Fields] OR "fatiguing"[All Fields] OR "fatigueability"[All Fields]) AND ("Fibromyalgia"[MeSH Terms] OR "Fibromyalgia"[All Fields] OR "fibromyalgias"[All Fields])) AND "syndrome*"[MeSH Terms]) OR (("fatiguability"[All Fields] OR "fatiguable"[All Fields] OR "Fatigue"[MeSH Terms] OR "Fatigue"[All Fields] OR "fatigued"[All Fields] OR "fatigues"[All Fields] OR "fatiguing"[All Fields] OR "fatigueability"[All Fields]) AND "disorder*"[MeSH Terms]) OR ("Postviral"[All Fields] AND "fatigue syndrome*"[MeSH Terms]) OR ((("Chronic"[All Fields] OR "chronical"[All Fields] OR "chronically"[All Fields] OR "chronicities"[All Fields] OR "chronicity"[All Fields] OR "chronicization"[All Fields] OR "chronics"[All Fields]) AND ("fatiguability"[All Fields] OR "fatiguable"[All Fields] OR "Fatigue"[MeSH Terms] OR "Fatigue"[All Fields] OR "fatigued"[All Fields] OR "fatigues"[All Fields] OR "fatiguing"[All Fields] OR "fatigueability"[All Fields]) AND ("immune system diseases"[MeSH Terms] OR ("Immune"[All Fields] AND "system"[All Fields] AND "diseases"[All Fields]) OR "immune system diseases"[All Fields] OR ("Immune"[All Fields] AND "Dysfunction"[All Fields]) OR "immune dysfunction"[All Fields])) AND "syndrome*"[MeSH Terms]) OR "fatigue syndrome*"[Title/Abstract] OR "chronic fatigue syndrome*"[Title/Abstract] OR "chronic fatigue fibromyalgia syndrome*"[Title/Abstract] OR "fatigue disorder*"[Title/Abstract] OR "postviral fatigue syndrome*"[Title/Abstract] OR "chronic fatigue and immune dysfunction syndrome*"[Title/Abstract]) AND ("medicine, traditional"[MeSH Terms] OR "complementary therapies"[MeSH Terms] OR "phytotherapy"[MeSH Terms] OR "plant extracts"[MeSH Terms] OR "plants, medicinal"[MeSH Terms] OR "plant preparations"[MeSH Terms] OR "drugs, chinese herbal"[MeSH Terms] OR "medicine, chinese traditional"[MeSH Terms] OR "complementary medicine*"[Title/Abstract] OR "alternative medicine*"[Title/Abstract] OR "chinese medicine*"[Title/Abstract] OR "pharmaceutical plant*"[Title/Abstract] OR "medicinal plant*"[Title/Abstract] OR "herb*"[Title/Abstract] OR "nutraceutical*"[Title/Abstract] OR "folk remed*"[Title/Abstract] OR "folk medicine*"[Title/Abstract]) AND ("clinical study"[Publication Type] OR "clinical trial"[Publication Type] OR "randomized controlled trial"[Publication Type])) AND (clinical study[Filter] OR clinical trial[Filter] OR randomized controlled trial[Filter])	1309

Supplementary Table.2 Web of Science (WOS)-Search history

#Number	Search queries	Database	Result	Running date
**11**	#9 AND #8 AND #7 and **Article** (Document Types)	Web of Science core collection	272	Sun Dec 25 2022 15:07:02 GMT+0800 (China Standard Time)
**10**	#9 AND #8 AND #7	Web of Science core collection	413	Sun Dec 25 2022 15:07:02 GMT+0800 (China Standard Time)
**9**	TS=("clinical trial*" OR "clinical stud*")	Web of Science core collection	654430	Sun Dec 25 2022 15:07:02 GMT+0800 (China Standard Time)
**8**	TS=("traditional medicine*" OR "complementary medicine*" OR "complementary therap*" OR "phytotherapy" OR "plant extract*" OR "herb*" OR "chinese medicine*" OR "alternative medicine*" OR "pharmaceutical plant*" OR "medicinal plant*" OR "nutraceutical*" OR "folk")	Web of Science core collection	478313	Sun Dec 25 2022 15:07:02 GMT+0800 (China Standard Time)
**7**	#6 OR #5	Web of Science core collection	302532	Sun Dec 25 2022 15:07:02 GMT+0800 (China Standard Time)
**6**	TS=("Fatigue Syndrome*" OR "Chronic Fatigue Syndrome*" OR "Chronic Fatigue Fibromyalgia Syndrome*" OR "Fatigue Disorder*" OR "Postviral Fatigue Syndrome*" OR "Chronic Fatigue and Immune Dysfunction Syndrome*")	Web of Science core collection	9838	Sun Dec 25 2022 15:07:02 GMT+0800 (China Standard Time)
**5**	#3 OR #4	Web of Science core collection	302532	Sun Dec 25 2022 15:07:02 GMT+0800 (China Standard Time)
**4**	TS=(chronic fatigue)	Web of Science core collection	24779	Sun Dec 25 2022 15:07:02 GMT+0800 (China Standard Time)
**3**	#2 OR #1	Web of Science core collection	302532	Sun Dec 25 2022 15:07:02 GMT+0800 (China Standard Time)
**2**	TS=(lassitude)	Web of Science core collection	327	Sun Dec 25 2022 15:07:02 GMT+0800 (China Standard Time)
**1**	TS=(fatigue)	Web of Science core collection	302279	Sun Dec 25 2022 15:07:02 GMT+0800 (China Standard Time)

Supplementary Table.3 Scopus-Search history

Number	Query	Results
**13**	(((TITLE-ABS-KEY (lassitude)) OR (TITLE-ABS-KEY (chronic AND fatigue)) OR (TITLE-ABS-KEY (chronic W/0 fatigue)) OR (TITLE-ABS-KEY ("chronic fatigue")) OR (TITLE-ABS-KEY ("Fatigue Syndrome*" OR "Chronic Fatigue Syndrome*" OR "Chronic Fatigue Fibromyalgia Syndrome*" OR "Fatigue Disorder*" OR "Postviral Fatigue Syndrome*" OR "Chronic Fatigue and Immune Dysfunction Syndrome*"))) OR (TITLE-ABS-KEY (fatigue))) AND (TITLE-ABS-KEY ("traditional medicine*" OR "complementary medicine*" OR "complementary therap*" OR "phytotherapy" OR "plant extract*" OR "herb*" OR "chinese medicine*" OR "alternative medicine*" OR "pharmaceutical plant*" OR "medicinal plant*" OR "nutraceutical*" OR "folk")) AND (TITLE-ABS-KEY ("clinical trial*" OR "clinical stud*")) AND (LIMIT-TO (DOCTYPE , "ar")) AND (LIMIT-TO (LANGUAGE , "English") OR LIMIT-TO (LANGUAGE , "Chinese"))	1733
**12**	(((TITLE-ABS-KEY (lassitude)) OR (TITLE-ABS-KEY (chronic AND fatigue)) OR (TITLE-ABS-KEY (chronic W/0 fatigue)) OR (TITLE-ABS-KEY ("chronic fatigue")) OR (TITLE-ABS-KEY ("Fatigue Syndrome*" OR "Chronic Fatigue Syndrome*" OR "Chronic Fatigue Fibromyalgia Syndrome*" OR "Fatigue Disorder*" OR "Postviral Fatigue Syndrome*" OR "Chronic Fatigue and Immune Dysfunction Syndrome*"))) OR (TITLE-ABS-KEY (fatigue))) AND (TITLE-ABS-KEY ("traditional medicine*" OR "complementary medicine*" OR "complementary therap*" OR "phytotherapy" OR "plant extract*" OR "herb*" OR "chinese medicine*" OR "alternative medicine*" OR "pharmaceutical plant*" OR "medicinal plant*" OR "nutraceutical*" OR "folk")) AND (TITLE-ABS-KEY ("clinical trial*" OR "clinical stud*")) AND (LIMIT-TO (DOCTYPE , "ar"))	1784
**11**	(((TITLE-ABS-KEY (lassitude)) OR (TITLE-ABS-KEY (chronic AND fatigue)) OR (TITLE-ABS-KEY (chronic W/0 fatigue)) OR (TITLE-ABS-KEY ("chronic fatigue")) OR (TITLE-ABS-KEY ("Fatigue Syndrome*" OR "Chronic Fatigue Syndrome*" OR "Chronic Fatigue Fibromyalgia Syndrome*" OR "Fatigue Disorder*" OR "Postviral Fatigue Syndrome*" OR "Chronic Fatigue and Immune Dysfunction Syndrome*"))) OR (TITLE-ABS-KEY (fatigue))) AND (TITLE-ABS-KEY ("traditional medicine*" OR "complementary medicine*" OR "complementary therap*" OR "phytotherapy" OR "plant extract*" OR "herb*" OR "chinese medicine*" OR "alternative medicine*" OR "pharmaceutical plant*" OR "medicinal plant*" OR "nutraceutical*" OR "folk")) AND (TITLE-ABS-KEY ("clinical trial*" OR "clinical stud*"))	2570

**10**	TITLE-ABS-KEY ("clinical trial*" OR "clinical stud*")	5141802
**9**	TITLE-ABS-KEY ("traditional medicine*" OR "complementary medicine*" OR "complementary therap*" OR "phytotherapy" OR "plant extract*" OR "herb*" OR "chinese medicine*" OR "alternative medicine*" OR "pharmaceutical plant*" OR "medicinal plant*" OR "nutraceutical*" OR "folk")	851841
**8**	((TITLE-ABS-KEY (lassitude)) OR (TITLE-ABS-KEY (chronic AND fatigue)) OR (TITLE-ABS-KEY (chronic W/0 fatigue)) OR (TITLE-ABS-KEY ("chronic fatigue")) OR (TITLE-ABS-KEY ("Fatigue Syndrome*" OR "Chronic Fatigue Syndrome*" OR "Chronic Fatigue Fibromyalgia Syndrome*" OR "Fatigue Disorder*" OR "Postviral Fatigue Syndrome*" OR "Chronic Fatigue and Immune Dysfunction Syndrome*"))) OR (TITLE-ABS-KEY (fatigue))	518102
**7**	(TITLE-ABS-KEY (lassitude)) OR (TITLE-ABS-KEY (chronic AND fatigue)) OR (TITLE-ABS-KEY (chronic W/0 fatigue)) OR (TITLE-ABS-KEY ("chronic fatigue")) OR (TITLE-ABS-KEY ("Fatigue Syndrome*" OR "Chronic Fatigue Syndrome*" OR "Chronic Fatigue Fibromyalgia Syndrome*" OR "Fatigue Disorder*" OR "Postviral Fatigue Syndrome*" OR "Chronic Fatigue and Immune Dysfunction Syndrome*"))	49244
**6**	TITLE-ABS-KEY ("Fatigue Syndrome*" OR "Chronic Fatigue Syndrome*" OR "Chronic Fatigue Fibromyalgia Syndrome*" OR "Fatigue Disorder*" OR "Postviral Fatigue Syndrome*" OR "Chronic Fatigue and Immune Dysfunction Syndrome*")	12886
**5**	TITLE-ABS-KEY ("chronic fatigue")	13672
**4**	TITLE-ABS-KEY (chronic W/0 fatigue)	13728
**3**	TITLE-ABS-KEY (chronic AND fatigue)	47687
**2**	TITLE-ABS-KEY (lassitude)	1248
**1**	TITLE-ABS-KEY (fatigue)	517143

Supplementary Table.4 Embase-Search history

Number	Query	Results
**#26**	#23 AND [humans]/lim AND [article]/lim AND [clinical study]/lim	1020
**#25**	#23 NOT #24	2003
**#24**	'massage':ti,ab,kw OR 'yoga':ti,ab,kw OR 'acupuncture':ti,ab,kw OR 'music':ti,ab,kw OR 'moxibustion':ti,ab,kw OR 'meditation':ti,ab,kw OR 'exercise':ti,ab,kw OR 'relaxation':ti,ab,kw OR 'aromatherapy':ti,ab,kw OR 'hypnosis':ti,ab,kw	662591
**#23**	#6 AND #12 NOT #20 AND ([chinese]/lim OR [english]/lim) AND [humans]/lim	2534
**#22**	#6 AND #12 NOT #20 AND ([chinese]/lim OR [english]/lim)	3094
**#21**	#6 AND #12 NOT #20	3243
**#20**	protocol:ti OR pilot:ti OR review:ti OR retrospective:ti OR observational:ti OR 'cross-sectional study':ti,ab,kw OR questionnaire:ti,ab,kw OR 'meta analysis':ti,ab,kw	2249842
**#19**	#6 AND #12 AND [article]/lim AND [humans]/lim AND [clinical study]/lim AND ([randomized controlled trial]/lim OR 'controlled clinical trial'/de) AND ([chinese]/lim OR [english]/lim)	559
**#18**	#6 AND #12 AND [article]/lim AND [humans]/lim AND [clinical study]/lim	1482
**#17**	#13 AND #14 AND ([chinese]/lim OR [english]/lim) AND [humans]/lim	732
**#16**	#13 AND #14 AND ([chinese]/lim OR [english]/lim)	756
**#15**	#13 AND #14	776

**#14**	clinical trial*':ti,ab,kw OR 'clinical stud*':ti,ab,kw	897220
**#13**	#6 AND #12	4472
**#12**	#10 OR #11	287601
**#11**	complementary medicine*':ti,ab,kw OR 'medicinal plant*':ti,ab,kw OR 'herb*':ti,ab,kw	209188
**#10**	#7 OR #8 OR #9	145857
**#9**	complementary therap*':ti,ab,kw OR 'nutraceutical*':ti,ab,kw OR 'folk*':ti,ab,kw	40500
**#8**	chinese medicine*':ti,ab,kw OR herb:ti,ab,kw OR 'phytotherapy*':ti,ab,kw OR 'plant extract*':ti,ab,kw	101049
**#7**	traditional medicine*':ti,ab,kw OR 'alternative medicine*':ti,ab,kw OR 'pharmaceutical plant*':ti,ab,kw	42252
**#6**	#1 OR #2 OR #3 OR #4 OR #5	346519
**#5**	chronic fatigue syndrome':ti,ab,kw OR 'postviral fatigue syndrome':ti,ab,kw OR ('chronic fatigue':ti,ab,kw AND 'immune dysfunction syndrome':ti,ab,kw)	8192
**#4**	chronic fatigue syndrome':ti,ab,kw	8113
**#3**	fatigue:ti,ab,kw OR lassitude:ti,ab,kw	194112
**#2**	lassitude	1244
**#1**	fatigue'/exp OR fatigue	346274

Supplementary Table.5 Cochrane-Search history

ID	Search	Hits
**#14**	#12 AND #13 in Trials	687
**#13**	#4 OR #5 OR #6 OR #7 OR #9 OR #10 OR #11	53425
**#12**	#1 OR #2 OR #3 OR #8	6366
**#11**	(chinese medicine*):ti,ab,kw OR (phytotherapy*):ti,ab,kw OR (plant extract*):ti,ab,kw	22773
**#10**	(complementary therap*):ti,ab,kw OR (nutraceutical*):ti,ab,kw OR (folk*):ti,ab,kw	6309
**#9**	(complementary medicine*):ti,ab,kw OR (medicinal plant*):ti,ab,kw OR (herb*):ti,ab,kw	16544
**#8**	(chronic fatigue syndrome*):ti,ab,kw OR (postviral fatigue syndrome*):ti,ab,kw OR ((chronic fatigue*):ti,ab,kw AND(immune dysfunction syndrome):ti,ab,kw)	2051
**#7**	MeSH descriptor: [Plant Extracts] explode all trees	8969
**#6**	MeSH descriptor: [Phytotherapy] explode all trees	4337
**#5**	MeSH descriptor: [Complementary Therapies] explode all trees	22366
**#4**	MeSH descriptor: [Medicine, Traditional] explode all trees	1654
**#3**	MeSH descriptor: [Fatigue Syndrome, Chronic] explode all trees	437
**#2**	Lassitude in Trials	161
**#1**	MeSH descriptor: [Fatigue] explode all trees	4378

Supplementary Table.6 Wanfang-Search history

序号	文献类型	检索式	检索结果
**8**	期刊	(主题:(中药 or 药物疗法 or 植物中药 or 饮片 or 中药配方颗粒 or 草药) or 题名或关键词: (中药 or 药物疗法 or 植物中药 or 饮片 or 中药配方颗粒 or 草药)) and (主题:(慢性疲劳综合征 or 肌痛性脑脊髓炎 or 肌痛性脑脊髓炎 or 慢性疲劳免疫功能紊乱综合征 or 病毒感染后疲劳综合征 or CFS) or 题名或关键词: (慢性疲劳综合征 or 疲劳 or 肌痛性脑脊髓炎 or 慢性疲劳免疫功能紊乱综合征 or 病毒感染后疲劳综合征 or CFS)) and (主题:(临床 or 实验 or 试验 or 疗效 or 观察) or 题名或关键词: ((临床 or 实验 or 试验 or 疗效 or 观察)))	620
**7**	期刊	主题:(慢性疲劳综合征 or 肌痛性脑脊髓炎 or 疲劳 or 慢性疲劳免疫功能紊乱综合征 or 病毒感染后疲劳综合征) or 题名或关键词:(慢性疲劳综合征 or 肌痛性脑脊髓炎 or 疲劳 or 慢性疲劳免疫功能紊乱综合征 or 病毒感染后疲劳综合征)	118391
**6**	期刊	(主题:(中药 or 药物疗法 or 植物中药 or 饮片 or 中药配方颗粒 or 草药) or 题名或关键词: (中药 or 药物疗法 or 植物中药 or 饮片 or 中药配方颗粒 or 草药)) and (主题:(慢性疲劳综合征 or 肌痛性脑脊髓炎 or 肌痛性脑脊髓炎 or 慢性疲劳免疫功能紊乱综合征 or 病毒感染后疲劳综合征 or CFS) or 题名或关键词: (慢性疲劳综合征 or 肌痛性脑脊髓炎 or 肌痛性脑脊髓炎 or 慢性疲劳免疫功能紊乱综合征 or 病毒感染后疲劳综合征 or CFS)) and (主题:(临床 or 实验 or 试验 or 疗效 or 观察) or 题名或关键词: ((临床 or 实验 or 试验 or 疗效 or 观察)))	148
**5**	期刊	(主题:(中药 or 药物疗法 or 植物中药 or 饮片 or 中药配方颗粒 or 草药) or 题名或关键词: (中药 or 药物疗法 or 植物中药 or 饮片 or 中药配方颗粒 or 草药)) and (主题:(慢性疲劳综合征 or 肌痛性脑脊髓炎 or 肌痛性脑脊髓炎 or 慢性疲劳免疫功能紊乱综合征 or 病毒感染后疲劳综合征 or CFS) or 题名或关键词: (慢性疲劳综合征 or 肌痛性脑脊髓炎 or 肌痛性脑脊髓炎 or 慢性疲劳免疫功能紊乱综合征 or 病毒感染后疲劳综合征 or CFS))	178
**4**	期刊	主题:(中药 or 药物疗法 or 植物中药 or 饮片 or 中药配方颗粒 or 草药) or 题名或关键词: (中药 or 药物疗法 or 植物中药 or 饮片 or 中药配方颗粒 or 草药)	465799
**3**	期刊	主题:(慢性疲劳综合征 or 肌痛性脑脊髓炎 or 肌痛性脑脊髓炎 or 慢性疲劳免疫功能紊乱综合征 or 病毒感染后疲劳综合征 or CFS) or 题名或关键词: (慢性疲劳综合征 or 肌痛性脑脊髓炎 or 肌痛性脑脊髓炎 or 慢性疲劳免疫功能紊乱综合征 or 病毒感染后疲劳综合征 or CFS)	4904
**2**	期刊	主题:(慢性疲劳综合征 or 肌痛性脑脊髓炎 or 肌痛性脑脊髓炎 or 慢性疲劳免疫功能紊乱综合征 or 病毒感染后疲劳综合征) or 题名或关键词: (慢性疲劳综合征 or 肌痛性脑脊髓炎 or 肌痛性脑脊髓炎 or 慢性疲劳免疫功能紊乱综合征 or 病毒感染后疲劳综合征 or CFS)	2476
**1**	期刊	主题:(临床 or 实验 or 试验 or 疗效 or 观察) or 题名或关键词: ((临床 or 实验 or 试验 or 疗效 or 观察))	10428108

Supplementary Table.7 CNKI-Search history

序号	检索式	检索范围	数量
**10**	(主題=临床 + 实验 + 试验 + 疗效 + 观察) AND (主題=中药 + 药物疗法 + 植物中药 + 饮片 + 中药配方颗粒 + 草药) AND (主題=疲劳 +慢性疲劳综合征 + 肌痛性脑脊髓炎+ 慢性疲劳免疫功能紊乱综合征 + 病毒感染后疲劳综合征)	資源範圍:學術期刊；同義詞擴展；更新時間:不限；來源類別:全部期刊	529
**9**	(篇關摘=临床 + 实验 + 试验 + 疗效 + 观察) AND (篇關摘=中药 + 药物疗法 + 植物中药 + 饮片 + 中药配方颗粒 + 草药) AND (篇關摘=慢性疲劳综合征 + 肌痛性脑脊髓炎 + 慢性疲劳免疫功能紊乱综合征 + 病毒感染后疲劳综合征 + CFS)	資源範圍:學術期刊；同義詞擴展；更新時間:不限；來源類別:全部期刊	184
**8**	(篇關摘=临床 + 实验 + 试验 + 疗效 + 观察) AND (篇關摘=中药 + 药物疗法 + 植物中药 + 饮片 + 中药配方颗粒 + 草药) AND (篇關摘=慢性疲劳综合征 + 肌痛性脑脊髓炎 + 慢性疲劳免疫功能紊乱综合征 + 病毒感染后疲劳综合征)	資源範圍:學術期刊；同義詞擴展；更新時間:不限；來源類別:全部期刊	147
**7**	(主題=临床 + 实验 + 试验 + 疗效 + 观察) AND (主題=中药 + 药物疗法 + 植物中药 + 饮片 + 中药配方颗粒 + 草药) AND (主題=慢性疲劳综合征 + 肌痛性脑脊髓炎+ 慢性疲劳免疫功能紊乱综合征 + 病毒感染后疲劳综合征)	資源範圍:學術期刊；同義詞擴展；更新時間:不限；來源類別:全部期刊	93
**6**	(主題=临床 + 实验 + 试验 + 疗效 + 观察) AND (主題=中药 + 药物疗法 + 植物中药 + 饮片 + 中药配方颗粒 + 草药) AND (主題=慢性疲劳综合征 + 肌痛性脑脊髓炎 + 慢性疲劳免疫功能紊乱综合征 + 病毒感染后疲劳综合征)	資源範圍:學術期刊；中英文擴展；更新時間:不限；來源類別:全部期刊	89
**5**	(主題=临床 + 实验 + 试验 + 疗效)	資源範圍:學術期刊；中英文擴展；更新時間:不限；來源類別:全部期刊	8,326,813
**4**	(主題=临床 + 实验 + 试验)	資源範圍:學術期刊；中英文擴展；更新時間:不限；來源類別:全部期刊	7,851,124
**3**	(主題=中药 + 药物疗法 + 植物中药 + 饮片 + 中药配方颗粒 + 草药)	資源範圍:學術期刊；中英文擴展；更新時間:不限；來源類別:全部期刊	361,343
**2**	(主題=慢性疲劳综合征 + 肌痛性脑脊髓炎 + 慢性疲劳免疫功能紊乱综合征 + 病毒感染后疲劳综合征)	資源範圍:學術期刊；中英文擴展；更新時間:不限；來源類別:全部期刊	1,829
**1**	(主題=疲劳)	資源範圍:學術期刊；中英文擴展；更新時間:不限；來源類別:全部期刊	104,015
