# Supplementary material for: Is there a role for traditional and complementary medicines in managing chronic fatigue? a systematic review of randomized controlled trials
Source: Front Pharmacol. 2023 Oct 24;14:1266803. doi: 10.3389/fphar.2023.1266803 (PMC10628447; doi:10.3389/fphar.2023.1266803)
Supplement: Supplementary file 2 [file DataSheet2.docx]

**Supplementary Table 8. Description of the studies included**

| Reference | Country | Language | Hospital | Analyzed  population | Sample size(T/C) | Gender (M/F) | | Age range(mean) | CFS criteria | CFS history  (mean) | Prescription name | Treatment  Duration | Follow-up |
| --- | --- | --- | --- | --- | --- | --- | --- | --- | --- | --- | --- | --- | --- |
| Zhou et al.,2022 | China | Chinese | S | 147 | 74,73 | | 41,106 | T:20-60(34.57±4.42)y C:20-60(34.61±4.58)y | TCM | T:8-45(28.12±2.45) m C:7-43(28.09±2.47) m | Xinshen’an Capsule | 8weeks | None |
| Wu et al.,2022 | China | Chinese | M | 100^#1^ | 50,50 | | 43,57 | T:(49.08±14.59)y C:(48.72±13.99)y | MM | T:(7.33±6.19) m C:(8.11±7.02) m | Dispelling Dampness-Replenishing Qi-Nourishing Yin Step Therapy | 9weeks | None |
| Ma et al.,2022 | China | Chinese | M | 100^#2^ | 50,50 | | 43,57 | T:(49.08±14.59)y C:(48.72±13.99)y | MM/TCM | T:(7.33±6.19) m C:(8.11±7.02) m | Dispelling Dampness-Replenishing Qi-Nourishing Yin Step Therapy | 9weeks | None |
| Li et al.,2022 | China | Chinese | S | 82^#3^ | 41,41 | | 41,41 | T:(47.81±6.72)y C:(48.02±7.50)y | MM/TCM | T:(25.90±4.70) m C:(26.07±4.62) m | Guashen Decoction | 4weeks | None |
| Huang et al.,2022 | China | Chinese | S | 60 | 30,30 | | 32,28 | T:23-50(34.10±6.98)y C:24-51(37.674±8.85)y | MM/TCM | T:7-18(11.37±3.32) m C:7-20(10.30±2.79) m | Wenzhen Yunqi Formula | 12weeks | None |
| Guo and Huang,2022 | China | Chinese | S | 60 | 30,30 | | 35,25 | T:45-70(57.62±3.14)y C:48-69(57.75±3.04)y | MM/TCM | T:8-30(19.82±3.01) m C:10-28(19.89±2.85) m | Fali Decoction | 1month | None |
| Liu et al.,2021 | China | Chinese | S | 72 | 36,36 | | N/A | 18-65y | MM/TCM | ≥6m | Chaihu Guizhi Decoction | 28days | after 1month |
| Chen ,2021 | China | Chinese | S | 63 | 33,30 | | 29,34 | T:24-54(33.8±13.1)y C:21-52(33.6±13.2)y | MM/TCM | T:6-34(18.51±9.03) m C:6-32(16.32±8.94) m | Xiaoyao powder | 60days | None |
| Shin et al., 2021 | Korea | English | M | 96 | 48,48 | | 29,67 | T:(41.5±8.2)y C:(40.6±9.8)y | MM | ≥6m | Sipjeondaebo-tang | 8weeks | None |
| Kan et al., 2021; | China | English | S | 175^#4^ | 59,58,58 | | 84,91 | T(H):(50.5±7.0)y T(L):(51.5±7.5)y C:(50.7±7.6)y | MM | ≥6m | Cistanche and Ginkgo extracts | 2months |  |
| Zhang, 2020 | China | Chinese | S | 58 | 29,29 | | 37,21 | T:22-70(38.45±12.15)y C:23-71(39.44±12.18)y | MM | N/A | Buzhong Yiqi Decotion Combine with Xiaochaihu Decotion | 14days | None |
| Sheng, 2020 | China | Chinese | S | 48 | 24,24 | | 27,21 | T:25-65(53.33±2.50)y C:26-64(53.35±2.51)y | MM/TCM | T:5-25(13.53±1.95) m C:6-24(13.52±1.94) m | Chaihu Guizhi Decoction | 28days | None |
| Mao, 2020 | China | Chinese | S | 59 | 30,29 | | 35,24 | T:21-64(39.58±0.46) y C:22-66(39.40±0.37)y | TCM | T:0.4-4.3(1.26±0.38) y C:0.5-4.1(1.37±0.22) y | Yishen Tiaodu Method | 8weeks | None |
| Li, 2020 | China | Chinese | S | 72 | 36,36 | | 40,32 | T:25-58(37.82±6.03)y C:27-63(39.11±5.94)y | TCM | N/A | Buzhong Yiqi Decotion Combine withXiaochaihu Decotion | 1month | None |
| Huang et al.,2020 | China | Chinese | S | 80^#5^ | 43,45 | | 20,68 | T:(36.2±7.5)y C:(39.5±10.9)y | MM/TCM | ≥6m | Yangwei Jianpi Plaster | 1month | None |
| Dong, 2020 | China | Chinese | S | 80 | 40,40 | | 49,31 | T:(37.72±3.34)y C:(37.68±3.41)y | MM/TCM | T:(1.21±0.15) y C:(1.24±0.17) y | Qingshu Yiqi Decotion | 3months | None |
| Sung et al., 2020 | Korea | English | S | 47^#6^ | 24,23 | | 14,33 | T:(49.000±8.351)y C:(47.087±10.795)y | MM | T:(6.038±7.686) y C:(5.730±7.055) y | Korean red ginseng (KRG) | 6weeks | 4weeks |
| Yang, 2019 | China | Chinese | S | 40 | 20,20 | | 21,19 | T:32-65(38.45±5.36)y C:29-66(39.12±5.21)y | MM/TCM | 6-18m | Zuogui Pills | 4months | None |
| Wang, 2019 | China | Chinese | S | 80 | 40,40 | | 44,36 | T:(43.5±8.7)y C:(42.7±8.5)y | TCM | T:(2.5±0.9) y C:(2.4±1.1) y | Buzhong Yiqi Decotion Combine with Xiaochaihu Decotion | 2weeks | None |
| Shi, 2019 | China | chinese | S | 160 | 78.82 | | 67,93 | T:19-58(41.51±9.347)y C:19-58(40.55±9.775)y | MM/TCM | T:0.9-3y C:0.7-2y | Modified Xiaoyao San | 3weeks | None |
| Ma et al.,2019 | China | Chinese | M | 80 | 40,40 | | 22,58 | T:19-66(mean=45.2)y C:20-67(mean=43)y | MM/TCM | ≥6m | Modified Erxian Decoction | 2months | None |
| Liu et al.,2019a | China | Chinese | S | 60 | 30,30 | | 21,39 | T:40-55(43.3±12.6)y C:40-55(42.9±10.6)y | MM/TCM | T:6m-3y (15.0±5.6) m C:10m-3y (16.0±6.3) m | Jianpi Yishen Decoction | 6weeks | None |
| Liu et al.,2019b | China | chinese | S | 72 | 36,36 | | 20,52 | T:20-65y C:27-65y | MM/TCM | T:7-24m C:7-26m | Chaihu Guizhi Decoction | 28days | 1month |
| Liu et al.,2019c | China | Chinese | S | 72 | 36,36 | | 20,52 | T:20-65y C:27-65y | MM/TCM | T:7-24m C:7-26m | Chaihu Guizhi Decoction | 28days | after 1month |
| Liu et al.,2019d | China | Chinese | S | 60 | 30,30 | | 30,30 | 28-67(mean=42)y | MM | 8m-3y (mean=1y) | Modified Lingzhi Pills | 1month | None |
| Lin et al.,2019 | China | chinese | S | 100 | 50,50 | | 52,48 | T:22-69(44.43±1.35)y C:23-68(40.05±0.59)y | MM/TCM | ≥6m | Self made fatigue Decoction | 1month | None |
| Li et al.,2019 | China | Chinese | S | 70 | 35,35 | | 28,42 | T:30-51y C:31-50y | MM/TCM | T:9-48m C:8-45m | Dalishen Tea | 4weeks | None |
| Hu, 2019 | China | Chinese | S | 66 | 33,33 | | 33,33 | T:46-66(median55.14±1.26)y C:45-65(median55.11±1.22)y | TCM | T:1-7(3.15±1.14) y C:1-7(3.11±1.11) y | Buzhong Yiqi Decotion Combine with Xiaochaihu Decotion | N/A | None |
| Ding, 2019 | China | Chinese | S | 60 | 30,30 | | 31,29 | T:20-60(39.21±1.25)y C:21-60(41.15±1.29)y | MM | ≥6m | Guipi Decoction | 12weeks | None |
| Joung et al., 2019 | Korea | English | M | 97^#7^ | 48,49 | | 37,60 | T:23–58(39.8±8.7)y C:21–64(39.5±11.1)y | MM | ≥6m | Myelophil | 12weeks | None |
| Wu et al.,2018 | China | Chinese | S | 86 | 43,43 | | 34,62 | T:24-68(39.7±6.9)y C:22-65(40.3±7.5)y | MM | T:6m-5y C:6m-7y | Guipi Decoction | T:12 weeks C:8weeks | None |
| Ou et al.,2018 | China | Chinese | S | 80 | 40,40 | | 37,43 | T:(50.3±11.35)y C:(49.8±10.45)y | MM/TCM | T:2-5y C:2-6y | Guipi Decoction | 90days | None |
| Liu and Cai.,2018 | China | Chinese | S | 82 | 41,41 | | 32,50 | T:24-56(34.65±6.98)y C:22-53(32.99±6.47)y | MM/TCM | T:8-25(14.24±4.66) m C:9-28(16.01±5.23) m | Bupiwei Xieyinhuo Shengyang Decoction | 4weeks | None |
| Li et al.,2018 | China | Chinese | S | 60 | 30,30 | | 35,25 | T:21-67(42.65±8.42)y C:22-65(42.12±7.86)y | MM/TCM | T:7m-4y (2.26±0.67) y C:6m-4y (2.12±0.76) y | Yiqi Yangxue Bupi Hegan Decoction | 15-30days | None |
| Du, 2018 | China | Chinese | S | 108* | 54,54 | | 52,57 | T:24-70(40.59±5.60)y C:22-65(42.12±7.86)y | MM/TCM | T:0.5-3(2.08±0.57) y C:0.5-3(2.10±0.54) y | Self-made Yishen Buxue Ointment | 6weeks | None |
| Zheng et al.,2017 | China | Chinese | S | 90 | 45,45 | | 39,51 | T:24-54(35.8±7.6)y C:22-53(34.9±8.1)y | MM/TCM | T:7-46(15.4±3.8) m C:8-49(16.2±3.5) m | Shugan Jianpi Yishen Decoction | 8weeks | 6months |
| Wei et al.,2017 | China | Chinese | S | 60 | 30,30 | | 60,0 | T:35-75(56.33±9.92)y C:36-74(55.27±10.72)y | MM/TCM | ≥6m | Long Gao | 3months | None |
| Wang, 2017 | China | Chinese | S | 140 | 70,70 | | 77,63 | T:18-68(42.47±12.46)y C:19-69(42.33±17.40)y | MM/TCM | T:7-25(12.63±4.11) m C:6-24(12.78±4.24) m | Bupi Yishen Decoction | 1month | N/A |
| Li, 2017 | China | Chinese | S | 60 | 30,30 | | 29,31 | T:24-42(34.5±4.1)y C:27-43(35.5±3.6)y | TCM | N/A | Modified Huangqi Jianzhong Decoction | 60days | None |
| Huang et al.,2017 | China | Chinese | S | 150^#8^ | 50,50,50 | | 60,90 | T:21-57y C:21-57y H:20-55y | MM | T:: ≥6m C:: ≥6m H:0-6m | Wenzhen Yunqi Formula | 12weeks | None |
| Yang and Liang.,2016 | China | Chinese | S | 60 | 30,30 | | 29,31 | T:27-43(35.6±3.7)y C:24-42(34.6±4.2)y | MM/TCM | T:6m-4y (2.2±0.8) y C:6m-5y (2.3±0.7) y | Modified Huangqi Jianzhong Decoction | 60days | None |
| Wu et al.,2016 | China | Chinese | S | 80 | 42,38 | | 53,27 | T:23-62(40.15±8.51)y C:22-60(41.46±7.94)y | MM/TCM | T:0.5-3.16(1.32±0.67) y C:0.5-3.25(1.28±0.59) y | Xiaopi - Yin | 6weeks | None |
| Sun et al.,2016 | China | Chinese | S | 80 | 40,40 | | 80,0 | T:24-55(36.58±5.48)y C:25-56(36.87±6.58)y | MM/TCM | T:10-36(18.56±6.45) m C:10-36(17.75±5.92) m | Shugan Yiyang Capsule | N/A | None |
| Huang et al.,2016 | China | Chinese | S | 150^#9^ | 50,50,50 | | 57,93 | T:(39.52±2.10)y C:(40.59±1.84)y H:(39.30±1.85)y | MM/TCM | T: ≥6m C: ≥6m H:0-6m | Wenzhen Yunqi Formula | 3months | None |
| Gao and Pang, 2016 | China | Chinese | S | 70 | 35,35 | | 27,43 | T:(32.8±10.5)y C:(33.6±12.7)y | MM/TCM | T:9-48m C:8-50m | Wendan Decoction Combined with Sini powder | 4weeks | None |
| Wang et al.,2015 | China | Chinese | S | 120 | 60,60 | | 56,64 | T:(46.72±12.56)y C:(46.02±12.94)y | MM/TCM | ≥6m | Yiqi Jianpi Bushen Decoction | 4-8weeks | None |
| Liu et al.,2015a | China | Chinese | S | 100 | 51,49 | | 39,61 | T:(43.2±12.6)y C:(42.6±10.5)y | MM/TCM | T:6m-3y C:10m-3y | Shugan Yangxue Method | 6weeks | None |
| Liu et al.,2015b | China | Chinese | S | 120 | 60,60 | | 48,72 | 15-44y | MM/TCM | ≥6m | Jianpi Jieyu Xiaopi Paste | 90days | None |
| Li, 2015 | China | Chinese | S | 68 | 34,34 | | 37,31 | T:24-74(41.3±2.3)y C:25-73(42.3±2.4)y | MM | T:0.5-4y (2.4±1.1) y C:0.5-5y (2.5±1.2) y | Buzhong Yiqi Decotion Combine with Xiaochaihu Decotion | 28days | None |
| Li and Cao.,2015 | China | Chinese | S | 74 | 37,37 | | 0,74 | T:45-58(55.3±6.2)y C:45-58(54.7±6.9)y | MM/TCM | ≥6m | Invigorating spleen warming kidney and smoothing liver Decoction | 1month | 1year |
| Guo and Guo.,2015 | China | Chinese | S | 90 | 45,45 | | 39,51 | T:20-60(38.27±6.54)y C:20-63(39.20±6.85)y | MM/TCM | T:6-35(18.26±5.54) m C:7-36(17.77±6.23) m | Danzhi Xiaoyao tablet | N/A | None |
| Gao and Pang,2015 | China | Chinese | S | 70 | 35,35 | | 27,43 | T:(32.8±10.5)y C:(33.6±12.7)y | MM/TCM | T:9-48m C:8-50m | Shugan Jianpi Method | 4weeks | None |
| Wang and Liu,2014 | China | Chinese | S | 70 | 35,35 | | 33,37 | T:21-43(36.43士5.62)y C:22-42(36.58士4.89)y | MM/TCM | T:6m-5y C:7m-5y | Jianpi Jieyu Xiaopi Paste | 3months | None |
| Teng et al.,2014 | China | Chinese | S | 60 | 30,30 | | 23,37 | T:20-64(mean=43)y C:20-65(mean=43)y | MM/TCM | T:0.5-20(mean=2.4) y C:0.5-20(mean=2.7) y | Buzhong Jiepi Decoction | 8weeks | 6month |
| Niu et al.,2014 | China | Chinese | S | 132 | 66,66 | | 48,84 | T:(44.18±8.66)y C:(46.34±9.39)y | MM/TCM | T:(10.78±4.15) m C:(11.46±3.23) m | Bushen Shugan Decoction | 8weeks | None |
| Liu et al.,2014 | China | Chinese | S | 80 | 40,40 | | 31,49 | T:23-42(35.7±6.5)y C:24-43(36.5±5.9)y | MM/TCM | T:6m-4y (2.3±0.8) y C:6m-3.8y (2.5±0.7) y | Jianpi Jieyu Xiaopi Paste | 3months | None |
| Dai et al.,2014 | China | Chinese | N/A | 80 | 40,40 | | N/A | 18-60y | MM | 6m-3y | Buyi Pishen Decoction | 30-40days | None |
| Zhao et al.,2013 | China | Chinese | S | 87^#10^ | 44,43 | | 0,87 | T:17-58(mean=36.5)y C:16-57(mean=35.6)y | MM/TCM | T:7-29(9.35±2.13) m C:7-26(9.05±3.13) m | Fufangteng Mixture | 3months | after 6months |
| Xu et al.,2013 | China | Chinese | S | 68 | 40,28 | | 39,30 | T:15-65(33.24±1.56)y C:13-60(30.24±1.28)y | MM | T:6m-6y (24.24±4.30) m C:6m-5y (22.20±3.24) m | Modified Naoxinkang | 10days | None |
| Xu and Wang.,2013 | China | Chinese | S | 84 | 42,42 | | 36,48 | T:18-58(35.29±6.18)y C:20-60(34.87±7.08)y | MM/TCM | T:9-35(15.06±4.80) m C:10-33(14.75±5.02) m | Chaihu Combine with Longgu Muli Decoction | N/A | None |
| Sun, 2013 | China | Chinese | S | 30 | 15,15 | | 18,12 | 20-51(mean=43.2)y | MM | 9m-3y (mean=1.7y) | Panax quinquefolius L.prepared Slices | 28days | None |
| Kim et al., 2013 | Korea | English | S | 90 | 30,29,29 | | 21,69 | T(H):22-59(mean=40.5)y T(L):25-57(mean=39.5)y C:24-60(mean=39.5)y | MM | T1:(19.9±12.5) m T2:(20.8±20.2) m C:(17.6±12.4) m | Extract of P. ginseng | 4weeks | None |

S: single-center; M: Multi-center; T: test group; C: control group; H: health group; H: high dose; L: low dose; MM: Modern medicine; TCM: traditional Chinese medicine

#1: In the control group, 2 participants dropped out during treatment; #2: In the control group, 2 participants dropped out during treatment; #3: there were 2 drop-outs in the treatment group and 2 drop-outs in the control group; #4 Fifteen subjects dropped out, leaving 175 subjects in the (PP) analysis; #5: Eight drop-outs (4 drop-outs due to fail to return in time and one drop-outs after the course of treatment was interrupted in the treatment group; One patient could not be contacted after completing the trial. The 3 drop-outs in the control group failed to receive medicine in time during the course of treatment, and the treatment was interrupted and dropped off.);

#6: Three participants dropped out due to treatment discontinuation after visit 2 (1 in the KRG group and 2 in the placebo group); #7 One participant dropped out due to personal reasons before starting the first drug administration and was excluded from the ITT analysis; #8: No specific cause was found in 3 drop-outs; #9: During the course of treatment, one participants dropped out in the treatment group and 2 participants in the control group; #10: During the course of treatment, one participants dropped out in the treatment group and 2 participants in the control group.

| Supplementary Table 9. Intervention of the RCTs included in the review | | | | | | | | | | | | | |
| --- | --- | --- | --- | --- | --- | --- | --- | --- | --- | --- | --- | --- | --- |
| Reference | **Test group** | | **Dosage form** | **Ingredients** | | | **Dose (T)** | **Control group** | | | | | **Dose (C)** |
| Zhou et al.,2022 | | Xinshen’an Capsule | Capsule | *Panax ginseng C. A. Mey. ,Schisandra chinensis（Turcz.）Baill.,Ophiopogon japonicus (L. f.) Ker-Gawl.,Salvia miltiorrhiza Bge.，Astragalus membranaceus（Fisch.） Bge.,Atractylodes macrocephala Koidz.,Dimocarpus longan Lour.,Polygala tenuifolia Willd. ,Aucklandia lappa Decne.，Poria cocos（Schw.）Wolf,Ligusticum chuanxiong Hort.,Angelica sinensis（Oliv.）Diels,Ziziphus jujuba Mill. var. spinosa（Bunge）Hu ex H. F. Chou，Gynostemma pentaphyllum （Thunb.）Mak.,Acorus tatarinowii Schott,Corydalis yanhusuo W.T.Wang* | | | 3 capsules/ time, 3 times/day | Renshen Guipi pill | | | | | 1 pill /time, twice /day |
| Wu et al.,2022 | | Dispelling Dampness-Replenishing Qi-Nourishing Yin Step Therapy | Decoction | *Ⅰ:Coix lacryma-jobi L.var.ma-yuen(Roman.) Stapf,Hordeum vulgare L.,Setaria italica （L. ） Beauv. ,TALCUM,Benincasa hispida(Thunb.) Cogn.,Prunus armeniaca L. ,Magnolia officinalis Rehd.et Wils.,Pinellia ternata（Thunb.） Breit.,Lophatherum gracile Brongn. ,Dioscorea spongiosa J. Q. Xi，M. Mizuno et W. L. Zhao,Cannabis sativa L.,Areca catechu L.,Amomum villosum Lour.,Alisma plantago-aquatica Linn.,Glycyrrhiza uralensis Fisch.* | | | 300ml / time, once/day | Ganmai Dazao decoction | | | | | 150ml / time, twice /day |
|  |  |  |  | *Ⅱ:Astragalus membranaceus（Fisch.） Bge. ,Trichosanthes kirilowii Maxim. , Angelica sinensis（Oliv.）Diels ,Pseudostellaria heterophylla（Miq.）Pax ex Pax et Hoffm.,Areca catechu L.,Cannabis sativa L.,Citrus grandis (L.)Osbeck,Bambusa tuldoides Munro,Fritillaria thunbergii Miq. ,Citrus reticulata Blanco ,Atractylodes macrocephala Koidz.,Bupleurum chinense DC. or Bupleurum scorzonerifolium Willd. ,Glycyrrhiza uralensis Fisch. , Cimicifuga heracleifolia Kom.* | | |  |  |  |  |  |  |  |
|  |  |  |  | *Ⅲ:Astragalus membranaceus（Fisch.） Bge.,Angelica sinensis（Oliv.）Diels,Dolichos lablab L.,Pseudostellaria heterophylla（Miq.）Pax ex Pax et Hoffm.,Adenophora stricta Miq.,Polygonatum odoratum（Mill.）Druce,Ophiopogon japonicus (L. f.) Ker-Gawl.,Trichosanthes kirilowii Maxim.,Dendrobium nobile Lindl.,Citrus reticulata Blanco ,Atractylodes macrocephala Koidz.,Morus alba L.,Bupleurum chinense DC. or Bupleurum scorzonerifolium Willd.,生Glycyrrhiza uralensis Fisch.,Cimicifuga heracleifolia Kom.* | | |  |  |  |  |  |  |  |
| Ma et al.,2022 | | Dispelling Dampness-Replenishing Qi-Nourishing Yin Step Therapy | Decoction | *Ⅰ:Coix lacryma-jobi L.var.ma-yuen(Roman.) Stapf,Hordeum vulgare L.,Setaria italica （L. ） Beauv. ,TALCUM,Benincasa hispida(Thunb.) Cogn.,Prunus armeniaca L. ,Magnolia officinalis Rehd.et Wils.,Pinellia ternata（Thunb.） Breit.,Lophatherum gracile Brongn. ,Dioscorea spongiosa J. Q. Xi，M. Mizuno et W. L. Zhao,Cannabis sativa L.,Areca catechu L.,Amomum villosum Lour.,Alisma plantago-aquatica Linn.,Glycyrrhiza uralensis Fisch.* | | | 300ml / time, once/day | Ganmai Dazao decoction | | | | | 150ml / time, twice/day |
|  |  |  |  | *Ⅱ:Astragalus membranaceus（Fisch.） Bge. ,Trichosanthes kirilowii Maxim. , Angelica sinensis（Oliv.）Diels ,Pseudostellaria heterophylla（Miq.）Pax ex Pax et Hoffm.,Areca catechu L.,Cannabis sativa L.,Citrus grandis (L.)Osbeck,Bambusa tuldoides Munro,Fritillaria thunbergii Miq. ,Citrus reticulata Blanco ,Atractylodes macrocephala Koidz.,Bupleurum chinense DC. or Bupleurum scorzonerifolium Willd. ,Glycyrrhiza uralensis Fisch. , Cimicifuga heracleifolia Kom.* | | |  |  |  |  |  |  |  |
|  |  |  |  | *Ⅲ:Astragalus membranaceus（Fisch.） Bge.,Angelica sinensis（Oliv.）Diels,Dolichos lablab L.,Pseudostellaria heterophylla（Miq.）Pax ex Pax et Hoffm.,Adenophora stricta Miq.,Polygonatum odoratum（Mill.）Druce,Ophiopogon japonicus (L. f.) Ker-Gawl.,Trichosanthes kirilowii Maxim.,Dendrobium nobile Lindl.,Citrus reticulata Blanco ,Atractylodes macrocephala Koidz.,Morus alba L.,Bupleurum chinense DC. or Bupleurum scorzonerifolium Willd.,生Glycyrrhiza uralensis Fisch.,Cimicifuga heracleifolia Kom.* | | |  |  |  |  |  |  |  |
| Li et al.,2022 | | Guashen Decoction+healthy lifestyle | Decoction | *Panax ginseng C. A. Mey. ,Trichosanthes kirilowii Maxim.,Astragalus membranaceus（Fisch.） Bge.,Dendrobium nobile Lindl.,Atractylodes macrocephala Koidz.,Scrophularia ningpoensis Hemsl.,Ophiopogon japonicus (L. f.) Ker-Gawl.,Rehmannia glutinosa Libosch., Dianthus superbus L. or Dianthus chinensis L. ,Plantago asiatica L. or Plantago depressa Willd.,Coptis chinensis Franch.（or Coptis deltoidea C.Y.Cheng et Hsiao,or Coptis teeta Wall.,Leonurus japonicus Houtt.,Achyranthes bidentata Bl.* | | | 1 pack / time, twice/day | Shengmai Decoction+healthy lifestyle | | | | | 150ml / time, twice/day |
| Huang et al.,2022 | | Wenzhen Yunqi Formula+Conventional Western medicine+healthy lifestyle | Decoction | *Astragalus membranaceus（Fisch.） Bge.,Codonopsis pilosula (Franch.)Nannf.,Epimedium brevicornu Maxim.,Acorus tatarinowii Schott,Curcuma wenyujin Y. H. Chen et C. Ling（or Curcuma Longa L. or Curcuma kwangsiensis S. G. Lee et C. F. Liang or Curcuma phaeocaulis Val.) ,Salvia miltiorrhiza Bge.,Actinolite,Sedum aizoon L.or.Sedum kamtschaticum Fisch.* | | | 1 pack (150ml) / time, twice/day | Conventional Western medicine+healthy lifestyle | | | | | N/A |
| Guo and Huang,2022 | | Fali Decoction+Paroxetine hydrochloride | Decoction | *Rehmannia glutinosa Libosch.,Adenophora stricta Miq.,Achyranthes bidentata Bl.,Saposhnikovia divaricata （Turcz.）Schischk. ,Alisma plantago-aquatica Linn.,Polygala tenuifolia Willd. ,Epimedium brevicornu Maxim.,Astragalus membranaceus（Fisch.） Bge.,Paeonia lactiflora Pall.,Atractylodes macrocephala Koidz.,Poria cocos（Schw.）Wolf,Codonopsis pilosula (Franch.)Nannf.* | | | 1 pack / time, twice/day | Paxil hydrochloride | | | | | 20-40mg / time, once/day |
| Liu et al.,2021 | | Chaihu Guizhi Decoction | Granules | *Bupleurum chinense DC. or Bupleurum scorzonerifolium Willd.,Cinnamomum cassia Presl,Codonopsis pilosula (Franch.)Nannf.,Scutellaria baicalensis Georgi,Pinellia ternata（Thunb.） Breit.,Paeonia lactiflora Pall.,Glycyrrhiza uralensis Fisch. ,Zingiber officinale Rosc,Ziziphus jujuba Mill.* | | | 1 pack (12g) / time, twice/day | placebo | | | | | 1 pack (12g)/ time, twice/day |
| Chen ,2021 | | Xiaoyao powder | Decoction | *Poria cocos（Schw.）Wolf,Paeonia lactiflora Pall.,Atractylodes macrocephala Koidz.，Angelica sinensis（Oliv.）Diels，Glycyrrhiza uralensis Fisch.,Bupleurum chinense DC. or Bupleurum scorzonerifolium Willd.，Mentha haplocalyx Briq. ，Zingiber officinale Rosc* | | | 1 pack / time, twice/day | ATP + Oryzanol Tablets + VitaminB1 | | | | | 20mg*3 / time, 3 times/day |
| Shin et al., 2021 | | Sipjeondaebo-tang | Granules | *Panax ginseng C. A. Mey. ，Astragalus membranaceus（Fisch.） Bge.，Poria cocos（Schw.）Wolf，Atractylodes macrocephala Koidz.(or Atractylodes lancea（Thunb.）DC. Koidzumi)，Angelica dahurica（Fisch.ex Hoffm.）Benth.et Hook.f.(or Angelica gigas Nakai)，Paeonia lactiflora Pall.，Ligusticum chuanxiong Hort.，Cinnamomum cassia Presl，Rehmannia glutinosa Libosch.and Glycyrrhiza uralensis Fisch.(or glycyrrza glabra Linné，or Glycyrrhiza inflata Batal)* | | | 1 pack (3g)/ time, 3 times/day | placebo | | | | | 1 pack (3g)/ time, 3 times/day |
| Kan et al., 2021; | | Cistanche and Ginkgo extracts | Tablet | *Cistanche tubulosa &Ginkgo biloba leaf* | | T(H):one tablet (450 mg*Cistanche tubulosa+ 180 mg* Ginkgo biloba leaf)/time, once/day | | | | | | placebo | one tablet/time, once/day |
|  |  |  |  |  |  | T(L):one tablet (300 mg*Cistanche tubulosa+ 120 mg* Ginkgo biloba leaf)/time, once/day | | | | | |  |  |
| Zhang , 2020 | | Buzhong Yiqi Decotion Combine with Xiaochaihu Decotion | Decoction | *Codonopsis pilosula (Franch.)Nannf.,Astragalus membranaceus（Fisch.） Bge.,Bupleurum chinense DC. or Bupleurum scorzonerifolium Willd.,Citrus reticulata Blanco ,Scutellaria baicalensis Georgi,Pinellia ternata（Thunb.） Breit.，Agrimonia pilosa Ledeb.，Poria cocos（Schw.）Wolf,Atractylodes macrocephala Koidz.,Curcuma wenyujin Y. H. Chen et C. Ling（or Curcuma Longa L. or Curcuma kwangsiensis S. G. Lee et C. F. Liang or Curcuma phaeocaulis Val.) ，Platycodon grandiflorum （Jacq.）A.DC.* | | | 1 pack / time, twice/day | | | | Modified Xiaoyao pills | | 1 pack (6g)/ time, twice/day |
| Sheng , 2020 | | Chaihu Guizhi Decoction | Granules | *Bupleurum chinense DC. or Bupleurum scorzonerifolium Willd.,Codonopsis pilosula (Franch.)Nannf.,Scutellaria baicalensis Georgi,Paeonia lactiflora Pall.,Glycyrrhiza uralensis Fisch.,Cinnamomum cassia Presl,Pinellia ternata（Thunb.） Breit.,Ziziphus jujuba Mill.及Zingiber officinale Rosc* | | | 1 pack(12g) / time, twice/day | Xiaoyao pills | | | | | 8 pills /time,3 times / day. |
| Mao , 2020 | | Yishen Tiaodu Method | Decoction | *Dioscorea opposita Thunb.,Oryza sativa L.,Euryale ferox Salisb. ,Allium tuberosum RottL.ex Spreng.* | | | 1 pack(400-500ml) / time,once/every 2 days | Oryzanol Tablets | | | | | 20mg / time, 3 times/day |
| Li , 2020 | | Buzhong Yiqi Decotion Combine withXiaochaihu Decotion | Decoction | *Bupleurum chinense DC. or Bupleurum scorzonerifolium Willd.,Atractylodes macrocephala Koidz.,Poria cocos（Schw.）Wolf,Bupleurum chinense DC. or Bupleurum scorzonerifolium Willd.,Codonopsis pilosula (Franch.)Nannf.，Curcuma wenyujin Y. H. Chen et C. Ling（or Curcuma Longa L. or Curcuma kwangsiensis S. G. Lee et C. F. Liang or Curcuma phaeocaulis Val.) ，Glycyrrhiza uralensis Fisch.Ziziphus jujuba Mill.* | | | N/A | ATP | | | | | 40mg / time, 3 times/day |
| Huang et al.,2020 | | Yangwei Jianpi Plaster | Plaster | *Codonopsis pilosula (Franch.)Nannf.,Dioscorea opposita Thunb.,Atractylodes macrocephala Koidz.,Poria cocos（Schw.）Wolf,Glycyrrhiza uralensis Fisch.,Nelumbo nucifera Gaertn.,Coix lacryma-jobi L.var.ma-yuen(Roman.) Stapf,Platycodon grandiflorum （Jacq.）A.DC.,Dolichos lablab L.,Astragalus membranaceus（Fisch.） Bge.,Cinnamomum cassia Presl,Paeonia lactiflora Pall.,Zingiber officinale Rosc,Ziziphus jujuba Mill.,Saposhnikovia divaricata （Turcz.）Schischk. ,Gallus gallus domesticus Brisson ,Massa Medicata Fermentata* | | | 1 tablespoon(20g)/time, once/day | Buzhong Yiqi Decotion | | | | | 200ml / time, twice/day |
| Dong , 2020 | | Qingshu Yiqi Decotion | Decoction | *Glycyrrhiza uralensis Fisch.，Cimicifuga heracleifolia Kom.,Schisandra chinensis（Turcz.）Baill.９ｇ，Amomum villosum Lour.,Citrus reticulata Blanco，Phellodendron chinense Schneid.,Citrus reticulata Blanco，Atractylodes lancea（Thunb.）DC. ,Poria cocos（Schw.）Wolf,Alisma plantago-aquatica Linn.,Angelica sinensis（Oliv.）Diels,Codonopsis pilosula (Franch.)Nannf.,Atractylodes macrocephala Koidz.,Mosla chinensis Maxim. or Mosla chinensis‘Jiangxiangru’，Massa Medicata Fermentata，Astragalus membranaceus（Fisch.） Bge.* | | | 200ml / time, twice/day | Nordicon capsules | | | | | 2 capsules/time, 3 times / day |
| Sung et al., 2020 | | Korean red ginseng (KRG) | Capsule | *Panax ginseng C.A.Mey.* | | | 3 capsules(0.5g)/time, twice/day | placebo | | | | | 3 capsules(0.5g)/time, twice/day |
| Yang, 2019 | | Zuogui Pills | Pill | *Rehmannia glutinosa Libosch.,Dioscorea opposita Thunb.,Cornus officinalis Sieb. et Zucc.,Lycium barbarum L.,CERVI CORNUS COLLA,Cuscuta australis R.Br.,Eucommia ulmoides Oliv.,Angelica sinensis（Oliv.）Diels,Cinnamomum cassia Presl,Aconitum carmichaelii Debx.* | | | 9g / time, twice/day | Conventional Western medicine | | | | | N/A |
| Wang, 2019 | | Buzhong Yiqi Decotion Combine with Xiaochaihu Decotion | Decoction | *Astragalus membranaceus（Fisch.） Bge. ,Codonopsis pilosula (Franch.)Nannf. ,Agrimonia pilosa Ledeb.,Atractylodes macrocephala Koidz. ,Curcuma wenyujin Y. H. Chen et C. Ling（or Curcuma Longa L. or Curcuma kwangsiensis S. G. Lee et C. F. Liang or Curcuma phaeocaulis Val.) ,Poria cocos（Schw.）Wolf ,Bupleurum chinense DC. or Bupleurum scorzonerifolium Willd.,Pinellia ternata（Thunb.） Breit. ,Citrus reticulata Blanco ,Ziziphus jujuba Mill.,Glycyrrhiza uralensis Fisch.,Scutellaria baicalensis Georgi ,Platycodon grandiflorum （Jacq.）A.DC.* | | | 1 pack / time, twice/day | Modified Xiaoyao pills | | | | | 1 pack(6g)/ time, twice/day |
| Shi, 2019 | | Modified Xiaoyao San + Psychological counseling | Decoction | *Angelica sinensis（Oliv.）Diels Paeonia lactiflora Pall. ,Bupleurum chinense DC. or Bupleurum scorzonerifolium Willd. ,Poria cocos（Schw.）Wolf ,Atractylodes macrocephala Koidz. ,Glycyrrhiza uralensis Fisch. , Mentha haplocalyx Briq. ,Codonopsis pilosula (Franch.)Nannf. ,Dioscorea opposita Thunb.* | | | 150ml / time, twice/day | Oryzanol Tablets + Psychological counseling | | | | | 20mg / time, 3 times/day |
| Ma et al.,2019 | | Modified Erxian Decoction | Decoction | *Epimedium brevicornu Maxim. ，Curculigo orchioides Gaertn. ，Morinda officinalis How ，Astragalus membranaceus（Fisch.） Bge. ，Codonopsis pilosula (Franch.)Nannf.， Angelica sinensis（Oliv.）Diels ，Cimicifuga heracleifolia Kom. ，Bupleurum chinense DC. or Bupleurum scorzonerifolium Willd. ，Phellodendron chinense Schneid. ，Anemarrhena asphodeloides Bge.，* | | | 120ml / time, twice/day | Vitamin B1+Oryzanol Tablets+Baile Mian Capsule | | | | | 20mg*(VB1+Oryzanol )/ time and 4 capsule Bale Mian , 3 times/day |
| Liu et al.,2019a | | Jianpi Yishen Decoction | Decoction | *Cuscuta australis R.Br. or Cuscuta chinensis Lam. ，Lycium barbarum L. ，Pseudostellaria heterophylla（Miq.）Pax ex Pax et Hoffm. ，Atractylodes macrocephala Koidz. ， Poria cocos（Schw.）Wolf ，Dioscorea opposita Thunb. ，Citrus reticulata Blanco ，Cistanche deserticola Y.C.Ma ，Psoralea corylifolia L. ，Poria cocos（Schw.）Wolf ，Polygala tenuifolia Willd. ，Glycyrrhiza uralensis Fisch.* | | | 1 dose / time, twice/day | VitaminC+VitaminB+VitaminE | | | | | 1dose(VC*0.1g+VB*0.2g+VE*0.1g)/time, 3 times/day |
| Liu et al.,2019b | | Chaihu Guizhi Decoction | Granules | *Bupleurum chinense DC. or Bupleurum scorzonerifolium Willd.，Cinnamomum cassia Presl,Codonopsis pilosula (Franch.)Nannf.,Scutellaria baicalensis Georgi,Pinellia ternata（Thunb.） Breit.,Paeonia lactiflora Pall.,Glycyrrhiza uralensis Fisch.，Zingiber officinale Rosc，Ziziphus jujuba Mill.* | | | 1 pack / time, twice / day | placebo | | | | | 1 pack / time, twice / day |
| Liu et al.,2019c | | Chaihu Guizhi Decoction | Granules | *Bupleurum chinense DC. or Bupleurum scorzonerifolium Willd.,Cinnamomum cassia Presl,Codonopsis pilosula (Franch.)Nannf.,Scutellaria baicalensis Georgi,Pinellia ternata（Thunb.） Breit.,Paeonia lactiflora Pall.,Glycyrrhiza uralensis Fisch. ,Zingiber officinale Rosc,Ziziphus jujuba Mill.* | | | 1 pack (12g)/ time, twice/day | placebo | | | | | 1 pack (12g)/ time, twice/day |
| Liu et al.,2019d | | Modified Lingzhi Pills | Pill | *Polygonum multiflorum Thunb. ，TESTUDINIS CARAPACIS ET PLASTRI COLLA ，Ganoderma lucidum（Leyss.ex Fr.）Karst. ，Astragalus membranaceus（Fisch.） Bge.，Panax notoginseng （ Burk.） F. H. ，Acorus tatarinowii Schott，Polygala tenuifolia Willd.* | | | 1 pack / time, 3 times / day | Fluoxetine tablets | | | | | 20mg / time, 3 times/day |
| Lin et al.,2019 | | Self made fatigue Decoction | Decoction | *Codonopsis pilosula (Franch.)Nannf. ，Poria cocos（Schw.）Wolf，Atractylodes macrocephala Koidz.，Paeonia lactiflora Pall.，Astragalus membranaceus（Fisch.） Bge. ， Epimedium brevicornu Maxim.，Polygala tenuifolia Willd. ，Alisma plantago-aquatica Linn.，Saposhnikovia divaricata （Turcz.）Schischk. ，Achyranthes bidentata Bl.， Adenophora stricta Miq.，Rehmannia glutinosa Libosch.* | | | 150ml / time, 3 times/day | Jinri Yangshen pills | | | | | 2 capsules(0.5g) / time, twice/day |
| Li et al.,2019 | | Dalishen Tea | Drinks | *Polyrhachis dives,Panax quinquefolium L.,Lepidium meyenii Walp.,Cornus officinalis Sieb. et Zucc.,Lycium barbarum L.,Rubus chingii Hu ,Nelumbo nucifera Gaertn.,Euryale ferox Salisb.* | | | 1 pack / time, 3 times / day | Oryzanol Tablets tablets | | | | | 20mg / time, 3 times/day |
| Hu, 2019 | | Buzhong Yiqi Decotion Combine with Xiaochaihu Decotion | Decoction | *Codonopsis pilosula (Franch.)Nannf.,Astragalus membranaceus（Fisch.） Bge.,Bupleurum chinense DC. or Bupleurum scorzonerifolium Willd.,Agrimonia pilosa Ledeb.,Atractylodes macrocephala Koidz.,Pinellia ternata（Thunb.） Breit.,Poria cocos（Schw.）Wolf,Curcuma wenyujin Y. H. Chen et C. Ling（or Curcuma Longa L. or Curcuma kwangsiensis S. G. Lee et C. F. Liang or Curcuma phaeocaulis Val.) ,Platycodon grandiflorum （Jacq.）A.DC.,Citrus reticulata Blanco ,Scutellaria baicalensis Georgi* | | | 1 pack / time, twice/day | ATP | | | | | 2 tablets /time, 3 times/day |
| Ding, 2019 | | Guipi Decoction | Decoction | *Astragalus membranaceus（Fisch.） Bge. ，Ziziphus jujuba Mill. var. spinosa（Bunge）Hu ex H. F. Chou ，Poria cocos（Schw.）Wolf,Dimocarpus longan Lour.,Polygala tenuifolia Willd. ,Angelica sinensis（Oliv.）Diels,Atractylodes macrocephala Koidz., Codonopsis pilosula (Franch.)Nannf. or Codonopsis pilosula Nannf.var.modesta（Nannf.）L.T.Shen or Codonopsis tangshen Oli ，Aucklandia lappa Decne.，；Glycyrrhiza uralensis Fisch.，；* | | | 200ml / time, twice/day | conventional therapy(Vitamin B and Vitamin C+jogging) | | | | | N/A |
| Joung et al., 2019 | | Myelophil | capsule | *Astragalus membranaceus（Fisch.） Bge., Salvia miltiorrhiza Bge.* | | | a dose(2g) /time , once/day | placebo | | | | | a dose(2g) /time , once/day |
| Wu et al.,2018 | | Guipi Decoction | Decoction | *Codonopsis pilosula (Franch.)Nannf. ，Astragalus membranaceus（Fisch.） Bge.，Dimocarpus longan Lour.，Ziziphus jujuba Mill. var. spinosa（Bunge）Hu ex H. F. Chou ，Poria cocos（Schw.）Wolf，Atractylodes macrocephala Koidz. ，Angelica sinensis（Oliv.）Diels ，Ligusticum chuanxiong Hort. ，Paeonia lactiflora Pall. ，Bupleurum chinense DC. or Bupleurum scorzonerifolium Willd. ，Citrus aurantium L. ，Curcuma wenyujin Y. H. Chen et C. Ling（or Curcuma Longa L. or Curcuma kwangsiensis S. G. Lee et C. F. Liang or Curcuma phaeocaulis Val.) ，Aucklandia lappa Decne. ，Polygala tenuifolia Willd. or Polygala sibirica L. ，Citrus reticulata Blanco ，Cimicifuga heracleifolia Kom. ，Glycyrrhiza uralensis Fisch.* | | | 200ml / time, twice/day | conventional therapy(Vitamin B and Vitamin C+jogging) | | | | | N/A |
| Ou et al.,2018 | | Guipi Decoction | Decoction | *Astragalus membranaceus（Fisch.） Bge.，Ziziphus jujuba Mill. var. spinosa（Bunge）Hu ex H. F. Chou，Codonopsis pilosula (Franch.)Nannf.,Poria cocos（Schw.）Wolf,Dimocarpus longan Lour.,Atractylodes macrocephala Koidz.,Polygala tenuifolia Willd. ,Angelica sinensis（Oliv.）Diels，Glycyrrhiza uralensis Fisch.，Aucklandia lappa Decne.* | | | 150ml / time, twice/day | Fluoxetine hydrochloride capsules | | | | | 20-40 mg/ time, once/every other day |
| Liu and Cai.,2018 | | Bupiwei Xieyinhuo Shengyang Decoction + Fluoxetine hydrochloride capsules | Decoction | *Bupleurum chinense DC. or Bupleurum scorzonerifolium Willd.，Astragalus membranaceus（Fisch.） Bge.,Atractylodes lancea（Thunb.）DC. ,Notopterygium incisum Ting ex H. T. Chang ,Glycyrrhiza uralensis Fisch.，Cimicifuga heracleifolia Kom.，Panax ginseng C. A. Mey. ,Scutellaria baicalensis Georgi，Coptis chinensis Franch.（or Coptis deltoidea C.Y.Cheng et Hsiao,or Coptis teeta Wall.，GYPSUM FIBROSUM* | | | 200ml / time, twice/day | Fluoxetine hydrochloride capsules | | | | | 20 mg/ time, once/every other day |
| Li et al.,2018 | | Yiqi Yangxue Bupi Hegan Decoction | Decoction | *Astragalus membranaceus（Fisch.） Bge., Atractylodes macrocephala Koidz. , Paeonia lactiflora Pall. , Poria cocos（Schw.）Wolf , Dioscorea opposita Thunb. , Panax ginseng C. A. Mey. , Rehmannia glutinosa Libosch. , Angelica sinensis（Oliv.）Diels , Ligusticum chuanxiong Hort. , Bupleurum chinense DC. or Bupleurum scorzonerifolium Willd. , Cyperus rotundus L. , Corydalis yanhusuo W.T.Wang , Poria cocos（Schw.）Wolf , Gardenia jasminoides Ellis , Glycyrrhiza uralensis Fisch.* | | | 300ml / time, twice/day | Paxil hydrochloride | | | | | 20-40 mg/ time, once/day |
| Du, 2018 | | Self-made Yishen Buxue Ointment | Plaster | *Angelica sinensis（Oliv.）Diels ，Rehmannia glutinosa Libosch.，Paeonia lactiflora Pall. ，Ligusticum chuanxiong Hort. ，Cuscuta australis R.Br.，Epimedium brevicornu Maxim. ，Psoralea corylifolia L.，Lycium barbarum L.* | | | 1 pack (150-200ml) / time, twice/day | VitaminC+MultivitaminB +Oryzanol +Adenosine Triphosphate | | | | | 1 dose(VC*0. 1 g +MultiVB*0.2g +Oryzanol * 20 mg +ATP*20 mg)/time, 3 times/day |
| Zheng et al.,2017 | | Shugan Jianpi Yishen Decoction+Paxil hydrochloride | Decoction | *Astragalus membranaceus（Fisch.） Bge.，Codonopsis pilosula (Franch.)Nannf.,Atractylodes macrocephala Koidz.，Anemarrhena asphodeloides Bge.,Citrus reticulata Blanco ,Bupleurum chinense DC. or Bupleurum scorzonerifolium Willd.,Cimicifuga heracleifolia Kom.,Paeonia lactiflora Pall.,Cuscuta australis R.Br.,Epimedium brevicornu Maxim.,Lycium barbarum L.，Glycyrrhiza uralensis Fisch.* | | | 150ml / time, twice/day | Paxil hydrochloride | | | | | 20mg / time, once/day |
| Wei et al.,2017 | | Long Gao | Plaster | *CERVI CORNUS COLLA,Rehmannia glutinosa Libosch.,Rehmannia glutinosa Libosch.,Epimedium brevicornu Maxim.,Dimocarpus longan Lour.,Pueraria lobata（Willd.）Ohwi ,Siphonostegia chinensis Benth.,MEL* | | | 1 dose(15g) / time, twice/day | Liuwei Dihuang pill | | | | | 16 pills / time, twice/day |
| Wang, 2017 | | Bupi Yishen Decoction + Adenosine Triphosphate+ Oryzano | Decoction | *Panax ginseng C.A.Mey. ，Atractylodes macrocephala Koidz.，Rhodiola crenulata （Hook. f. et Thoms. ）H. Ohba，Poria cocos（Schw.）Wolf，Cuscuta australis R.Br.，Psoralea corylifolia L.，Citrus reticulata Blanco ，Cornus officinalis Sieb. et Zucc,Dioscorea opposita Thunb.，Pinellia ternata（Thunb.） Breit.,Glycyrrhiza uralensis Fisch.,Bupleurum chinense DC. or Bupleurum scorzonerifolium Willd.* | | | 1 pack (400ml) / time, twice/day | Adenosine Triphosphate+ Oryzano | | | | | 1 dose(Oryzanol*20 mg +ATP*60 mg)/time, 3 times/day |
| Li, 2017 | | Modified Huangqi Jianzhong Decoction | Decoction | *Astragalus membranaceus（Fisch.） Bge.，Codonopsis pilosula (Franch.)Nannf.，Ziziphus jujuba Mill.，Citrus aurantium L.，Aconitum carmichaelii Debx.，Cinnamomum cassia Presl，Glycyrrhiza uralensis Fisch.，Paeonia lactiflora Pall.，Atractylodes macrocephala Koidz.，Zingiber officinale Rosc.* | | | 150ml / time, 3 times/day | Shenqi pill | | | | | 1 pill / time, twice/day |
| Huang et al.,2017 | | Wenzhen Yunqi Formula | Decoction | *Astragalus membranaceus（Fisch.） Bge.,Codonopsis pilosula (Franch.)Nannf.,Epimedium brevicornu Maxim.,Acorus tatarinowii Schott,Curcuma wenyujin Y. H. Chen et C. Ling（or Curcuma Longa L. or Curcuma kwangsiensis S. G. Lee et C. F. Liang or Curcuma phaeocaulis Val.) ,Salvia miltiorrhiza Bge.,Actinolite,Sedum aizoon L. or Sedum kamtschaticum Fisch.* | | | 1 pack (150ml) / time, twice/day | C：Buzhong Yiqi pill | | | | | 8 capsules / time, 3 times/day |
|  |  |  |  |  |  |  |  | H：none | | | | |  |
| Yang and Liang.,2016 | | Modified Huangqi Jianzhong Decoction | Decoction | *Astragalus membranaceus（Fisch.） Bge. ，Codonopsis pilosula (Franch.)Nannf. Cinnamomum cassia Presl ， Paeonia lactiflora Pall. ，Glycyrrhiza uralensis Fisch. ，Ziziphus jujuba Mill. ，Aconitum carmichaelii Debx. Zingiber officinale Rosc. ，Citrus aurantium L. ，Atractylodes macrocephala Koidz.* | | | 150ml / time, 3 times/day | Guipi pill | | | | | 10 pills / time, 3 times / day |
| Wu et al.,2016 | | Xiaopi - Yin | Decoction | *Panax ginseng C.A.Mey. ，Atractylodes macrocephala Koidz. ，Poria cocos（Schw.）Wolf ，Cuscuta australis R.Br. ，Lycium barbarum L. ，Epimedium brevicornu Maxim. ，Dioscorea opposita Thunb. ，Psoralea corylifolia L. ，Angelica sinensis（Oliv.）Diels ，Alisma plantago-aquatica Linn. ，Astragalus membranaceus（Fisch.） Bge.var.mongholicus（Bge.）Hsiao or Astragalus membranaceus（Fisch.）Bge. ，Nardostachys jatamansi DC. ，Glycyrrhiza uralensis Fisch.* | | | 200ml / time, 3 times/day | Vitamin B6 | | | | | 2 tablets/time , once/day |
| Sun et al.,2016 | | Shugan Yiyang Capsule+Paxil hydrochloride | Capsule | *Bupleurum chinense DC. or Bupleurum scorzonerifolium Willd.,Tribulus terrestris L.,Aspongopus chinensis Dallas,Polistes mandarinus Saussure,Cnidium monnieri（L.）Cuss,Cistanche deserticola Y.C.Ma,Cuscuta australis R.Br.,Schisandra chinensis（Turcz.）Baill.,Morinda officinalis How,Polygala tenuifolia Willd. ,Acorus tatarinowii Schott,Pheretima aspergillum(E.Perrier）（or Pheretima vulgaris Chen or Pheretima guillelmi（Michaelsen）or Pheretima pectinifera Michaelsen）,Whitmania pigra Whitman（or Hirudo nipponica Whitman or Whitmania acranulata Whitman）,Scolopendra subspinipes mutilans L. Koch* | | | 1dose(0.75g)/time, 3 times/day | Paxil hydrochloride | | | | | 20mg / time, once/day |
| Huang et al.,2016 | | Wenzhen Yunqi Formula | Decoction | *Astragalus membranaceus（Fisch.） Bge.,Codonopsis pilosula (Franch.)Nannf.,Epimedium brevicornu Maxim.,Acorus tatarinowii Schott,Curcuma wenyujin Y. H. Chen et C. Ling（or Curcuma Longa L. or Curcuma kwangsiensis S. G. Lee et C. F. Liang or Curcuma phaeocaulis Val.) ,Salvia miltiorrhiza Bge.,Actinolite,Sedum aizoon L. or Sedum kamtschaticum Fisch.* | | | 1 pack (150ml) / time, twice/day | C：Xiaoyao pill | | | | | 8 capsules / time, 3 times/day |
|  |  |  |  |  |  |  |  | H：none | | | | |  |
| Gao and Pang, 2016 | | Wendan Decoction Combined with Sini powder + Fluoxetine hydrochloride | Decoction | *Pinellia ternata（Thunb.） Breit.,Bambusa tuldoides Munro,Glycyrrhiza uralensis Fisch.,Bupleurum chinense DC. or Bupleurum scorzonerifolium Willd.,Citrus aurantium L.,Paeonia lactiflora Pall.，Zingiber officinale Rosc，Citrus reticulata Blanco* | | | 1 pack (150ml) / time, twice/day | Fluoxetine hydrochloride | | | | | 20-40 mg/ time, once/every other day |
| Wang et al.,2015 | | Yiqi Jianpi Bushen Decoction | Decoction | *Astragalus membranaceus（Fisch.） Bge.,Codonopsis pilosula (Franch.)Nannf.,Atractylodes macrocephala Koidz.,Bupleurum chinense DC. or Bupleurum scorzonerifolium Willd.,Cuscuta australis R.Br.,Epimedium brevicornu Maxim.,Psoralea corylifolia L.,Lycium barbarum L.* | | | 150-200ml / time, twice/day | Buzhong Yiqi Decotion Combine with Xiaoyao San | | | | | 150-200ml / time, twice/day |
| Liu et al.,2015a | | Shugan Yangxue Method | Decoction | *Bupleurum chinense DC. or Bupleurum scorzonerifolium Willd. ，Rehmannia glutinosa Libosch. ， Citrus medica L. or Citrus wilsonii Tanaka ，Citrus medica L. var. sarcodactylis Swingle ，Curcuma wenyujin Y. H. Chen et C. Ling（or Curcuma Longa L. or Curcuma kwangsiensis S. G. Lee et C. F. Liang or Curcuma phaeocaulis Val.) ，Paeonia lactiflora Pall. ，Ligusticum chuanxiong Hort. ，Angelica sinensis（Oliv.）Diels ，Albizia julibrissin Durazz.* | | | 1 pack / time, twice/day | VitaminC+MultivitaminB +Oryzanol +Adenosine Triphosphate | | | | | 1 dose(VC*0. 1 g +MultiVB*0.2g +Oryzanol * 20 mg +ATP*20 mg)/time, 3 times/day |
| Liu et al.,2015b | | Jianpi Jieyu Xiaopi Paste+ Psychological counseling | Plaster | *Astragalus membranaceus（Fisch.） Bge.，Codonopsis pilosula (Franch.)Nannf. ，Atractylodes macrocephala Koidz.，Angelica sinensis（Oliv.）Diels，Ligusticum chuanxiong Hort.，Poria cocos（Schw.）Wolf，Dioscorea opposita Thunb.，Achyranthes bidentata Bl.，Ziziphus jujuba Mill. var. spinosa（Bunge）Hu ex H. F. Chou，Albizia julibrissin Durazz.，Polygonum multiflorum Thunb.，Dimocarpus longan Lour.，Paeonia lactiflora Pall.，Paeonia lactiflora Pall.，Bupleurum chinense DC. or Bupleurum scorzonerifolium Willd.，Curcuma wenyujin Y. H. Chen et C. Ling（or Curcuma Longa L. or Curcuma kwangsiensis S. G. Lee et C. F. Liang or Curcuma phaeocaulis Val.) ，Aucklandia lappa Decne.，Citrus reticulata Blanco ，Gallus gallus domesticus Brisson ，Glycyrrhiza uralensis Fisch.，TESTUDINIS CARAPACIS ET PLASTRI COLLA* | | | N/A | Guipi Pill+Oryzanol Tablets+Psychological counseling | | | | | Guipi Pill* 1 pill, twice/day Erguwei*20 m g, 3 times/day; |
| Li, 2015 | | Buzhong Yiqi Decotion Combine with Xiaochaihu Decotion | Decoction | *Codonopsis pilosula (Franch.)Nannf.,Astragalus membranaceus（Fisch.） Bge.,Bupleurum chinense DC. or Bupleurum scorzonerifolium Willd.,Agrimonia pilosa Ledeb.,Atractylodes macrocephala Koidz.,Pinellia ternata（Thunb.） Breit.,Poria cocos（Schw.）Wolf,Curcuma wenyujin Y. H. Chen et C. Ling（or Curcuma Longa L. or Curcuma kwangsiensis S. G. Lee et C. F. Liang or Curcuma phaeocaulis Val.) ,Platycodon grandiflorum （Jacq.）A.DC.,Citrus reticulata Blanco ,Scutellaria baicalensis Georgi,Glycyrrhiza uralensis Fisch.,Ziziphus jujuba Mill.* | | | 1 pack / time, twice/day | ATP | | | | | 2 tablets/time, 3 times/day |
| Li and Cao.,2015 | | Invigorating spleen warming kidney and smoothing liver Decoction | Decoction | *Codonopsis pilosula (Franch.)Nannf.（or Codonopsis pilosula Nannf.var.modesta（Nannf.）L.T.Shen or Codonopsis tangshen Oliv） ，Atractylodes macrocephala Koidz. ，Poria cocos（Schw.）Wolf ，Angelica sinensis（Oliv.）Diels ，Ligusticum chuanxiong Hort. ，Paeonia lactiflora Pall. ，Rehmannia glutinosa Libosch. ，Curculigo orchioides Gaertn. ，Epimedium brevicornu Maxim. ，Bupleurum chinense DC. or Bupleurum scorzonerifolium Willd. ，Coptis chinensis Franch.（or Coptis deltoidea C.Y.Cheng et Hsiao,or Coptis teeta Wall. ， Gardenia jasminoides Ellis ，Curcuma wenyujin Y. H. Chen et C. Ling（or Curcuma Longa L. or Curcuma kwangsiensis S. G. Lee et C. F. Liang or Curcuma phaeocaulis Val.) ，Zingiber officinale Rosc ，Glycyrrhiza uralensis Fisch. ，Ziziphus jujuba Mill.* | | | 200ml / time, 3 times/day | Vitamin tablets + Oryzanol Tablets tablets | | | | | 1 tablet*2/time, 3 times/day. |
| Guo and Guo.,2015 | | Danzhi Xiaoyao tablet + Paxil Hydrochloride | Tablet | *Paeonia suffruticosa Andr. ,Gardenia jasminoides Ellis,Angelica sinensis（Oliv.）Diels,Paeonia lactiflora Pall.,Poria cocos（Schw.）Wolf,Atractylodes macrocephala Koidz.,Glycyrrhiza uralensis Fisch.,Mentha haplocalyx Briq. ,Bupleurum chinense DC. or Bupleurum scorzonerifolium Willd.* | | | 4 tablets/ time，3 times / day | Paxil Hydrochloride | | | | | 10-30mg / time, once/day |
| Gao and Pang,2015 | | Shugan Jianpi Method | Decoction | *Pinellia ternata（Thunb.） Breit.,Bambusa tuldoides Munro,Glycyrrhiza uralensis Fisch.,Bupleurum chinense DC. or Bupleurum scorzonerifolium Willd.,Citrus aurantium L.,Paeonia lactiflora Pall.,Zingiber officinale Rosc,Citrus reticulata Blanco* | | | 150ml / time, twice/day | | Fluoxetine hydrochloride capsules | | | | 20-40 mg/ time, once/every other day |
| Wang and Liu,2014 | | Jianpi Jieyu Xiaopi Paste+ Psychological counseling | Plaster | *Astragalus membranaceus（Fisch.） Bge.,Codonopsis pilosula (Franch.)Nannf.,Atractylodes macrocephala Koidz.,Angelica sinensis（Oliv.）Diels,Ligusticum chuanxiong Hort.,Poria cocos（Schw.）Wolf，Dioscorea opposita Thunb.，Achyranthes bidentata Bl. ，Ziziphus jujuba Mill. var. spinosa（Bunge）Hu ex H. F. Chou，Albizia julibrissin Durazz.，Polygonum multiflorum Thunb.，Dimocarpus longan Lour.，Paeonia lactiflora Pall.，Paeonia lactiflora Pall.，Bupleurum chinense DC. or Bupleurum scorzonerifolium Willd. ，Curcuma wenyujin Y. H. Chen et C. Ling（or Curcuma Longa L. or Curcuma kwangsiensis S. G. Lee et C. F. Liang or Curcuma phaeocaulis Val.) ，Aucklandia lappa Decne., Citrus reticulata Blanco ,Gallus gallus domesticus Brisson ,Glycyrrhiza uralensis Fisch.,TESTUDINIS CARAPACIS ET PLASTRI COLLA* | | | N/A | Guipi pill +Oryzanol Tablets+ Psychological counseling | | | | | Guipi Pill* 1 pill, twice/day Erguwei*20 m g, 3 times/day; |
| Teng et al.,2014 | | Buzhong Jiepi Decoction | Decoction | *Codonopsis pilosula (Franch.)Nannf.，Astragalus membranaceus（Fisch.） Bge.，Atractylodes macrocephala Koidz.，Glycyrrhiza uralensis Fisch.，Citrus reticulata Blanco ，Angelica sinensis（Oliv.）Diels，Cimicifuga heracleifolia Kom.，Bupleurum chinense DC. or Bupleurum scorzonerifolium Willd.，Pueraria lobata（Willd.）Ohwi ，Apatite (or Calcite)，Ostrea gigas Thunberg，* | | | 1 pack / time, twice/day | Oryzanol Tablets+VitaminB1 | | | | | 10mg*2 / time, 3 times/day |
| Niu et al.,2014 | | Bushen Shugan Decoction | Decoction | *Rehmannia glutinosa Libosch. ，Lycium barbarum L.，Rehmannia glutinosa Libosch. ，Scrophularia ningpoensis Hemsl. ，Ophiopogon japonicus (L. f.) Ker-Gawl. ， Angelica sinensis（Oliv.）Diels ，Ligusticum chuanxiong Hort. ，Bupleurum chinense DC. or Bupleurum scorzonerifolium Willd. ，Citrus aurantium L. ，Scutellaria baicalensis Georgi ，Coptis chinensis Franch.（or Coptis deltoidea C.Y.Cheng et Hsiao,or Coptis teeta Wall. ，Glycyrrhiza uralensis Fisch.* | | | 200ml / time, twice/day | ATP + Oryzanol Tablets | | | | | 20mg*2 / time, 3 times/day |
| Liu et al.,2014 | | Jianpi Jieyu Xiaopi Paste | Plaster | *Astragalus membranaceus（Fisch.） Bge.,Codonopsis pilosula (Franch.)Nannf.,Atractylodes macrocephala Koidz.,Angelica sinensis（Oliv.）Diels,Ligusticum chuanxiong Hort.,Poria cocos（Schw.）Wolf，Dioscorea opposita Thunb.，Achyranthes bidentata Bl. ，Ziziphus jujuba Mill. var. spinosa（Bunge）Hu ex H. F. Chou，Albizia julibrissin Durazz.，Polygonum multiflorum Thunb.，Dimocarpus longan Lour.，Paeonia lactiflora Pall.，Paeonia lactiflora Pall.，Bupleurum chinense DC. or Bupleurum scorzonerifolium Willd. ，Curcuma wenyujin Y. H. Chen et C. Ling（or Curcuma Longa L. or Curcuma kwangsiensis S. G. Lee et C. F. Liang or Curcuma phaeocaulis Val.) ，Aucklandia lappa Decne., Citrus reticulata Blanco ,Gallus gallus domesticus Brisson ,Glycyrrhiza uralensis Fisch.,TESTUDINIS CARAPACIS ET PLASTRI COLLA* | | | 15ml / time, twice/day | Guipi Pill +Oryzanol Tablets | | | | | Guipi Pill* 1 pill, twice/day Erguwei*20 m g, 3 times/day; |
| Dai et al.,2014 | | Buyi Pishen Decoction | Decoction | *Astragalus membranaceus（Fisch.） Bge.，Atractylodes macrocephala Koidz.，Pseudostellaria heterophylla（Miq.）Pax ex Pax et Hoffm.，Rehmannia glutinosa Libosch.，Dioscorea opposita Thunb.，Cornus officinalis Sieb. et Zucc，Rhodiola crenulata （Hook. f. et Thoms. ）H. Ohba，Dryopteris crassirhizoma Nakai，Citrus medica L. var. sarcodactylis Swingle ，Citrus reticulata Blanco ，Poria cocos（Schw.）Wolf，Ziziphus jujuba Mill. var. spinosa（Bunge）Hu ex H. F. Chou* | | | N/A | ATP + Oryzanol Tablets | | | | | ATP*60mg + Oryzanol*2 tablets , 3 times/day; |
| Zhao et al.,2013 | | Fufangteng Mixture | Mixtures | *Euonymus fortunei （Turcz.） Hand.-Mazz., Astragalus membranaceus（Fisch.） Bge.,Panax ginseng C.A.Mey.* | | | 15ml / time, twice/day | Weikangfu + ATP + Oryzanol Tablets | | | | | Weikangfu* 20mg, twice/day; ATP*20mg + Oryzanol*20mg , 3 times/day |
| Xu et al.,2013 | | Modified Naoxinkang | Decoction | *Panax ginseng C. A. Mey. ,Paeonia lactiflora Pall.,Spatholobus suberectus Dunn,Polygonum multiflorum Thunb. ,Ligusticum chuanxiong Hort.,Angelica sinensis（Oliv.）Diels,Pheretima aspergillum(E.Perrier）（or Pheretima vulgaris Chen or Pheretima guillelmi（Michaelsen）or Pheretima pectinifera Michaelsen）,Astragalus membranaceus（Fisch.） Bge.,Lycopodium japonicum Thunb.* | | | 100ml / time, twice/day | ATP | | | | | 1 tablet/ time, twice/day |
| Xu and Wang.,2013 | | Chaihu Combine with Longgu Muli Decoction | Decoction | *Bupleurum chinense DC. or Bupleurum scorzonerifolium Willd.，Scutellaria baicalensis Georgi ，Pinellia ternata（Thunb.） Breit. ，Panax ginseng C. A. Mey. ，Cinnamomum cassia Presl ，Rheum palmatum L.（or Rheum tanguticum Maxim. ex Bal£. or Rheum officinale Baill.） ，Apatite (or Calcite) ，Ostrea gigas Thunberg ，Amber ，Zingiber officinale Rosc ，Ziziphus jujuba Mill.* | | | 200ml / time, twice/day | Paxil + psychological counseling | | | | | 20-40 mg/ time, once/day |
| Sun , 2013 | | Panax quinquefolius L.+conventional therapy | Prepared Slices | *Panax quinquefolius L* | 6-10g Prepared Slices/ day | | | conventional therapy | | | | | N/A |
| Kim et al., 2013 | | Extract of P. ginseng | Capsule | *Panax ginseng C. A. Mey.* | T1：2g raw material/capsule, 4 capsules (250 mg)/time, twice/day | | | | | placebo | | | 4 capsules (250 mg)/time, twice/day |
|  |  |  |  |  | T2：1g raw material/capsule, 4 capsules (250 mg)/time, twice/day | | | | |  |  |  |  |

T: test group; C: control group; H: health group
